# Estrone‐α‐2‐Deoxy‐Glucoside as a Targeted Therapy for Triple‐Negative Breast Cancer: Aromatase Inhibition and Cytotoxicity

**DOI:** 10.1111/cbdd.70251

**Published:** 2026-01-30

**Authors:** Tzu‐Yu Huang, Meng‐Ru Wang, Feng‐Pai Chou, Sheng‐Cih Huang, Po‐Yun Hsiao, Tung‐Kung Wu

**Affiliations:** ^1^ Department of Biological Science and Technology National Yang Ming Chiao Tung University Hsin‐Chu Taiwan; ^2^ Center for Emergent Functional Matter Science National Yang Ming Chiao Tung University Hsinchu Taiwan; ^3^ Center for Intelligent Drug Systems and Smart Bio‐Devices (IDS^2^B) National Yang Ming Chiao Tung University Hsinchu Taiwan

**Keywords:** aromatase inhibitors, endocrine therapy, estradiol, estrone, steroidal glycoside, *trans*‐androsterone

## Abstract

Aromatase inhibitors (AIs) are vital in the treatment of estrogen‐dependent breast cancer, especially in postmenopausal women. In this study, a series of steroidal glycosides (SGs) derived from *trans*‐androsterone (*t*AND), estrone (E1), and estradiol (E2) were synthesized using a one‐pot multi‐enzyme glycosylation approach and structurally characterized via HPLC, MS, and NMR. Among the synthesized compounds, E1‐α‐2DG (**2b**) and E2‐α‐2DG (**3b**) demonstrated the most potent aromatase inhibition, with IC_50_ values of 0.101 ± 0.001 μM and 0.159 ± 0.009 μM, respectively. Molecular docking revealed that these glycosides form key hydrogen bonds with catalytic residues and the heme group of CYP19A1. In vitro cytotoxicity showed that E1‐α‐2DG selectively inhibited the growth of MCF‐7 and MDA‐MB‐231 breast cancer cells in a dose‐dependent manner, with the highest potency observed against triple‐negative MDA‐MB‐231 cells (IC_50_ = 20.46 ± 2.92 μM), while exhibiting no toxicity toward non‐cancerous HEK293 cells. These findings suggest that glycosylation enhances the pharmacological potential of steroidal scaffolds and highlights E1‐α‐2DG as a promising lead compound for the development of safer, dual‐function breast cancer therapies.

## Graphical abstract

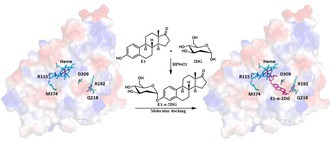

## 1 Introduction

Breast cancer remains the most commonly diagnosed cancer and the second leading cause of cancer‐related mortality, primarily due to its high metastatic potential (Chikara and Parang 2023). In 2022, approximately 20 million new cancer cases and 9.7 million deaths were reported, with nearly 90% of breast cancer deaths attributed to metastasis to the bone, liver, and brain (Bachmann et al. 2015; Bray et al. 2024; Giaquinto et al. 2022; Hess et al. 2006; Łukasiewicz et al. 2021; Rahman and Mohammed 2015; Weilbaecher et al. 2011). Breast cancer is classified into four subtypes based on estrogen receptor (ER), progesterone receptor (PR), epidermal growth factor 2 (ERBB2/HER2), and Ki‐67 expression: luminal A, luminal B, HER2‐enriched, and triple‐negative breast cancer (TNBC) (Ades et al. 2014; Burguin et al. 2021; Cruz‐Tapias et al. 2021; Cserni et al. 2021; Gao and Swain 2018; Loibl and Gianni 2017; Perou et al. 2000). These subtypes differ significantly in prognosis and therapeutic responsiveness, with luminal A showing the most favorable outcomes, while TNBC remains the most aggressive and therapeutically challenging subtype (Balma et al. 2022).

Aromatase inhibitors (AIs) are central to endocrine therapy for ER^+^ breast cancer, as they suppress estrogen biosynthesis by inhibiting aromatase, the enzyme responsible for converting androgens to estrogens (Lombardi 2002; Macedo et al. 2008). Based on their chemical structures and mechanisms of action, AIs are classified as steroidal or non‐steroidal. Steroidal AIs, such as exemestane, are androstenedione analogues that function as mechanism‐based, irreversible suicide inhibitors, offering high target specificity and sustained enzyme suppression but potentially retaining residual hormonal activity and steroid‐related side effects. In contrast, non‐steroidal AIs, including anastrozole and letrozole, inhibit aromatase reversibly via coordination with the heme iron of CYP19A1, generally providing favorable pharmacokinetics and minimal intrinsic hormonal activity, albeit with possible enzyme recovery and off‐target cytochrome P450 interactions. Despite their clinical success, the efficacy of current endocrine therapies is limited by resistance, adverse effects, and a lack of targeted options for ER^−^ subtypes such as TNBC.

Recent studies have explored steroid scaffolds such as *trans*‐androsterone (*t*AND), estrone (E1), and estradiol (E2) for developing novel anticancer drugs (Huang et al. 2015; Ozcan‐Sezer et al. 2019; Wan et al. 2019; Yang et al. 2023). However, the potential for hormone‐driven tumorigenesis necessitates structural modifications that retain anticancer efficacy while minimizing estrogenic activity. In this context, steroidal bioconjugates—compounds that chemically link steroid backbones to other functional groups—have emerged as a promising drug discovery strategy (Bansal and Suryan 2022; Moses et al. 2014). Among them, steroidal glycosides (SGs) combine the rigidity and membrane permeability of steroids with the hydrophilicity of sugars, resulting in enhanced bioavailability, solubility, and selectivity, along with synergistic therapeutic efficacy (Liu et al. 2024; Moses et al. 2014).

Building on our previous studies, where we demonstrated that glycosylated steroids like *t*AND, pregnenolone, and dehydroepiandrosterone (DHEA) glycoside reduced breast cancer cell viability and enhanced tamoxifen efficacy, we sought to extend these findings by investigating their potential as aromatase inhibitors (Chou et al. 2020; Chou et al. 2017; Liu et al. 2024). In this study, glycosylated derivatives of *t*AND, E1, and E2 were synthesized and assessed for their cytotoxicity against ER^+^ (MCF‐7) and TNBC (MDA‐MB‐231) cell lines, as well as for their ability to inhibit aromatase activity. Among the 10 derivatives tested, estrone‐α‐2‐deoxy‐glucoside (E1‐α‐2‐DG, **2b**) exhibited potent, dose‐dependent cytotoxicity against both cell lines, with IC_50_ values significantly lower than unmodified estrone. Notably, E1‐α‐2DG (**2b**) and E2‐α‐2DG (**3b**) showed the strongest aromatase inhibition, supported by molecular docking studies confirming their interactions with key active site residues, including Asp309 (D309) and the heme group. These findings suggest that glycosylation not only enhances the anticancer activity of steroid derivatives but also introduces effective aromatase inhibition, offering a dual mechanism to combat both ER^+^ and ER^−^ breast cancers. Thus, our work lays the foundation for the development of next‐generation, multifunctional therapeutics with potential clinical applications, especially for challenging subtypes like TNBC.

## 2 Results and Discussion

### 2.1 Synthesis of *Trans*‐Androsterone‐, Estrone‐, and Estradiol‐Glycosides

**FIGURE 1:**
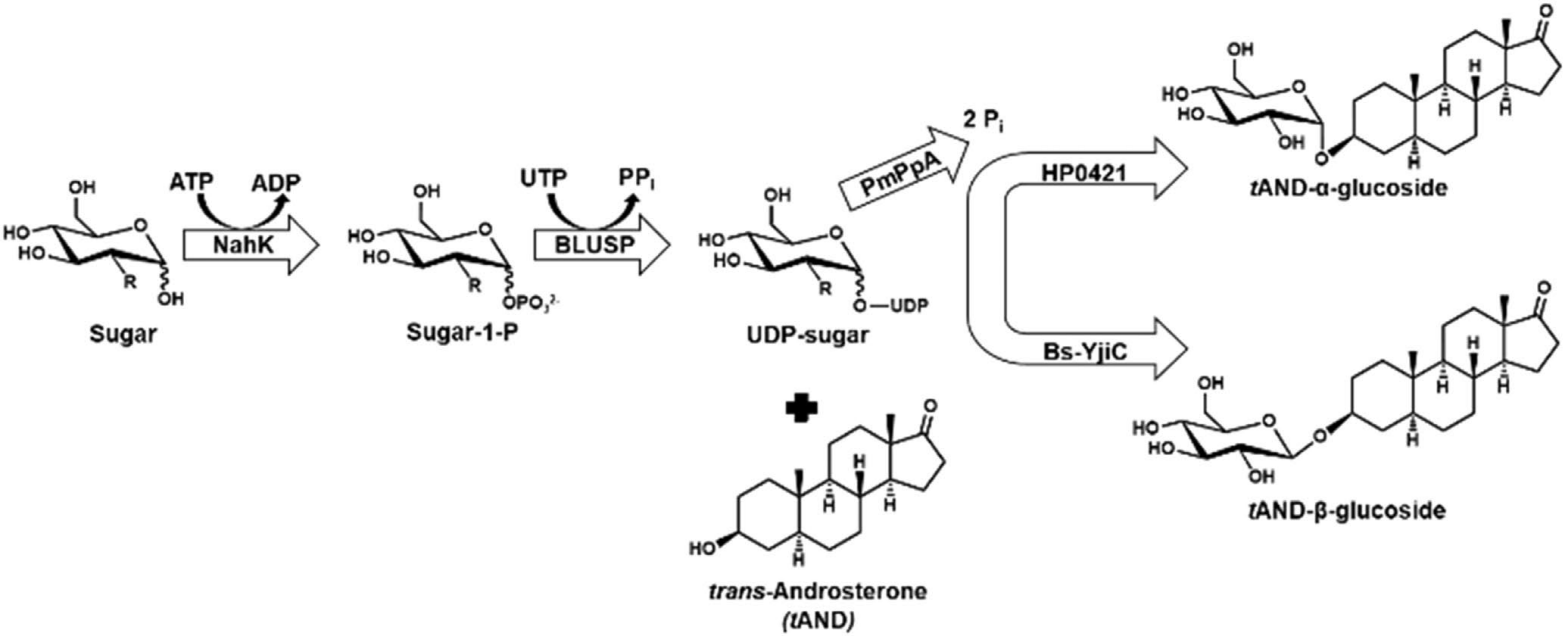
An in vivo one‐pot multi‐enzyme system for the synthesis of *trans*‐androsterone‐(α/β)‐Glc in *E. coli* cells.

**FIGURE 2:**
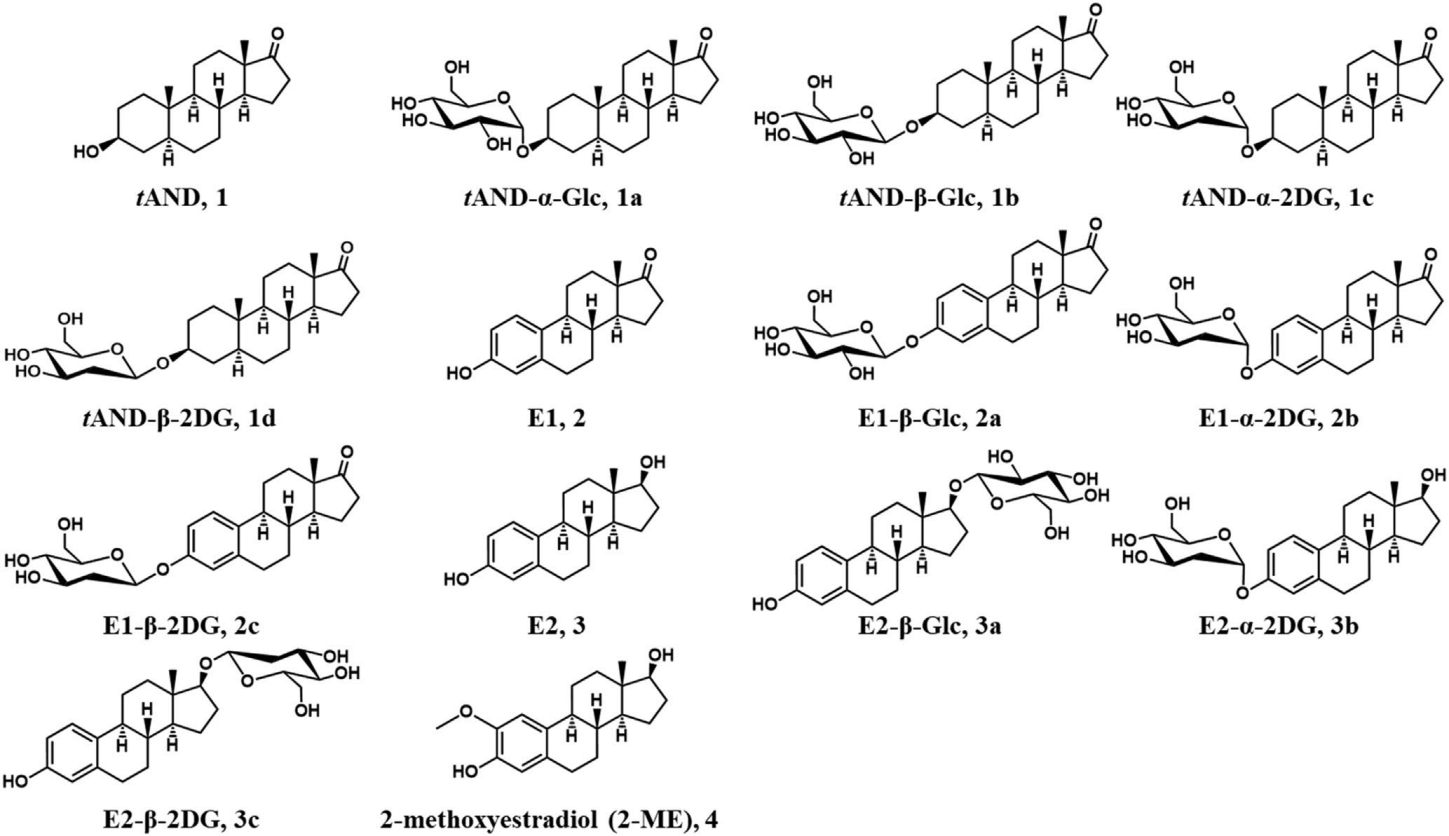
Structures of *t*AND (**1**), E1 (**2**), E2 (**3**), and *trans*‐androsterone‐, estrone‐, and estradiol‐glycosides (**1a**–**3c**), and 2‐methoxyestradiol (2‐ME, **4**).

Enantiomerically pure steroidal glycosides were enzymatically synthesized using an in vivo one‐pot multi‐enzyme system directly from glucose (Glc) or 2‐deoxy‐glucose (2‐DG) and *trans*‐androsterone, estrone, and estradiol. This system comprises four key enzymes, *N*‐acetylhexosamine 1‐kinase (NahK) and UDP‐glycopyrophosphorylase (BLUSP) from *Bifidobacterium longum* , inorganic pyrophosphatase (PmPpA) from *Pasteurella multocida* , as well as cholesterol‐α‐glucosyltransferase HP0421 from *Helicobacter pylori* for α‐glycosylation, and steroid‐β‐glycosyltransferase (Bs‐YjiC) from *Bacillus subtilis* for β‐glycosylation (Dai et al. 2017; Lebrun et al. 2006; Muthana et al. 2012; Nishimoto and Kitaoka 2007). This method efficiently converts inexpensive Glc or 2‐DG into UDP‐monosaccharides, eliminating the need for costly UDP‐sugar precursors and significantly reducing production costs. Inorganic pyrophosphate (PPi) generated during the reaction is hydrolyzed into two phosphate (Pi) molecules, driving the reaction equilibrium forward. The use of conformation‐selective glycosyltransferases ensures the synthesis of enantiomerically pure steroidal glycosides (Figure 1). The synthesized steroidal glycosides included *trans*‐androsterone‐(α/β)‐Glc (**1a**, **1b**), *trans*‐androsterone‐(α/β)‐2DG (**1c**, **1d**), estrone‐β‐Glc (**2a**), estrone‐(α/β)‐2DG (**2b**, **2c**), estradiol‐β‐Glc (**3a**), and estradiol‐(α/β)‐2DG (**3b**, **3c**) (Figure 2).

The products (**1a**–**3c**) were purified using column chromatography and high‐performance liquid chromatography (HPLC), and their molecular weights were initially characterized using high‐resolution electrospray ionization mass spectrometry (HR‐ESI‐MS). HR‐ESI‐MS revealed distinct *m/z* peaks used to determine the molecular masses of compounds **1a**–**3c**. Specifically, *t*AND‐α/β‐Glc (**1a** and **1b**) exhibited [M + H]^+^ and [M + HCOO]^−^ peaks at approximately *m/z* 453.2847 and 497.2744, respectively. *t*AND‐α/β‐2DG (**1c** and **1d**) showed a [M + Na]^+^ peak at *m/z* 459.2712. E1‐β‐Glc (**2a**) and E1‐α/β‐2DG (**2b** and **2c**) showed [M + HCOO]^−^ peaks at *m/z* 477.2120 and 461.2166, respectively. E2‐β‐Glc (**3a**) and E2‐α/β‐2DG (**3b** and **3c**) showed [M + HCOO]^−^ peaks at *m/z* 479.2291 and 463.2336, respectively.

The structures of compounds **1a**–**3c** were confirmed by ^1^H, ^13^C, HSQC, and HMBC nuclear magnetic resonance (NMR) spectroscopy to verify glycosylation (Figures S1–S10). For compounds **1a**‐**1d**, the ^1^H NMR spectra exhibited additional signals at δ_Η_ 3.09–5.04 ppm, with a 1:1 ratio between the steroid H9 proton (δ_Η_ 0.71 ppm) and the sugar H1′ proton (δ_Η_ 4.91–5.04 ppm). Additionally, the aglycone H3 proton experienced a downfield shift from δ_Η_ 3.49 to 3.59 ppm upon glycosylation. The ^13^C NMR spectra showed glycosylation‐induced shifts (Δδ_s_) of 5.0–8.0 ppm at C3, with the signal shifting from δ_C_ 70.3 to 75.2–77.7 ppm. A carbonyl signal at δ_C_ 222.5 ppm confirmed the presence of a C17 ketone. HMBC correlations between the sugar H1′ proton (δ_Η_ 4.37–5.04 ppm) and the aglycone C3 carbon (δ_C_ 75.2–77.7 ppm) verified the glycosidic bond.

For compounds **2a**–**2c**, the ^1^H NMR spectra displayed additional signals at δ_Η_ 3.02–5.52 ppm, with a 1:1 ratio between the olefinic H4 proton (δ_Η_ 6.74 ppm) and the sugar H1′ proton (δ_Η_ 4.82–5.52 ppm). The ^13^C NMR spectra showed minor glycosylation‐induced shifts (Δδ_s_) of 0.2–1.0 ppm, with the C3 signal shifting from δ_C_ 154.7 ppm to 154.9–155.5 ppm. A ketone signal at δ_C_ 220.1 ppm confirmed a C17 carbonyl. HMBC correlations between the sugar H1′ proton (δ_Η_ 4.82–5.52 ppm) and the C3 carbon (δ_C_ 154.89–155.50 ppm) supported glycosidic bond formation.

For compounds **3a** and **3c**, additional ^1^H NMR signals were observed at δ_Η_ 3.11–5.51 ppm, with a 1:1 ratio between the olefinic H4 proton (δ_Η_ 6.45 ppm) and the sugar H1′ proton (δ_Η_ 4.34–5.50 ppm). Glycosylation at C17 was confirmed by a downfield shift in the aglycone C17 carbon signal from δ_C_ 81.09 to 88.29–89.71 ppm (Δδ_s_ 7.0–9.0 ppm), with HMBC correlations between the H1′ proton (δ_Η_ 4.34–5.50 ppm) and the aglycone C17 carbon. For compound **3b**, glycosylation at C3 was verified by a downfield shift in the C3 carbon signal from δ_C_ 154.44 to 154.79 ppm and HMBC correlations between the sugar H1′ proton (δ_Η_ 5.50 ppm) and the aglycone C3 carbon.

The coupling constants of the sugar H1′ proton were used to determine the anomeric configuration of the monosaccharides. Compounds **1a**, **1c**, **2b**, and **3b** exhibited an α‐configuration (*J* = 2.59–3.85 Hz), while compounds **1b**, **1d**, **2a**, **2c**, **3a**, and **3c** exhibited a β‐configuration (*J* = 7.07–9.73 Hz). The presence of the C2′ carbon in 2‐DG derivatives was confirmed by characteristic ^13^C NMR chemical shifts: *t*AND‐α/β‐2DG at 37.9/39.5 ppm, E1‐α/β‐2DG at 38.3/35.8 ppm, and E2‐α/β‐2DG at 38.3/40.7 ppm. Except for the E2‐β‐Glc and E2‐β‐2DG derivatives, where glycosylation occurred at the steroid C17 position, all other glycosides were glycosylated at the steroid C3 position.

### 2.2 Cell Cytotoxicity Assays of Compounds 1a–3c on Breast Cancer Cell Lines

**FIGURE 3:**
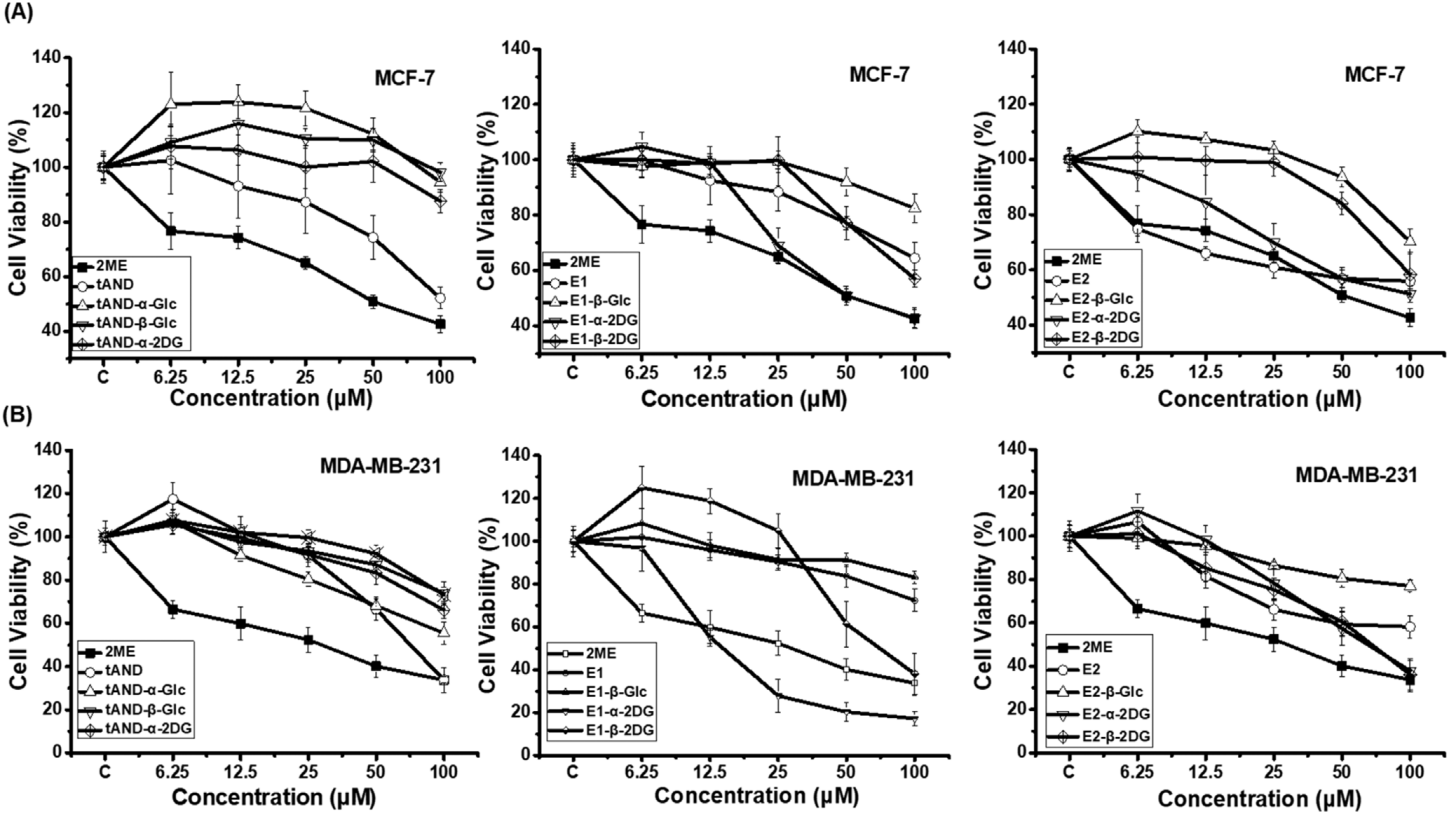
Cytotoxicity of compounds **1a**–**3c** and 2‐ME (**4**) at different concentrations on (A) MCF‐7 and (B) MDA‐MB‐231 cells.

Previous studies have utilized *t*AND as a scaffold to develop various derivatives targeting different tumor types, making them promising candidates for treating breast, liver, and lung, among others (Sandra et al. 2012; Yang et al. 2023). Estrone (E1) and estradiol (E2), as endogenous estrogens, contribute to the progression of hormone‐dependent cancers when present in excess. In addition, the estradiol derivative 2‐methoxyestradiol (2‐ME, **4**), is a natural metabolic product of estradiol. It can interact with estrogen receptors, inhibit hormone‐driven tumor growth, and induce apoptosis. Owing to its potent antiangiogenic and pro‐apototic properties, 2‐ME has been widely recognized as a promising anticancer candidate (Attia et al. 2024). To evaluate the inhibitory effects of compounds **1a**–**3c** on breast cancer cell growth, we tested their cytotoxicity against MCF‐7 and MDA‐MB‐231 breast cancer cells, using HEK293 cells as a control to assess selective toxicity. All three cell lines were treated with varying concentrations (0, 6.25, 12.5, 25, 50, and 100 μM) of compounds **1a**–**3c** and 2‐ME (**4**) for 48 h. The results showed that compounds **1a**–**1d** exhibited no inhibitory effects on the proliferation of MCF‐7 and MDA‐MB‐231 cells (IC_50_ > 100 μM) (Figure 3, Table S1). Among the estrone glycosides (**2a**–**2c**), E1‐α‐2DG (**2b**) significantly reduced cell viability, with IC_50_ values of 61.79 μM for MCF‐7 and 20.46 μM for MDA‐MB‐231. E1‐β‐2DG (**2c**) displayed moderate activity against MDA‐MB‐231 cells, with an IC_50_ of 68.27 μM, while the remaining estrone derivatives showed no detectable cytotoxic effects (IC_50_ > 100 μM).

Among the estradiol glycosides (**3a**–**3c**), only E2‐α‐2DG (**3b**) showed inhibitory activity against both MCF‐7 and MDA‐MB‐231 cell lines, with IC_50_ values of 88.41 μM and 62.96 μM, respectively. E2‐β‐2DG (**3c**) exhibited selective activity against MDA‐MB‐231 cells, with an IC_50_ of approximately 63.92 μM. Notably, none of the tested compounds (**1a**–**3c**) exhibited cytotoxicity toward normal HEK293 cells (IC_50_ > 100 μM). E1‐α‐2DG (**2b**) demonstrated dose‐dependent inhibition of MCF‐7 and MDA‐MB‐231 cells growth (Figure 3, Table S1). Furthermore, compared to 2‐ME (**4**), E1‐α‐2DG (**2b**) exhibited stronger inhibitory effects against MDA‐MB‐231 cells. These findings suggest that E1‐α‐2DG (**2b**) may serve as a promising lead compound for breast cancer therapy.

### 2.3 Inhibitory Effects of *t*AND‐/E1‐/E2‐Glycosides on Aromatase

**FIGURE 4:**
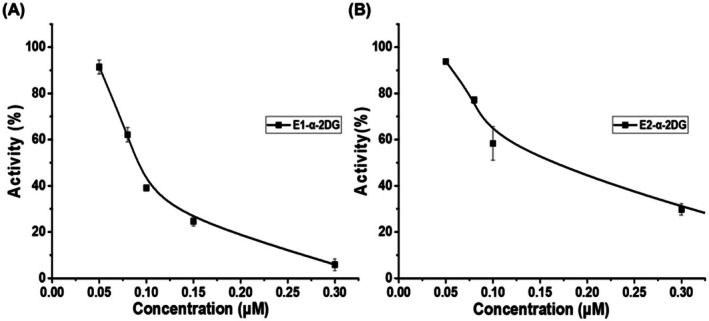
Inhibitory activity of (A) E1‐α‐2DG (**2b**) and (B) E2‐α‐2DG (**3b**) on aromatase (CYP19) at different concentrations.

To investigate the inhibitory effect of compounds **1a**–**3c** on aromatase (CYP19A1), an in vitro spectrofluorometric assay was performed using 7‐methoxy‐4‐trifluoromethyl coumarin (MFC) as the substrate and recombinant CYP19A1 protein as the enzyme. The inhibitory activities of compounds **1a**–**3c** on CYP19A1 were assessed by measuring the reduction in fluorescence intensity, which reflects the inhibition of MFC conversion to the fluorescent metabolite 7‐hydroxy trifluoromethyl coumarin (HFC) by aromatase. Exemestane, an FDA‐approved aromatase inhibitor commonly used in the treatment of hormone‐dependent breast cancers, served as the positive control. In vitro studies have shown that exemestane has an IC_50_ value of 0.0425 μM, providing an effective reference for inhibition assays (Lombardi 2002). The inhibitory activities of compounds **1a**–**3c** were evaluated at various concentrations (Table S2). Among all tested compounds, E1‐α‐2DG (**2b**) exhibited the most significant inhibitory effect, with an IC_50_ value of 0.101 ± 0.001 μM, followed by E2‐α‐2DG (**3b**), which had an IC_50_ of 0.159 ± 0.009 μM (Figure 4). Compared to exemestane, the inhibitory activities of E1‐α‐2DG (**2b**) and E2‐α‐2DG (**3b**) were approximately 2–3 times weaker. Although the overall inhibitory activities did not fully meet initial expectations, these results suggest that glycosylation of E1 and E2 with 2‐DG enhances their inhibitory effects compared to glycosylation with Glc.

### 2.4 Molecular Docking of E1‐α‐2DG With Aromatase

**FIGURE 5:**
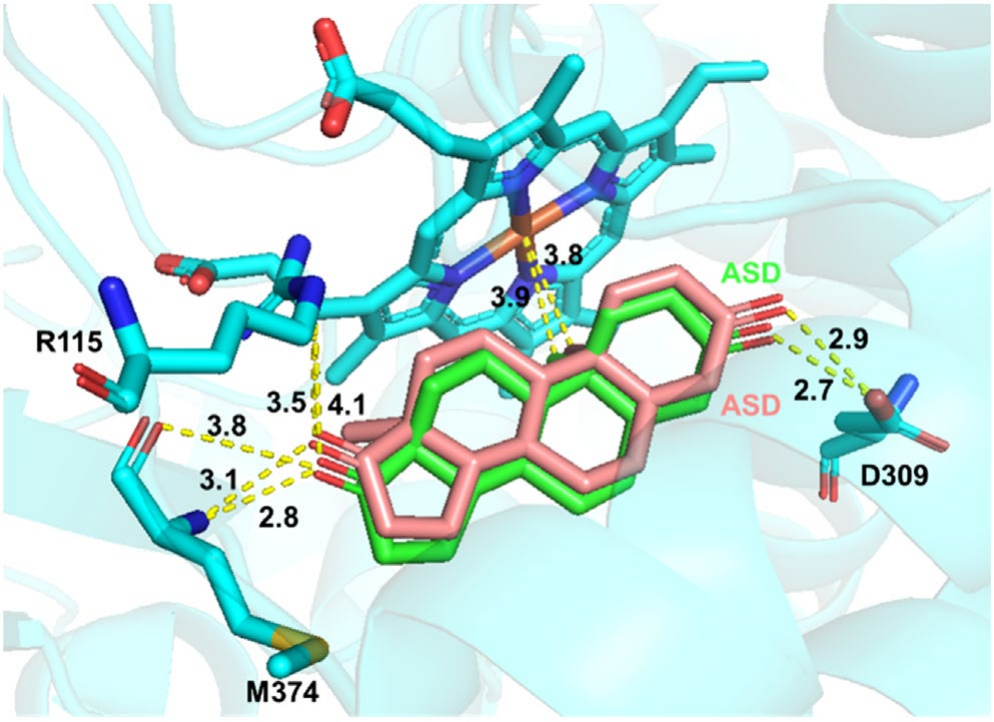
Overlay of the docking model of ASD (pink) with the X‐ray ligand ASD (green) within the active site of aromatase.

Aromatase inhibitors (AIs) play a crucial role in the treatment of estrogen‐dependent breast cancer in postmenopausal women. Therefore, understanding how substrates or inhibitors interact with the active site of aromatase is essential for optimizing AI‐based therapies. To investigate the structure–function relationship of aromatase and its inhibitors, we first performed molecular docking simulations using the aromatase/androstenedione (ASD) complex and compared the results with its reported crystal structure to validate the reliability of our docking approach (Ghosh et al. 2012). Subsequently, we conducted molecular docking simulations of the aromatase/E1‐α‐2DG complex and performed a comparative analysis with the aromatase/ASD crystal structure. Previous structural studies of the aromatase/ASD complex have identified four key catalytic site components: Arg115 (R115), Asp309 (D309), Met374 (M374), and the heme group. The C‐3 carbonyl group of ASD forms a hydrogen bond with D309, while the C‐17 carbonyl group interacts with R115 and M374. Additionally, the C‐19 methyl group of ASD engages in hydrophobic interactions with the heme moiety. In our docking model, ASD exhibited a binding mode that closely aligned with the X‐ray crystal structure of the aromatase/ASD complex, showing strong correspondence in terms of the active site geometry, spatial positioning, electrostatic interactions, hydrogen bonding, and hydrophobic contacts. These results confirm the reliability of our docking methodology (Figure 5).

**FIGURE 6:**
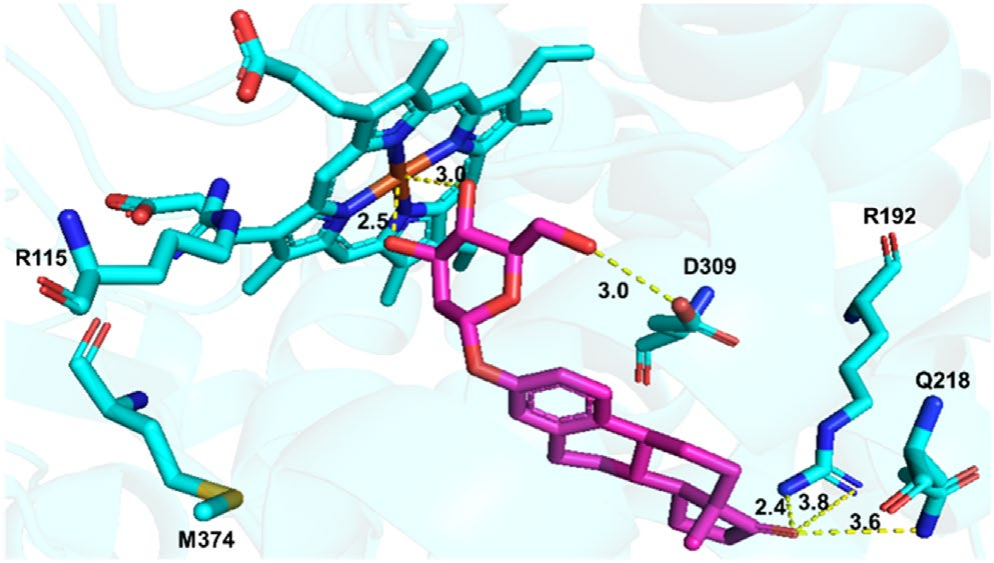
Molecular docking of E1‐α‐2DG with aromatase.

In the docking model of E1‐α‐2DG, binding energy analysis revealed a binding affinity of −117.45 kJ/mol. The C‐3′ hydroxyl group of E1‐α‐2DG formed a hydrogen bond with the heme group (2.5 Å), while the C‐6′ hydroxyl group interacted with D309 through a hydrogen bond (3.0 Å). Additionally, the C‐17 carbonyl group of E1‐α‐2DG formed hydrogen bonds with R192 (2.4 Å) and Q218 (2.4 Å), establishing further interactions with residues near the binding pocket (Figure 6).

**FIGURE 7:**
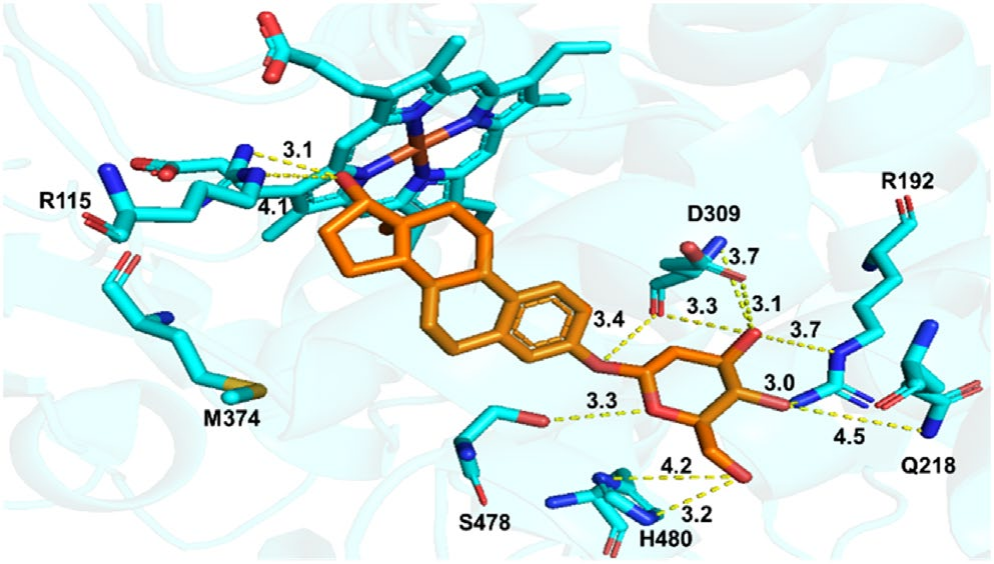
Molecular docking of E2‐α‐2DG with aromatase.

Molecular docking of E2‐α‐2DG yielded a binding energy of −114.17 kJ/mol and a binding orientation distinct from that of E1‐α‐2DG. Within the aromatase binding cavity, the C‐17 hydroxyl group of E2‐α‐2DG formed a hydrogen bond with R115 (3.1 Å), and the C‐3′ hydroxyl group formed a hydrogen bond with D309 (3.1 Å). Furthermore, E2‐α‐2DG engaged in additional interactions, including hydrogen bonding between its C‐1′ hydroxyl group and S478 (2.7 Å), as well as between its C‐4′ hydroxyl group and R192 (3.0 Å) (Figure 7).

Comparison of the docking results for E1‐α‐2DG or E2‐α‐2DG suggests that their steroidal cores retain a binding orientation similar to that of ASD, particularly with respect to interactions with the catalytic residue D309 and the region proximal to the heme group. The sugar moiety primarily contributes additional peripheral hydrogen‐bonding interactions (e.g., with residues such as R192 and Q218), which help enhance the overall stability of the ligand within the active site. In addition, differences in the C17 functional groups between E1‐α‐2DG and E2‐α‐2DG (carbonyl vs. hydroxyl group) may lead to variations in molecular conformation and hydrogen‐bonding networks, resulting in slightly different binding poses observed in the docking simulations.

Steroidal aromatase inhibitors, such as exemestane, are a mainstay in the treatment of estrogen‐dependent breast cancer due to their ability to irreversibly suppress estrogen synthesis and inhibit tumor growth. However, long‐term use of these agents is frequently associated with adverse effects—including bone demineralization, arthralgia, myalgia, cardiovascular complications, and urogenital atrophy—that not only compromise patient quality of life but may also reduce treatment compliance (Asten et al. 2014; Bell et al. 2020; Blanter et al. 2024; Geisler 2011; Heery et al. 2020; Lombardi 2002; Zarkavelis et al. 2016). These limitations underscore the urgent need for alternative or next‐generation AIs that offer improved therapeutic profiles with reduced toxicity. In this context, our development of steroidal glycosides (SGs) as novel aromatase inhibitors presents a promising avenue. By leveraging the pharmacological advantages of glycosylation—such as enhanced water solubility, increased metabolic stability, and prolonged systemic retention—SGs may overcome the bioavailability issues common to many steroidal drugs. Furthermore, SGs exhibit potential dual‐function anticancer activity: in addition to inhibiting aromatase‐mediated estrogen synthesis, they may exert intrinsic cytotoxic effects through mechanisms such as disruption of glucose metabolism and depletion of NADPH, both of which are critical for cancer cell survival. This multifaceted mode of action, combined with a degree of selectivity toward cancer cells, suggests that SGs may reduce off‐target toxicity and the systemic side effects often seen with traditional AIs.

Nevertheless, important challenges remain. Structural modifications resulting from glycosylation may compromise binding affinity to aromatase, rendering SGs less potent than clinically approved agents such as exemestane. Moreover, their glycosidic bonds may be susceptible to enzymatic hydrolysis by endogenous glycosidases, potentially leading to reduced efficacy or inconsistent pharmacological outcomes. There is also a need to evaluate the risk of off‐target effects and interactions with metabolic enzymes to ensure therapeutic safety. Despite these potential limitations, the development of SGs represents a novel and rational strategy for advancing endocrine therapy. The incorporation of sugar moieties into the steroidal framework not only provides an opportunity to fine‐tune pharmacokinetic and pharmacodynamic properties but also opens the door to multi‐targeted therapeutic interventions. Further preclinical and clinical studies are warranted to optimize their design, assess their in vivo efficacy, and establish their safety profile. Overall, this research highlights SGs as compelling candidates in the ongoing search for safer and more effective therapies for hormone‐dependent breast cancer.

## 3 Conclusion

This study demonstrates that glycosylation is an effective strategy for enhancing the bioactivity of steroidal compounds such as *t*AND, E1, and E2. Among the synthesized derivatives, E1‐α‐2DG and E2‐α‐2DG exhibited both selective cytotoxicity against MDA‐MB‐231 breast cancer cells (IC_50_ = 20.46 ± 2.92 μM) and potent aromatase inhibition (IC_50_ = 0.101 and 0.159 μM). Using a one‐pot multi‐enzyme system, 10 steroidal glycosides were synthesized and characterized, with regioselective glycosylation at the C‐3 and C‐17 hydroxyl groups. Molecular docking confirmed key interactions with aromatase catalytic residues, supporting their mechanism of inhibition. Future studies will focus on elucidating the structure–function‐mechanism relationships of E1‐α‐2DG through gene expression profiling of CYP19A1, apoptosis‐related markers, and glucose metabolism‐associated genes. In parallel, we will optimize glycoside structures to improve stability and efficacy (e.g., acetylation or sulfation), evaluate in vivo antitumor activity, enhance resistance to glycosidase‐mediated degradation, and expand anticancer screening across multiple tumor types. These findings position steroidal glycosides as promising candidates for hormone‐dependent and triple‐negative breast cancer therapy.

## Author Contributions

Tung‐Kung Wu conceptualization, supervision, writing – original draft, writing – review and editing. Tzu‐Yu Huang, Meng‐Ru Wang and Po‐Yun Hsiao performed enzymatic synthesis, bioassay and molecular docking studies. Feng‐Pai Chou performed the cellular bioassay. Sheng‐Cih Huang performed compound structural analysis. All authors gave final approval for publication and agreed to be held accountable for the work performed therein.

## Funding

This work was supported by the Ministry of Science and Technology, Taiwan, MOST 107‐2113‐M‐009‐009, MOST 110‐2113‐M‐A49‐017, MOST 112‐2113‐M‐A49‐008.

## Disclosure

Entry for the Table of Contents: Estrone‐α‐2DG, synthesized via a one‐pot multi‐enzyme system, selectively inhibits aromatase (IC50 = 0.101 μM) and triple‐negative breast cancer cells (IC50 = 20.46 μM), offering a dual‐function therapeutic scaffold.

## Conflicts of Interest

The authors declare no conflicts of interest.

## Supporting information


**Figure S1:** NMR of *t*AND‐α‐Glc.
**Figure S2:** NMR of *t*AND‐β‐Glc.
**Figure S3:** NMR of *t*AND‐α‐2DG.
**Figure S4:** NMR of *t*AND‐β‐2DG.
**Figure S5:** NMR of E1‐β‐Glc.
**Figure S6:** NMR of E1‐α‐2DG.
**Figure S7:** NMR of E1‐β‐2DG.
**Figure S8:** NMR of E2‐β‐Glc.
**Figure S9:** NMR of E2‐α‐2DG.
**Figure S10:** NMR of E2‐β‐2DG.
**Table S1:** Cell viability of tAND (1), E1 (2), E2 (3), 2‐ME (4), and trans‐androsterone‐, estrone‐, and estradiol‐glycoside (1a–3c) against different breast cancer cell lines.
**Table S2:** Inhibitory activities of tAND (1), E1 (2), E2 (3), 2‐ME (4), and trans androsterone‐, estrone‐, and estradiol‐glycoside (1a–3c) against aromatase CYP19.

## Data Availability

The data supporting the findings of this study are available on request from the corresponding authors.
