# Supplementary material for: Estrone‐α‐2‐Deoxy‐Glucoside as a Targeted Therapy for Triple‐Negative Breast Cancer: Aromatase Inhibition and Cytotoxicity
Source: Chem Biol Drug Des. 2026 Jan 30;107(2):e70251. doi: 10.1111/cbdd.70251 (PMC12857250; doi:10.1111/cbdd.70251)
Supplement: Supplementary file 1 — Figure S1: NMR of tAND‐α‐Glc. Figure S2: NMR of tAND‐β‐Glc. Figure S3: NMR of tAND‐α‐2DG. Figure S4: NMR of tAND‐β‐2DG. Figure S5: NMR of E1‐β‐Glc. Figure S6: NMR of E1‐α‐2DG. Figure S7: NMR of E1‐β‐2DG. Figure S8: NMR of E2‐β‐Glc. Figure S9: NMR of E2‐α‐2DG. Figure S10: NMR of E2‐β‐2DG. Table S1: Cell viability of tAND (1), E1 (2), E2 (3), 2‐ME (4), and trans‐androsterone‐, estrone‐, and estradiol‐glycoside (1a–3c) against different breast cancer cell lines. Table S2: Inhibitory activities of tAND (1), E1 (2), E2 (3), 2‐ME (4), and trans androsterone‐, estrone‐, and estradiol‐glycoside (1a–3c) against aromatase CYP19. [file CBDD-107-e70251-s001.pdf]

Supplementary Information for:

Estrone-α-2-Deoxy-Glucoside as a Targeted Therapy for Triple-Negative Breast Cancer: Aromatase Inhibition and Cytotoxicity

Tzu-Yu Huang¶[a], Meng-Ru Wang¶[a], Feng-Pai Chou[a], Sheng-Cih Huang[a], Po-Yun Hsiao[a], and Tung-Kung Wu*[a,b,c]

Experimental Section

Synthesis and NMR Data of *trans*-Androsterone, Estrone, and Estradiol Derivatives. 3-10

In vitro Aromatase Inhibitory Assays. 10-11

Cell Cytotoxicity Assays of *trans*-Androsterone, Estrone, and Estradiol Derivative. 11-12

Molecular Docking. 12

Figures

Fig. S1 NMR of *t*AND-α-Glc. 13-16

Fig. S2 NMR of *t*AND-β-Glc. 17-20

Fig. S3 NMR of *t*AND-α-2DG. 21-23

Fig. S4 NMR of *t*AND-β-2DG. 24-26

Fig. S5 NMR of E1-β-Glc. 27-29

Fig. S6 NMR of E1-α-2DG. 30-32

Fig. S7 NMR of E1-β-2DG. 33-35

Fig. S8 NMR of E2-β-Glc. 36-38

Fig. S9 NMR of E2-α-2DG. 39-41

Fig. S10 NMR of E2-β-2DG. 42-44

Table

Table 1. Cell viability of *t*AND (**1**), E1 (**2**), E2 (**3**), 2-ME (**4**), and *trans*-androsterone-, estrone-, and estradiol-glycoside (**1a**–**3c**) against different breast cancer cell lines. 45

EXPERIMENTAL SECTION

Synthesis of *trans*-Androsterone, Estrone, and Estradiol Derivatives Using Whole Cell Biocatalysts

The synthesis of *trans*-androsterone, estrone, and estradiol derivatives was carried out following previously reported method.[1] *E. coli* BL21(DE3) cells were co-transformed with pCOLADuet-PmPpA-NahK and either pETDuet-BLUSP-HP0421 or pETDuet-BLUSP-Bs-YjiC plasmids. Protein expression was induced with 0.2 mM IPTG at 28 °C overnight. The harvested cells were re-suspended in M9 salts supplemented with 1 mM sterol acceptor (dissolved in 0.03% DMSO) and 15 mM sugar donors, and the reaction mixture was incubated for 12–16 hours at 37 °C.

Following the reaction, the mixtures were extracted three times with ethyl acetate, and the organic layer was concentrated under vacuum. Crude products were purified via column chromatography using an ethyl acetate-ethanol ratio (7:1) solvent system. Product purity was confirmed via HPLC on an ACCHROM Unitary C18 column (10 mm × 250 mm, 5 μm) with a gradient elution of water (A) and methanol (B) at a rate of 1.5–2.0 mL/min. The gradient program was as follows: 0-7 min, 60 % to 80 % B; 7-10 min, 80 % to 100 % B; 100 % B maintained for 10 min; followed by re-equilibration at 60 % B for 10 min. The molecular structures of the purified products were characterized using HR-ESI-MS in both positive and negative modes, along with NMR analyses, including ^{1}H, ^{13}C, DEPT, HSQC, and HMBC.

***t*AND-α-Glc (1a):** Chemical Formula: $C_{25}H_{40}O_7$, Molecular Weight: 452.5880

*t*AND-α-Glc (**1a**) exhibited $[M + H]^+$ theoretical and experimental peaks at approximately *m*/*z* = 453.2847 and *m*/*z* = 453.2847, respectively.

^{1}H NMR (700 MHz, methanol-*d*$_4$, CD_3OD): δ 4.91 (d, *J* = 3.9 Hz, 1H, H-1’), 3.78 – 3.74 (m, 1H, H-6’a), 3.66 – 3.62 (m, 2H, H-5’, H-6’b), 3.62 – 3.55 (m, 2H, H-3, H-

3’), 3.33 (dd, *J* = 9.8, 3.7 Hz, 1H, H-2’), 3.25 (t, *J* = 9.1 Hz, 1H, H-4’), 2.40 (dd, *J* = 19.3, 8.9, 1.1 Hz, 1H, H-16a), 2.04 (m, *J* = 19.3, 9.1 Hz, 1H, H-16b), 1.95 – 1.86 (m, 2H, H-2a, H-15a), 1.81 (dq, *J* = 12.9, 3.5 Hz, 1H, H-7a), 1.77 – 1.70 (ddt, 2H, H-1a, H-12a), 1.68 – 1.63 (m, 2H, H-4a, H-11a), 1.62 – 1.55 (qd, 1H, H-8), 1.55 – 1.49 (m, 1H, H-15b), 1.47 – 1.39 (m, 2H, H-2b, H-4b), 1.39 – 1.25 (m, 4H, H-6a, H-6b, H-11b, H-14), 1.23 – 1.13 (m, 2H, H-5, H-12b), 1.06 – 0.93 (m, 2H, H-1b, H-7b), 0.86 (s, 3H, H-19), 0.85 (s, 3H, H-18), 0.72 (ddd, *J* = 12.4, 10.4, 4.1 Hz, 1H, H-9).

^{13}C NMR (175 MHz, methanol-d_4, CD_3OD): δ_c 222.56 (C-17), 96.71 (C-1’), 76.36 (C-3), 73.64 (C-3’), 72.20 (C-5’), 72.01 (C-2’), 70.50 (C-4’), 61.32 (C-6’), 54.42 (C-9), 51.28 (C-14), 47.66 (C-13), 44.90 (C-5), 36.60 (C-1), 35.59 (C-4), 35.45 (C-10), 35.23 (C-16), 34.92 (C-8), 31.36 (C-12), 30.63 (C-7), 28.19 (C-6), 26.89 (C-2), 21.25 (C-15), 20.13 (C-11), 12.72 (C-18), 11.21 (C-19).

***t*AND-β-Glc (1b):** Chemical Formula: $C_{25}H_{40}O_7$, Molecular Weight: 452.5880

*t*AND-β-Glc (**1b**) exhibited [M + HCOO]$^-$ theoretical and experimental peaks at approximately *m*/*z* = 497.2756 and *m*/*z* = 497.2744, respectively.

^{1}H NMR (700 MHz, methanol-d_4, CD_3OD): δ 4.37 (d, *J* = 7.8 Hz, 1H, H-1’), 3.83 (dd, *J* = 11.9, 2.0 Hz, 1H, H-6’a), 3.69 (td, *J* = 11.3, 5.6 Hz, 1H, H-3), 3.63 (dd, *J* = 11.8, 5.2 Hz, 1H, H-6’b), 3.32 (t, *J* = 8.6 Hz, 1H, H-3’), 3.27 – 3.20 (m, 2H, H-4’, H-5’), 3.11 (dd, *J* = 9.2, 7.8 Hz, 1H, H-2’), 2.40 (dd, *J* = 19.4, 8.6 Hz, 1H, H-16a), 2.04 (dt, *J* = 19.2, 9.1 Hz, 1H, H-16b), 1.92 (ddd, *J* = 12.3, 8.5, 5.9 Hz, 1H, H-15a), 1.89 – 1.84 (m, 1H, H-2a), 1.81 (dq, *J* = 12.9, 3.5 Hz, 1H, H-7a), 1.76 – 1.67 (m, 3H, H-1a, H-4a, H-12a), 1.67 – 1.62 (m, 1H, H-11a), 1.62 – 1.46 (m, 3H, H-2b, H-8, H-15b), 1.39 – 1.25 (m, 5H, H-4b, H-6a, H-6b, H-11b, H-14), 1.19 (td, *J* = 13.1, 4.2 Hz, 1H, H-12b), 1.12 (ddq, *J* = 12.9, 7.8, 3.1 Hz, 1H, H-5), 1.05 – 0.96 (m, 2H, H-1b, H-7b), 0.85 (s, 3H, H-19), 0.85 (s, 3H, H-18), 0.71 (ddd, *J* = 12.3, 10.4, 4.1 Hz, 1H, H-9).

^{13}C NMR (175 MHz, methanol-*d*4, CD_3OD): δ_c 222.67 (C-17), 100.79 (C-1’), 77.70 (C-3), 76.63 (C-3’), 76.40 (C-5’), 73.66 (C-2’), 70.22 (C-4’), 61.33 (C-6’), 54.43 (C-9), 51.27 (C-14), 47.65 (C-13), 44.57 (C-5), 36.76 (C-1), 35.44 (C-10), 35.23 (C-16), 34.90 (C-8), 33.90 (C-4), 31.35 (C-12), 30.63 (C-7), 28.97 (C-2), 28.25 (C-6), 21.25 (C-15), 20.12 (C-11), 12.73 (C-18), 11.21 (C-19).

***t*AND-α-2DG (1c):** Chemical Formula: $C_{25}H_{40}O_6$, Molecular Weight: 436.5890

*t*AND-α-2DG (**1c**) showed $[M + Na]^+$ theoretical and experimental peaks at approximately *m*/*z* = 459.2717 and *m*/*z* = 459.2708, respectively.

^1H NMR (700 MHz, methanol-*d*4, CD_3OD): δ 5.04 (d, *J* = 3.6 Hz, 1H, H-1’), 3.80 (ddd, *J* = 11.7, 8.9, 5.0 Hz, 1H, H-3’), 3.77 (dd, *J* = 11.7, 2.5 Hz, 1H, H-6’a), 3.66 (dd, *J* = 11.7, 5.6 Hz, 1H, H-6’b), 3.60 – 3.53 (m, 2H, H-3, H-5’), 3.19 (t, *J* = 9.4 Hz, 1H, H-4’), 2.40 (dd, *J* = 19.3, 8.8 Hz, 1H, H-16a), 2.03 (dt, *J* = 18.9, 9.1 Hz, 1H, H-16b), 1.96 (dd, *J* = 12.7, 5.1 Hz, 1H, H-2’a), 1.92 (ddd, *J* = 12.2, 8.8, 5.9 Hz, 1H, H-15a), 1.86 (d, *J* = 10.3 Hz, 1H, H-2a), 1.80 (dq, *J* = 12.9, 3.5 Hz, 1H, H-7a), 1.76 – 1.69 (m, 2H, H-1a, H-12a), 1.67 – 1.48 (m, 4H, H-2’b, H-4a, H-8, H-15b), 1.38 – 1.25 (m, 6H, H-2b, H-4b, H-6a, H-6b, H-11b, H-14), 1.22 – 1.12 (m, 2H, H-5, H-12b), 0.98 (m, 2H, H-1b, H-7b), 0.84 (s, 6H, H-18, H-19), 0.71 (ddd, *J* = 12.4, 10.4, 4.1 Hz, 1H, H-9).

^{13}C NMR (175 MHz, methanol-*d*4, CD_3OD): δ_c 222.58 (C-17), 95.02 (C-1’), 75.21 (C-3), 72.63 (C-5’), 72.06 (C-4’), 68.51 (C-3’), 61.52 (C-6’), 54.49 (C-9), 51.33 (C-14), 47.68 (C-13), 44.91 (C-5), 37.91 (C-2’), 36.66 (C-1), 35.67 (C-4), 35.48 (C-10), 35.29 (C-16), 34.96 (C-8), 31.41 (C-12), 30.67 (C-7), 28.24 (C-6), 27.15 (C-2), 21.31 (C-15), 20.19 (C-11), 12.82 (C-18), 11.31 (C-19).

***t*AND-β-2DG (1d):** Chemical Formula: $C_{25}H_{40}O_6$, Molecular Weight: 436.5890

*t*AND-β-2DG (**1d**) showed $[M + Na]^+$ theoretical and experimental peaks at

approximately *m*/*z* = 459.2717 and *m*/*z* = 459.2712, respectively.

^{1}H NMR (700 MHz, methanol-d_4, CD_3OD): δ 4.68 (dd, *J* = 9.7, 1.9 Hz, 1H, H-1'), 3.83 (dd, *J* = 11.8, 2.3 Hz, 1H, H-6'a), 3.71 (tq, *J* = 11.1, 5.4, 4.8 Hz, 1H, H-3), 3.65 (dd, *J* = 11.8, 5.7 Hz, 1H, H-6'b), 3.53 (ddd, *J* = 11.8, 8.5, 5.1 Hz, 1H, H-3'), 3.20 – 3.10 (m, 2H, H-4', H-5'), 2.40 (dd, *J* = 19.3, 8.7 Hz, 1H, H-16a), 2.07 – 1.99 (m, 2H, H-2'a, H-16b), 1.95 – 1.89 (m, 1H, H-15a), 1.85 (d, *J* = 11.5 Hz, 1H, H-2a), 1.81 (dq, *J* = 12.8, 3.5 Hz, 1H, H-7a), 1.74 – 1.69 (m, 2H, H-1a, H-12a), 1.65 (ddq, *J* = 12.8, 5.7, 2.9, 2.4 Hz, 2H, H-4a, H-11a), 1.62 – 1.49 (m, 2H, H-8, H-15b), 1.49 – 1.40 (m, 2H, H-2'b, H-2b), 1.38 – 1.25 (m, 4H, H-6a, H-6b, H-11, H-14), 1.25 – 1.16 (m, 2H, H-4b, H-12b), 1.16 – 1.08 (m, 1H, H-5), 1.04 – 0.96 (m, 2H, H-1b, H-7b), 0.84 (s, 6H, H-18, H-19), 0.71 (ddd, *J* = 12.4, 10.4, 4.1 Hz, 1H, H-9).

^{13}C NMR (175 MHz, methanol-d_4, CD_3OD): δ_c 222.59 (C-17), 97.42 (C-1'), 76.95 (C-3), 76.59 (C-5'), 71.73 (C-4'), 71.19 (C-3'), 61.58 (C-6'), 54.49 (C-9), 51.31 (C-14), 47.68 (C-13), 44.58 (C-5), 39.51 (C-2'), 36.77 (C-1), 35.47 (C-10), 35.27 (C-16), 34.93 (C-8), 34.09 (C-4), 31.40 (C-12), 30.67 (C-7), 29.02 (C-6), 28.29 (C-2), 21.30 (C-15), 20.16 (C-11), 12.79 (C-18), 11.25 (C-19).

E1-β-Glc (2a): Chemical Formula: $C_{24}H_{32}O_7$, Molecular Weight: 432.5130

E1-β-Glc (**2a**) displayed $[M + HCOO]^-$ theoretical and experimental peaks at approximately *m*/*z* = 477.2130 and *m*/*z* = 477.2120, respectively.

^{1}H NMR (700 MHz, methanol-d_4, CD_3OD): δ 7.18 (d, *J* = 8.6 Hz, 1H, H-1), 6.85 (dd, *J* = 8.6, 2.7 Hz, 1H, H-2), 6.80 (d, *J* = 2.7 Hz, 1H, H-4), 4.82 (d, *J* = 7.1 Hz, 1H, H-1'), 3.86 (d, *J* = 2.2 Hz, 1H, H-6'a), 3.67 (dd, *J* = 12.1, 5.5 Hz, 1H, H-6'b), 3.46 – 3.34 (m, 4H, H-2', H-3', H-4', H-5'), 2.88 – 2.84 (m, 2H, H-6a, H-6b), 2.47 (dd, *J* = 19.1, 8.7 Hz, 1H, H-16a), 2.41 – 2.37 (m, 1H, H-11a), 2.25 – 2.20 (m, 1H, H-9), 2.12 (dt, *J* = 18.7, 8.9 Hz, 1H, H-16b), 2.05 (ddd, *J* = 11.7, 8.8, 5.1 Hz, 1H, H-15a), 2.01

(ddt, J = 12.6, 5.5, 2.5 Hz, 1H, H-7a), 1.89 – 1.84 (m, 1H, H-12a), 1.64 (tt, J = 11.7, 8.8 Hz, 1H, H-15b), 1.59 – 1.51 (m, 2H, H-8, H-14), 1.51 – 1.37 (m, 3H, H-7b, H-11b, H-12b), 0.90 (s, 3H, H-18).

^{13}C NMR (175 MHz, methanol-d_4, CD_3OD): δ_c 222.14 (C-17), 155.50 (C-3), 137.32 (C-5), 133.43 (C-10), 125.65 (C-1), 116.28 (C-4), 113.74 (C-2), 100.94 (C-1'), 76.52 (C-5'), 76.43 (C-3'), 73.37 (C-2'), 69.87 (C-4'), 60.98 (C-6'), 50.04 (C-14), 47.71 (C-13), 43.79 (C-9), 38.20 (C-8), 35.14 (C-16), 31.21 (C-12), 29.05 (C-6), 26.08 (C-7), 25.47 (C-11), 20.91 (C-15), 12.70 (C-18).

E1-α-2DG (2b): Chemical Formula: $C_{24}H_{32}O_6$, Molecular Weight: 416.5140

E1-α-2DG (**2b**) displayed [M + HCOO]$^-$ theoretical and experimental peaks at approximately m/z = 461.2181 and m/z = 461.2166, respectively.

^{1}H NMR (700 MHz, DMSO-d_6, $(CD_3)_2SO$): δ 7.13 (d, J = 8.6 Hz, 1H, H-1), 6.78 (dd, J = 8.6, 2.6 Hz, 1H, H-2), 6.74 (d, J = 2.6 Hz, 1H, H-4), 5.52 (d, J = 2.7 Hz, 1H, H-1'), 4.89 (d, J = 5.4 Hz, 1H, 4'-OH), 4.83 (d, J = 4.9 Hz, 1H, 3'-OH), 4.38 (t, J = 5.9 Hz, 1H, 6'-OH), 3.75 (ddt, J = 11.4, 9.7, 4.9 Hz, 1H, H-3'), 3.51 (ddd, J = 11.8, 5.6, 2.3 Hz, 1H, H-6'a), 3.46 (dt, J = 11.5, 5.7 Hz, 1H, H-6'b), 3.37 (ddd, J = 9.8, 5.0, 2.3 Hz, 1H, H-5'), 3.12 (td, J = 9.3, 5.4 Hz, 1H, H-4'), 2.83 – 2.73 (m, 2H, H-6a, H-6b), 2.40 (dd, J = 18.9, 8.6 Hz, 1H, H-16a), 2.34 – 2.29 (m, 1H, H-11a), 2.15 (m, 1H, H-9), 2.07 – 1.99 (m, 2H, H-2'a, H-16b), 1.96 – 1.87 (m, 2H, H-7a, H-15a), 1.76 – 1.68 (m, 1H, H-12a), 1.61 – 1.41 (m, 4H, H-2'b, H-8, H-14, H-15b), 1.41 – 1.27 (m, 3H, H-7b, H-11b, H-12b), 0.80 (s, 3H, H-18).

^{13}C NMR (175 MHz, DMSO-d_6, $(CD_3)_2SO$): δ_c 220.10 (C-17), 154.89 (C-3), 137.75 (C-5), 133.36 (C-10), 126.55 (C-1), 117.05 (C-4), 114.71 (C-2), 96.26 (C-1'), 74.39 (C-5'), 71.70 (C-4'), 68.18 (C-3'), 61.13 (C-6'), 50.03 (C-14), 47.76 (C-13), 43.93 (C-9), 38.31 (C-2'), 38.19 (C-8), 35.81 (C-16), 31.80 (C-12), 29.55 (C-6), 26.47

(C-7), 25.89 (C-11), 21.58 (C-15), 13.95 (C-18).

E1-β-2DG (2c): Chemical Formula: $C_{24}H_{32}O_6$, Molecular Weight: 416.5140

E1-β-2DG (**2c**) displayed $[M + HCOO]^-$ theoretical and experimental peaks at approximately *m*/*z* = 461.2181 and *m*/*z* = 461.2166, respectively.

^{1}H NMR (700 MHz, DMSO-*d*$_6$, $(CD_3)_2SO$): δ 7.13 (d, *J* = 8.6 Hz, 1H, H-1), 6.74 (dd, *J* = 8.6, 2.5 Hz, 1H, H-2), 6.68 (d, *J* = 2.4 Hz, 1H, H-4), 5.12 (dd, *J* = 9.7, 1.7 Hz, 1H, H-1'), 4.92 (t, *J* = 5.0 Hz, 2H, 3'-OH, 4'-OH), 4.48 (t, *J* = 5.8 Hz, 1H, 6'-OH), 3.66 (ddd, *J* = 11.8, 5.5, 2.2 Hz, 1H, H-6'a), 3.51 – 3.40 (m, 2H, H-3', H-6'b), 3.18 (ddd, *J* = 9.6, 5.9, 2.2 Hz, 1H, H-5'), 3.01 (td, *J* = 9.1, 5.1 Hz, 1H, H-4'), 2.77 (m, 2H, H-6a, H-6b), 2.40 (dd, *J* = 18.9, 8.5 Hz, 1H, H-16a), 2.35 – 2.28 (m, 1H), 2.15 (m, 1H, H-9), 2.09 (ddd, *J* = 11.9, 4.9, 1.7 Hz, 1H, H-2'a), 2.03 (dt, *J* = 18.5, 8.9 Hz, 1H, H-16b), 1.96 – 1.87 (m, 2H), 1.72 (dd, *J* = 8.9, 2.6 Hz, 1H, H-12a), 1.57 – 1.42 (m, 4H, H-2'b, H-8, H-14, H-15b), 1.39 – 1.26 (m, 3H, H-7b, H-11b, H-12b), 0.80 (s, 3H, H-18).

^{13}C NMR (175 MHz, DMSO-*d*$_6$, $(CD_3)_2SO$): δ_c 220.09 (C-17), 155.15 (C-3), 137.76 (C-5), 133.36 (C-10), 126.51 (C-1), 116.56 (C-4), 114.01 (C-2), 97.18 (C-1'), 77.70 (C-5'), 71.70 (C-4'), 70.87 (C-3'), 61.32 (C-6'), 50.01 (C-14), 47.76 (C-13), 43.93 (C-9), 39.61 (C-2') 38.25 (C-8), 35.82 (C-16), 31.79 (C-12), 29.61 (C-6), 26.45 (C-7), 25.91 (C-11), 21.58 (C-15), 13.95 (C-18).

E2-β-Glc (3a): Chemical Formula: $C_{24}H_{34}O_7$, Molecular Weight: 434.5290

E2-β-Glc (**3a**) exhibited $[M + HCOO]^-$ theoretical and experimental peaks at approximately *m*/*z* = 479.2287 and *m*/*z* = 479.2291, respectively.

^{1}H NMR (700 MHz, methanol-*d*$_4$, CD_3OD): δ 7.03 (d, *J* = 8.5 Hz, 1H, H-1), 6.51 (dd, *J* = 8.4, 2.7 Hz, 1H, H-2), 6.44 (d, *J* = 2.7 Hz, 1H, H-4), 4.34 (d, *J* = 7.8 Hz, 1H, H-1'), 3.84 (dd, *J* = 11.9, 2.3 Hz, 1H, H-6'a), 3.79 (t, *J* = 8.5 Hz, 1H, H-17), 3.66 (dd,

J = 11.9, 5.6 Hz, 1H, H-6’b), 3.33 (t, J = 9.0 Hz, 1H, H-3’), 3.27 (d, J = 8.9 Hz, 1H, H-4’), 3.24 – 3.20 (m, 1H, H-5’), 3.15 (dd, J = 9.1, 7.8 Hz, 1H, H-2’), 2.78 – 2.67 (m, 2H, H-6a, H-6b), 2.23 (dt, J = 12.6, 3.9 Hz, 1H), 2.10 – 2.00 (m, 3H, H-9, H-12a, H-16a), 1.81 (ddt, J = 12.5, 5.7, 2.5 Hz, 1H, H-7a), 1.69 – 1.59 (m, 2H, H-15a, H-16b), 1.44 – 1.20 (m, 5H, H-7b, H-8, H-11b, H-12b, H-15b), 1.15 (ddd, J = 12.3, 10.8, 7.1 Hz, 1H, H-14), 0.84 (s, 3H, H-18).

^{13}C NMR (175 MHz, methanol-d_4, CD_3OD): δc 154.33 (C-3), 137.24 (C-5), 131.06 (C-10), 125.69 (C-1), 114.54 (C-4), 112.22 (C-2), 103.18 (C-1’), 88.29 (C-17), 76.63 (C-3’), 76.31 (C-5’), 73.89 (C-2’), 70.16 (C-4’), 61.29 (C-6’), 49.62 (C-14), 43.74 (C-9), 43.04 (C-3), 38.76 (C-8), 37.23 (C-12), 29.18 (C-6), 28.36 (C-16), 26.93 (C-7), 26.09 (C-11), 22.47 (C-15), 10.61 (C-18).

E2-α-2DG (**3b**): Chemical Formula: $C_{24}H_{34}O_6$, Molecular Weight: 418.5300

E2-α-2DG (**3b**) exhibited $[M + HCOO]^-$ theoretical and experimental peaks at approximately m/z = 463.2337 and m/z = 463.2327, respectively.

^{1}H NMR (700 MHz, DMSO-d_6, $(CD_3)_2SO$): δ 7.12 (d, J = 8.6 Hz, 1H, H-1), 6.76 (dd, J = 8.6, 2.7 Hz, 1H, H-2), 6.71 (d, J = 2.7 Hz, 1H, H-4), 5.50 (d, J = 2.6 Hz, 1H, H-1’), 4.88 (d, J = 5.4 Hz, 1H, 4’-OH), 4.83 (d, J = 4.9 Hz, 1H, 3’-OH), 4.45 (d, J = 4.8 Hz, 1H, 17-OH), 4.38 (t, J = 5.9 Hz, 1H, 6’-OH), 3.74 (ddd, J = 15.2, 9.1, 4.8 Hz, 1H, H-3’), 3.53 – 3.42 (m, 3H, H-6’a, H-6’b, H-17), 3.37 (ddd, J = 10.0, 4.9, 2.3 Hz, 1H, H-5’), 3.12 (td, J = 9.0, 5.4 Hz, 1H, H-4’), 2.72 (dt, J = 7.1, 4.6 Hz, 2H, H-6a, H-6b), 2.25 – 2.20 (m, 1H, H-11a), 2.10 – 2.01 (m, 2H, H-2’a, H-9), 1.88 – 1.78 (m, 2H, H-12a, H-16a), 1.75 (ddd, J = 9.7, 5.3, 2.7 Hz, 1H, H-7a), 1.59 – 1.51 (m, 2H, H-2’b, H-15a), 1.37 – 1.17 (m, 5H, H-7b, H-8, H-11b, H-15b, H-16b), 1.14 (td, J = 13.0, 4.2 Hz, 1H, H-12b), 1.10 – 1.04 (m, 1H, H-14), 0.63 (s, 3H, H-18).

^{13}C NMR (175 MHz, DMSO-d_6, $(CD_3)_2SO$): δc 154.79 (C-3), 137.77 (C-5), 133.88

(C-10), 126.50 (C-1), 117.06 (C-4), 114.60 (C-2), 96.29 (C-1'), 80.48 (C-17), 74.36 (C-5'), 71.70 (C-4'), 68.18 (C-3'), 61.12(C-6'), 49.98 (C-14), 44.02 (C-9), 43.24 (C-13), 38.93 (C-8), 38.32 (C-2'), 37.02 (C-12), 30.33 (C-16), 29.63 (C-6), 27.27 (C-7), 26.42 (C-11), 23.21 (C-15), 11.68 (C-18).

E2-β-2DG (3c): Chemical Formula: $C_{24}H_{34}O_6$, Molecular Weight: 418.5300

E2-β-2DG (**3c**) exhibited $[M + HCOO]^-$ theoretical and experimental peaks at approximately *m*/*z* = 463.2337 and *m*/*z* = 463.2336, respectively.

^{1}H NMR (700 MHz, methanol-d_4, CD_3OD): δ 7.04 (d, *J* = 8.5 Hz, 1H, H-1), 6.51 (dd, *J* = 8.5, 2.7 Hz, 1H, H-2), 6.45 (d, *J* = 2.6 Hz, 1H, H-4), 4.62 (dd, *J* = 9.7, 1.9 Hz, 1H, H-1'), 3.85 (dd, *J* = 11.7, 1.8 Hz, 1H, H-6'a), 3.79 (t, *J* = 8.6 Hz, 1H, H-17), 3.67 (dd, *J* = 11.8, 5.1 Hz, 1H, H-6'b), 3.55 – 3.49 (m, 1H, H-3'), 3.19 – 3.12 (m, 2H, H-4', H-5'), 2.81 – 2.69 (m, 2H, H-6a, H-6b), 2.30 – 2.23 (m, 1H, H-11a), 2.15 – 2.09 (m, 2H, H-2'a, H-13), 2.09 – 2.03 (m, 1H, H-16a), 1.96 – 1.91 (m, 1H, H-12a), 1.84 (ddt, *J* = 12.3, 5.7, 2.4 Hz, 1H, H-7a), 1.69 – 1.58 (m, 2H, H-15a, H-16b), 1.50 – 1.40 (m, 3H, H-2'b, H-11b, H-12b), 1.39 – 1.27 (m, 3H, H-8, H-7a, H-15b), 1.19 (m, 1H, H-14), 0.78 (s, 3H, H-18).

^{13}C NMR (175 MHz, methanol-d_4, CD_3OD): δ_c 155.92 (C-3), 138.79 (C-5), 132.53 (C-10), 127.20 (C-1), 116.05 (C-4), 113.74 (C-2), 101.54 (C-1'), 89.71 (C-17), 78.07 (C-5'), 73.14 (C-4'), 72.64 (C-3'), 63.02 (C-6'), 51.12 (C-14), 45.31 (C-9), 44.48 (C-13), 40.67 (C-2'), 40.35 (C-8), 38.92 (C-12), 30.70 (C-6), 29.90 (C-16), 28.46 (C-7), 27.64 (C-11), 24.01 (C-15), 12.27 (C-18).

In vitro Aromatase Inhibitory Assays

The aromatase inhibitory activity of the compounds was evaluated using a CYP19A1/7-methoxy-4-trifluoromethyl coumarin (MFC) assay.[2] In this assay, MFC

served as the substrate, while human recombinant CYP19A1 enzyme was used as the enzymatic source. The conversion of MFC to its fluorescent metabolite, 7-hydroxy trifluoromethyl coumarin (HFC), was facilitated by a reduced nicotinamide adenine dinucleotide phosphate (NADPH)-generating system, with a reduction in fluorescence intensity indicating aromatase inhibition. Each reaction (100 μL) contained serially diluted compounds, an NADPH-cofactor mixture (16.25 μM $NADP^+$, 825.14 μM $MgCl_2$, 825.14 μM glucose-6-phosphate (G6P), and 0.4 U/mL G6PDH), and incubated at 37 °C for 10 minutes before adding recombinant human aromatase, 50 μM MFC, and 20 mM Tris-HCl buffer (pH 7.4). After a 15-minute incubation, fluorescence was measured at 409 nm (excitation) and 530 nm (emission) using a Tecan Infinite F200 Fluorescence Microplate Reader. Inhibition was calculated relative to control wells, and IC_{50} values were determined using nonlinear regression (Origin 2016), assuming 100% enzyme activity.

Cell Cytotoxicity Assays of *trans*-Androsterone, Estrone, and Estradiol Derivatives

MCF-7, MDA-MB-231 (breast cancer), and HEK293 (normal) cell lines were obtained from ATCC. MDA-MB-231 and HEK293 cells were cultured in DMEM supplemented with 10% FBS, while MCF-7 cells were maintained in RPMI-1640 with 10% FBS. All cells were incubated at 37°C in a 5% CO_2 atmosphere. The cell cytotoxicity assay was performed as previous described. Briefly, cells at 80% confluency were trypsinized, seeded in 96-well plates (2.5×10^3 cells/well), and incubated overnight. The following day, the medium was replaced with 1% FBS, and cells were treated with serially diluted sterols (*t*AND, E1, E2) or tested compounds (**1a**–**3c**, **4**) for 48 hours. After treatment, cells were stained with 0.2% crystal violet, washed, dried, and solubilized in 1% SDS. Absorbance was measured at 595 nm using a

microplate reader. Experiments were conducted in triplicate, and IC_{50} values were determined using log-linear regression analysis.

Molecular Docking

Ligand-based molecular docking was performed using iGEMDOCK to identify potential aromatase inhibitor-binding sites.[3] The 3D structure of CYP19A1 (PDB ID: 3S79) was retrieved from the RCSB Protein Data Bank (PDB) (https://www.rcsb.org/structure/3S79).[4] Ligand structures were drawn using ChemDraw 12, converted to SDF files via ChemSketch, and prepared using Open Babel. Docking poses were selected based on the lowest binding free energy, and structural analysis was conducted using PyMOL2 (Accelrys Software Inc., San Diego, CA, USA).

References

1. H. F. Liu, S. C. Chou, S. C. Huang, T. Y. Huang, P. Y. Hsiao, F. P. Chou and T. K. Wu, *Chem. Biol. Drug. Des.* 2024**, 104**, e14624.
2. S. Ozcan-Sezer, E. Ince, A. Akdemir, Ö. Ö. Ceylan, S. Suzen and H. Gurer-Orhan, *Xenobiotica* 2019**, 49**, 549-556.
3. K. C. Hsu, Y. F. Chen, S. R. Lin and J. M. Yang, *BMC Bioinformatics* 2011**, 12**, S33.
4. D. Ghosh, J. Lo, D. Morton, D. Valette, J. Xi and J. Griswold, J. et. al., *J. Med. Chem.* 2012, **55**, 8464-8476.

Fig. S1 High–resolution UPLC/ESI MS and NMR of *t*AND-α-Glc (**1a**). (A) ^{1}H NMR, (B) ^{13}C NMR, (C) C-H coupling constant, (D) DEPT, (E) ^{1}H-^{1}H COSY, (F) HSQC, and (G) HMBC.

(A) ^{1}H NMR

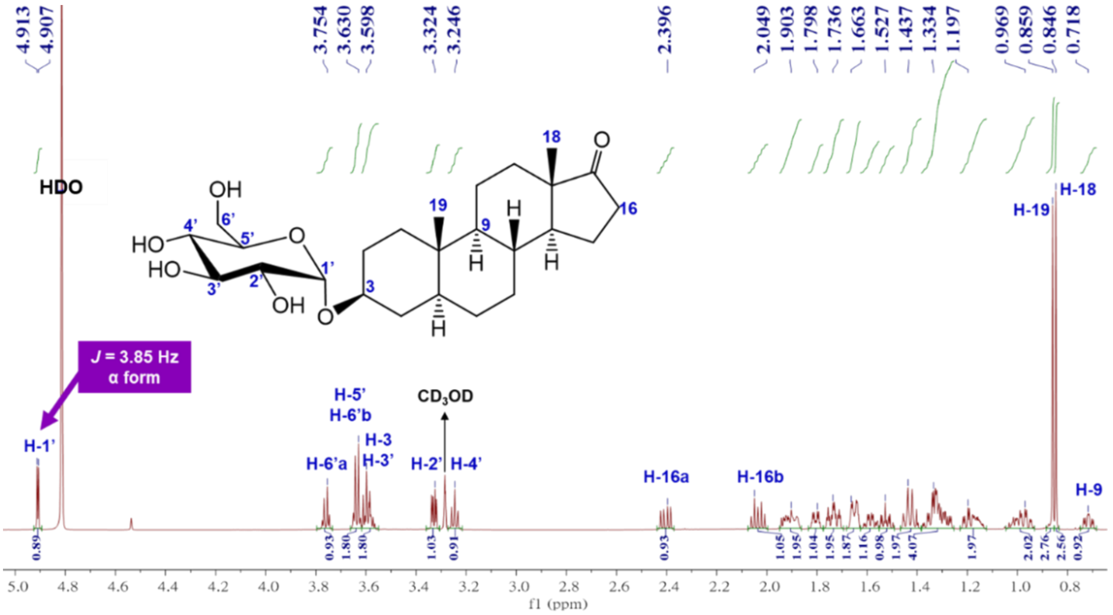

(B) ^{13}C NMR

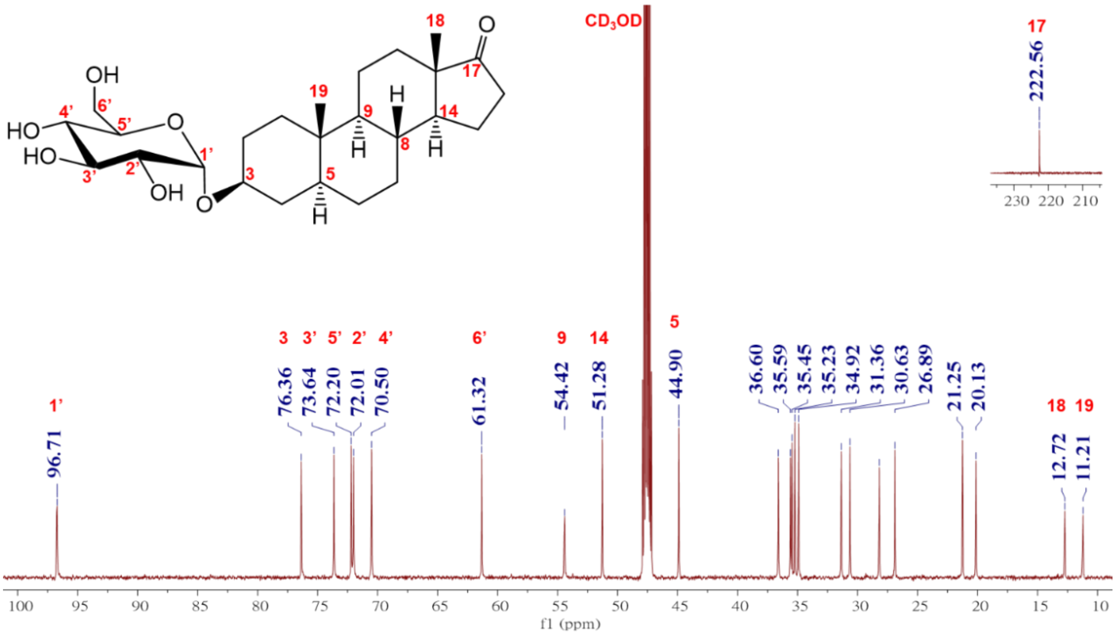

(C) C-H coupling constant

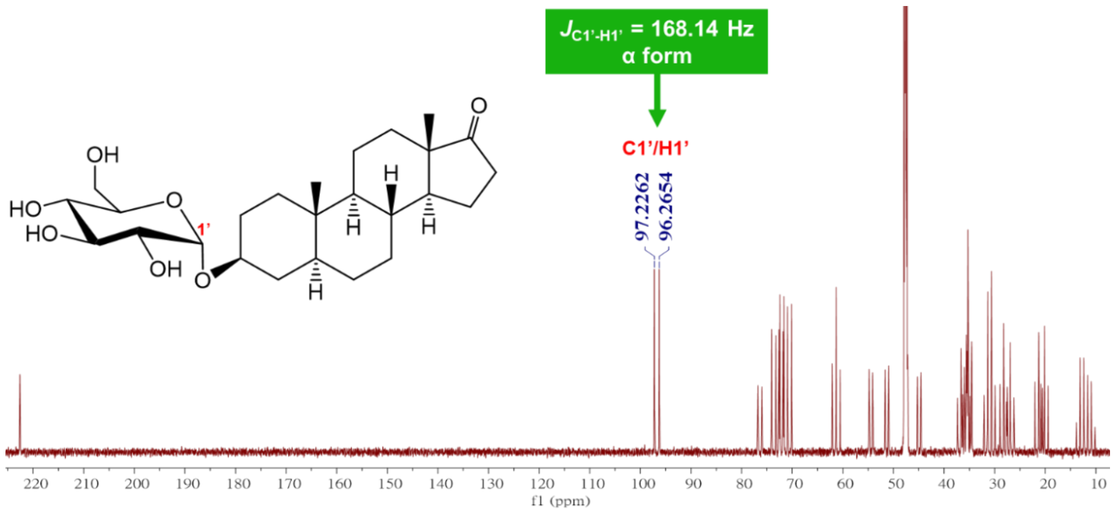

(D) DEPT

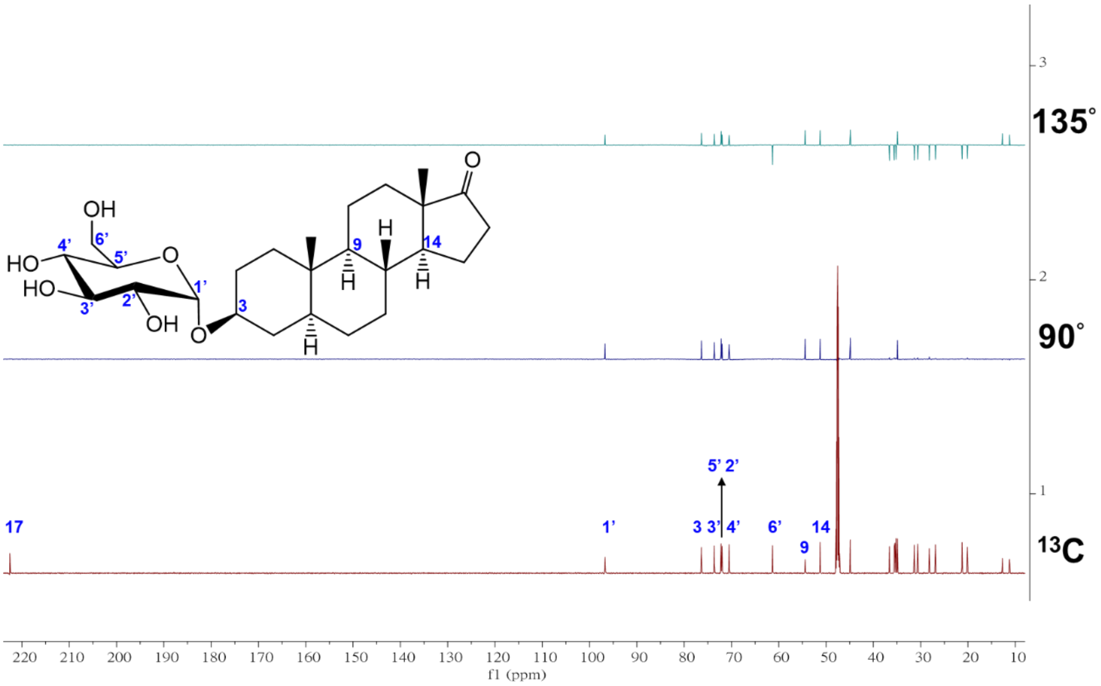

(E) ^{1}H-^{1}H COSY

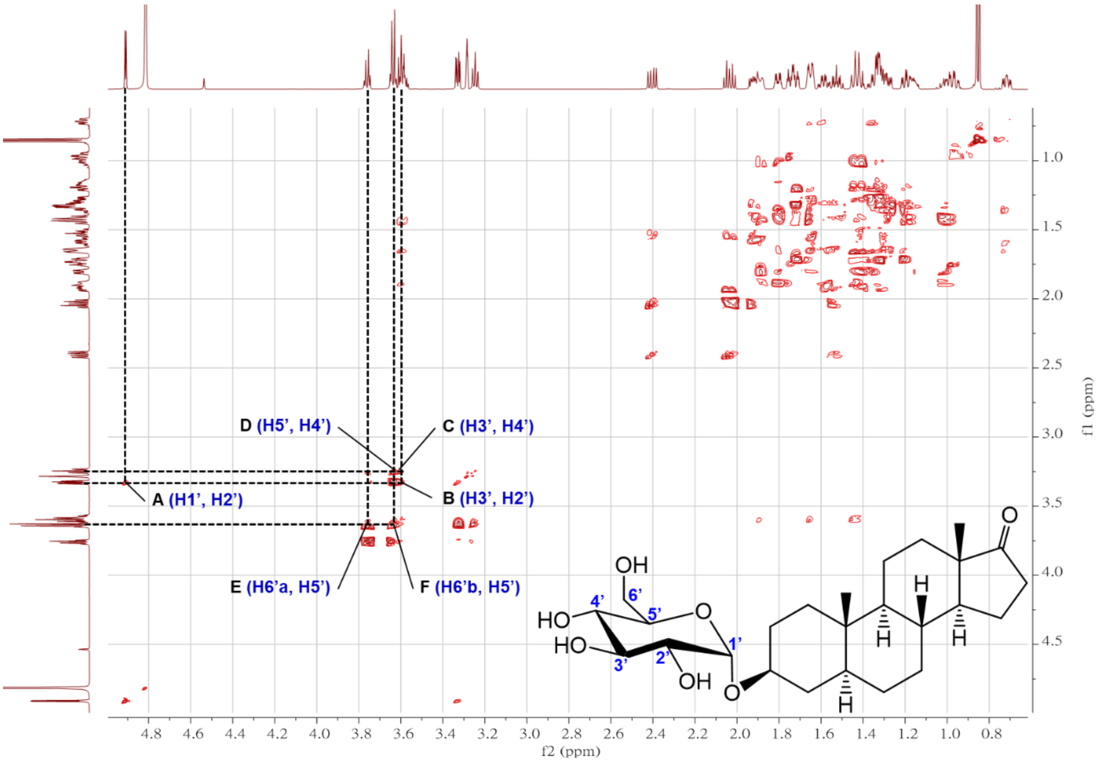
D (H5', H4')
C (H3', H4')
A (H1', H2')
B (H3', H2')
E (H6'a, H5')
F (H6'b, H5')
f1 (ppm)
f2 (ppm)

(F) HSQC

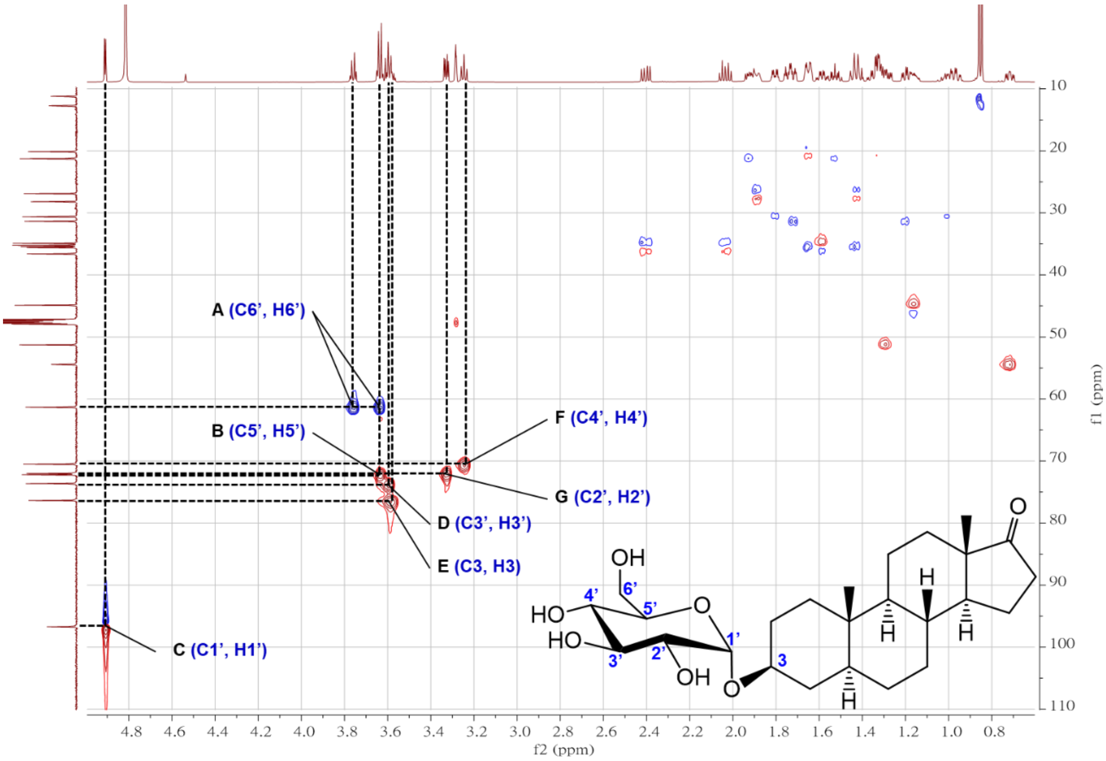
A (C6', H6')
B (C5', H5')
F (C4', H4')
G (C2', H2')
D (C3', H3')
E (C3, H3)
C (C1', H1')
f1 (ppm)
f2 (ppm)

(G) HMBC

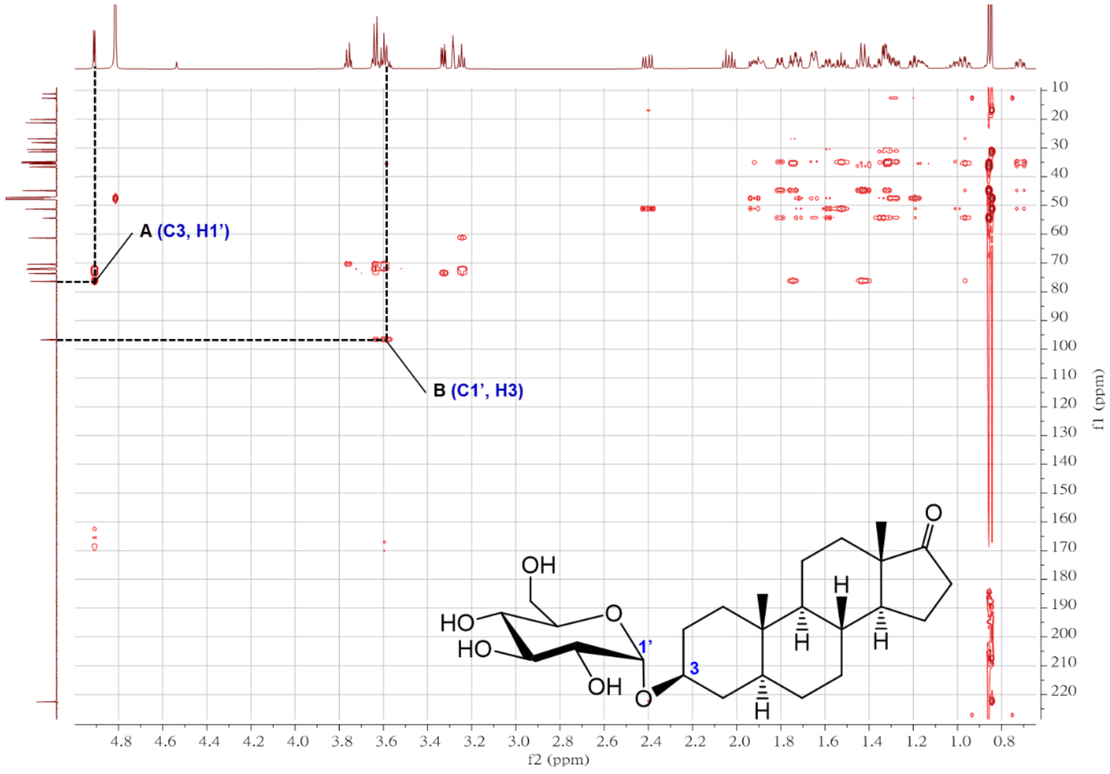
A (C3, H1')
B (C1', H3)
f1 (ppm)
f2 (ppm)
OH
HO
HO
OH
O
O
1'
3
H
H
H
H
O

Fig. S2 High–resolution UPLC/ESI MS and NMR of *t*AND-β-Glc (**1b**). (A) ^{1}H NMR, (B) ^{13}C NMR, (C) C-H coupling constant, (D) DEPT, (E) ^{1}H-^{1}H COSY, (F) HSQC, and (G) HMBC.

(A) ^{1}H NMR

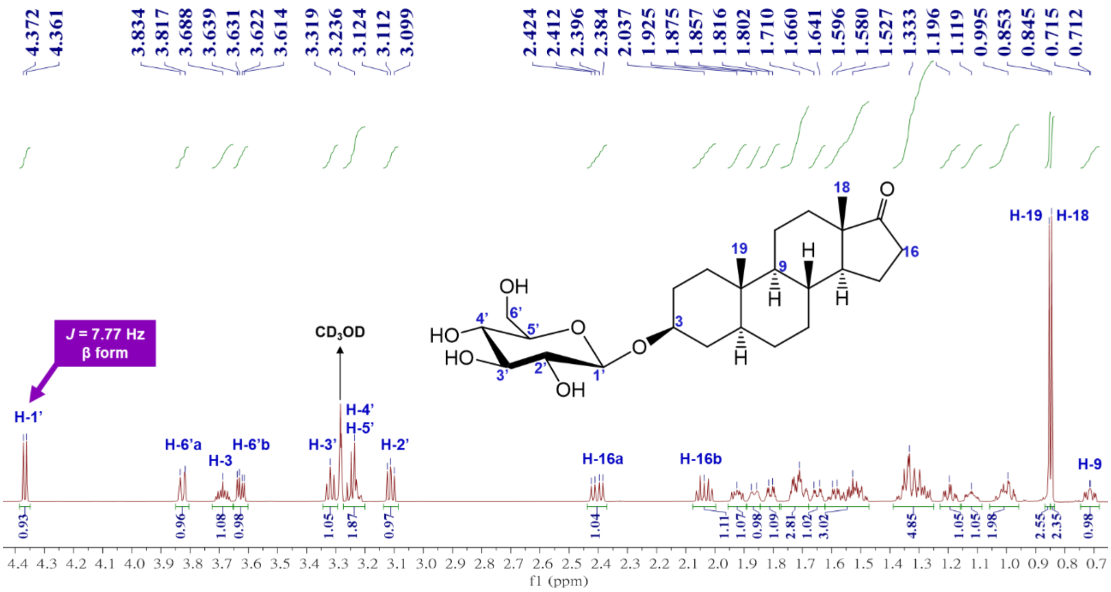

(B) ^{13}C NMR

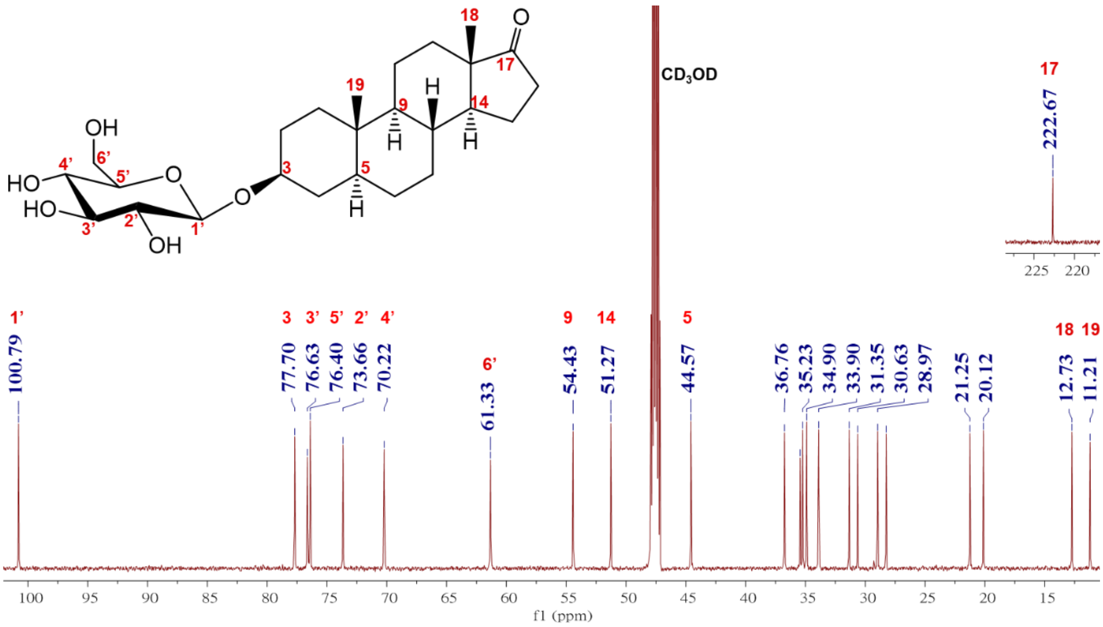

(C) C-H coupling constant

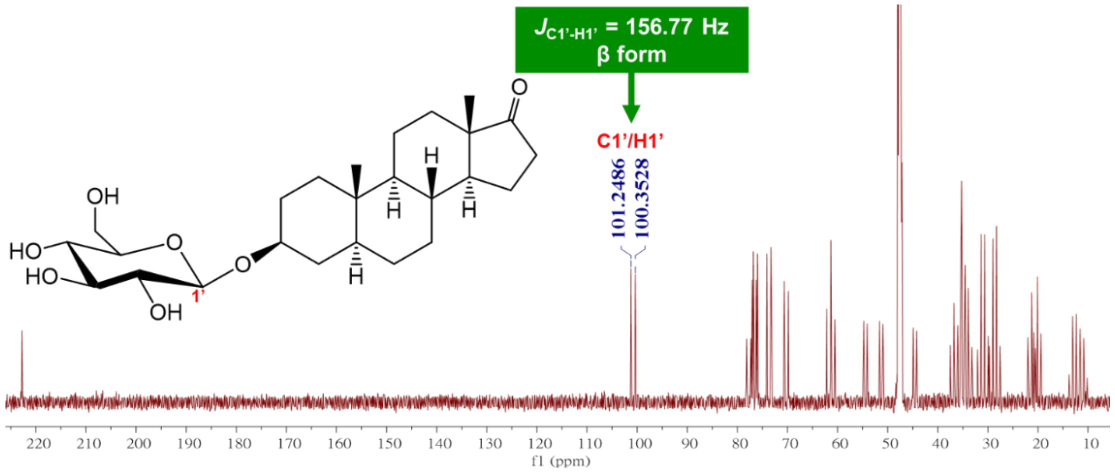

(D) DEPT

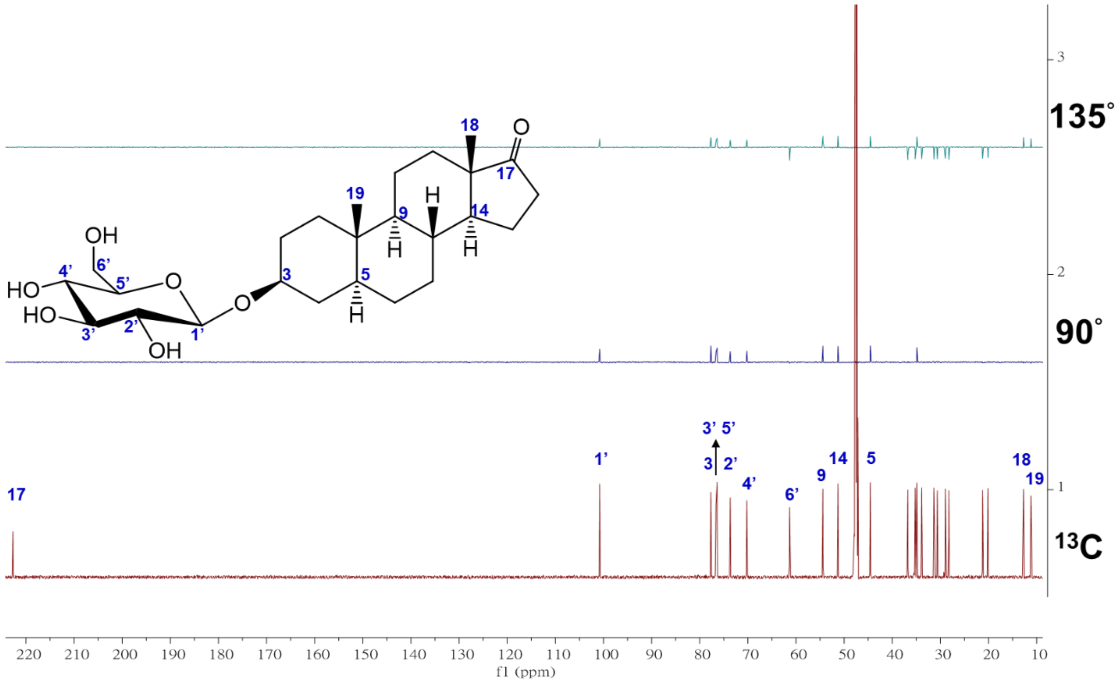

(E) ^{1}H-^{1}H COSY

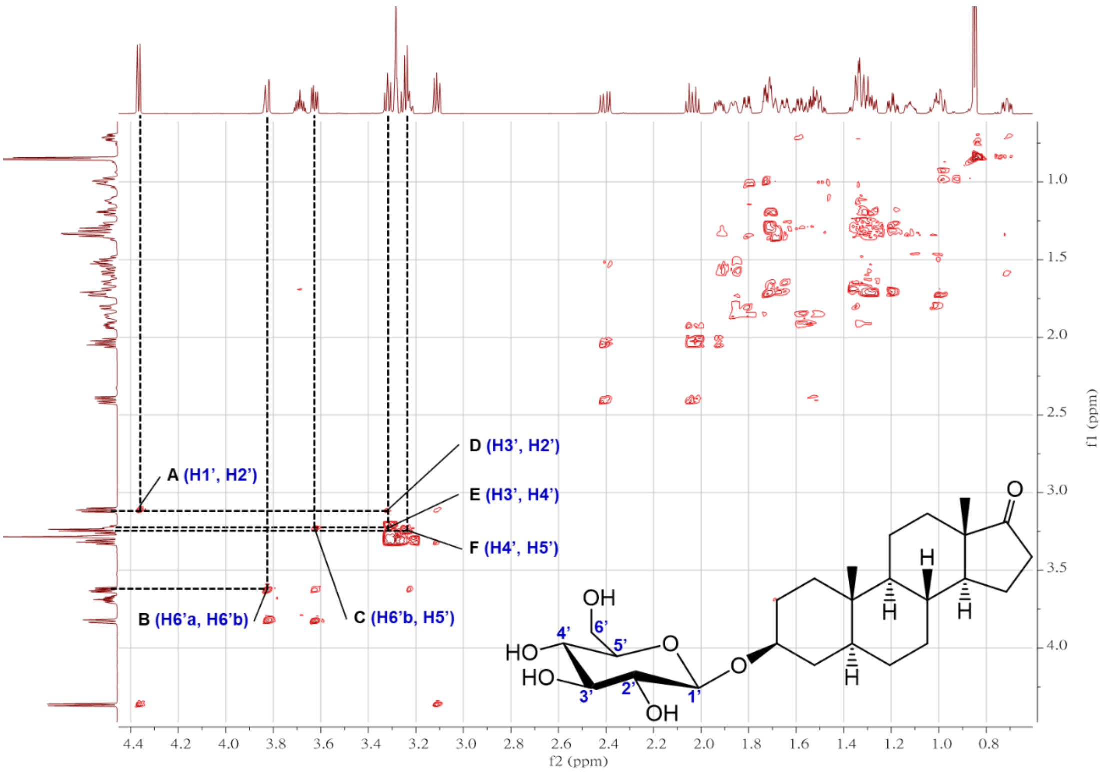

A (H1', H2')
B (H6'a, H6'b)
C (H6'b, H5')
D (H3', H2')
E (H3', H4')
F (H4', H5')
f1 (ppm)
f2 (ppm)

(F) HSQC

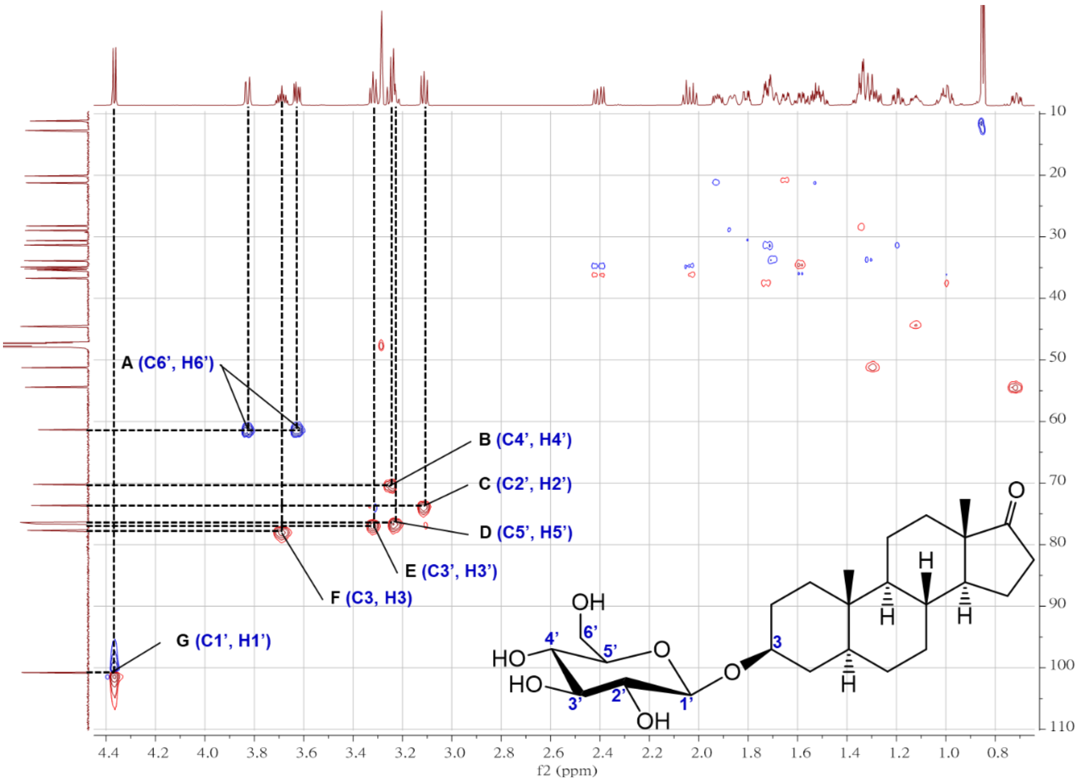

A (C6', H6')
B (C4', H4')
C (C2', H2')
D (C5', H5')
E (C3', H3')
F (C3, H3)
G (C1', H1')
f1 (ppm)
f2 (ppm)

(G) HMBC

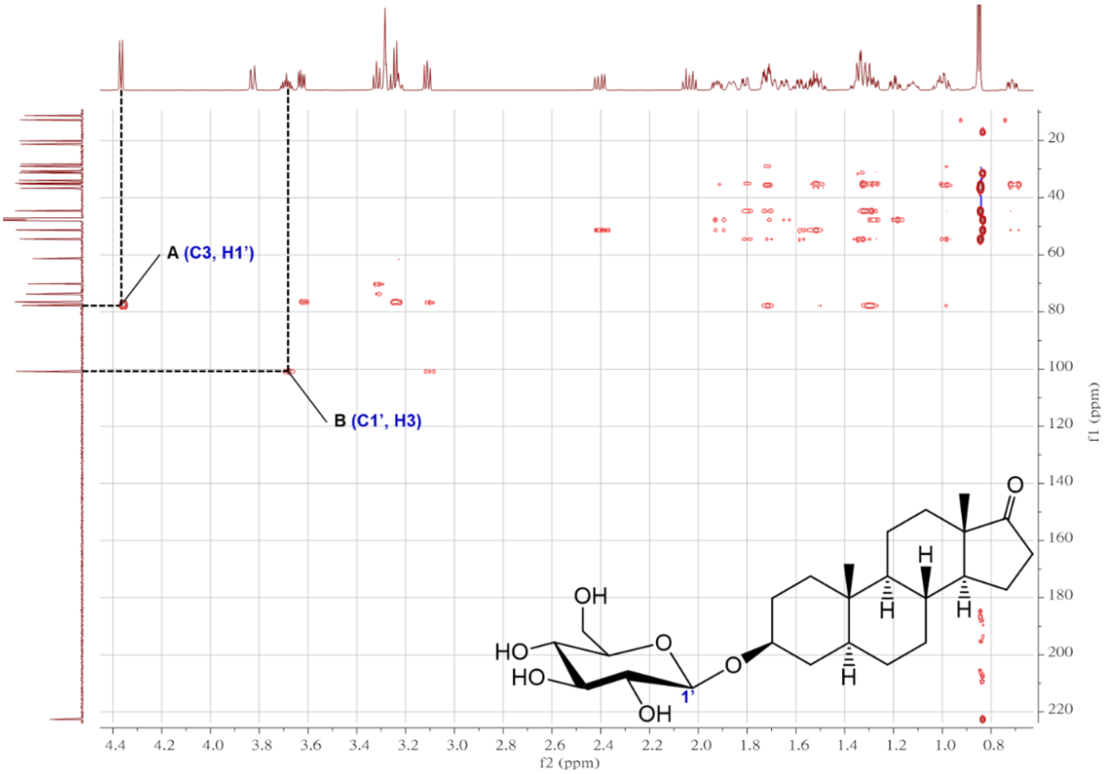
A (C3, H1')
B (C1', H3)
f1 (ppm)
f2 (ppm)
1'

Fig. S3 High–resolution UPLC/ESI MS and NMR of *t*AND-α-2DG (**1c**). (A) ^{1}H NMR, (B) ^{13}C NMR, (C) DEPT, (D) ^{1}H-^{1}H COSY, (E) HSQC, and (F) HMBC.

(A) ^{1}H NMR

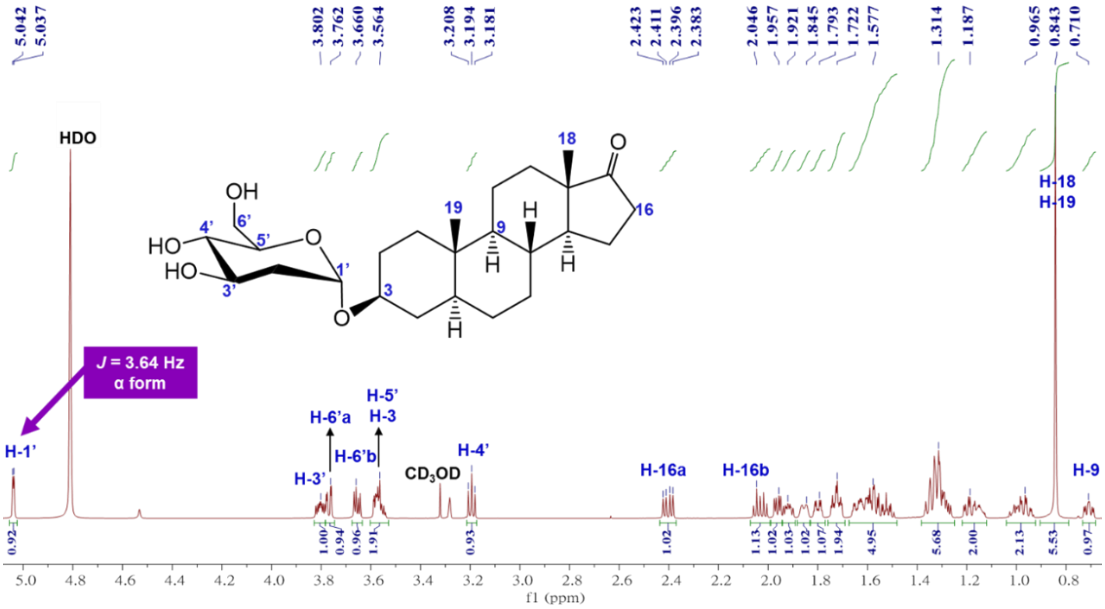

(B) ^{13}C NMR

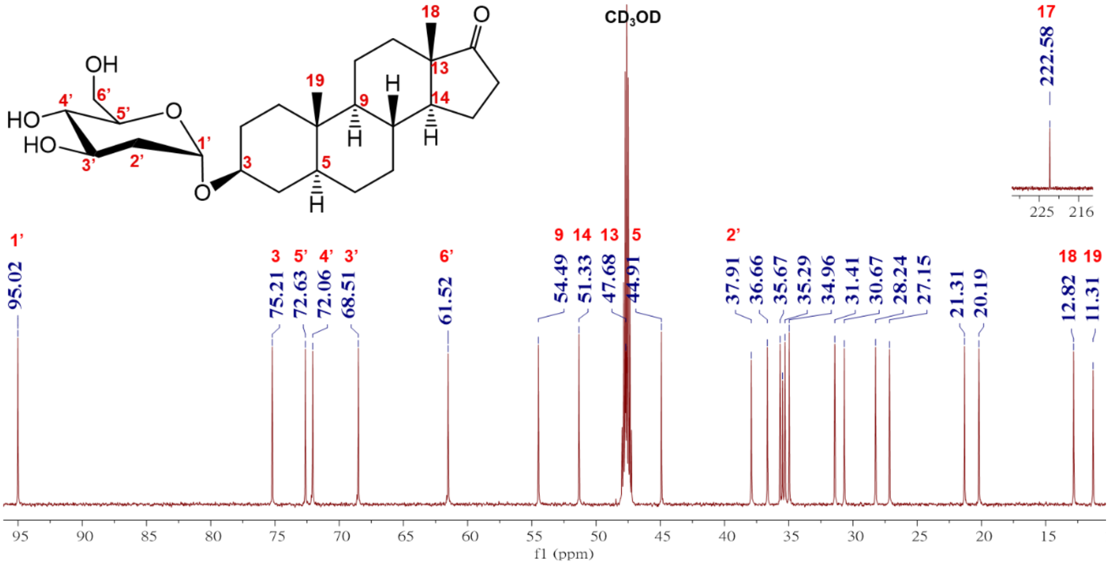

(C) DEPT

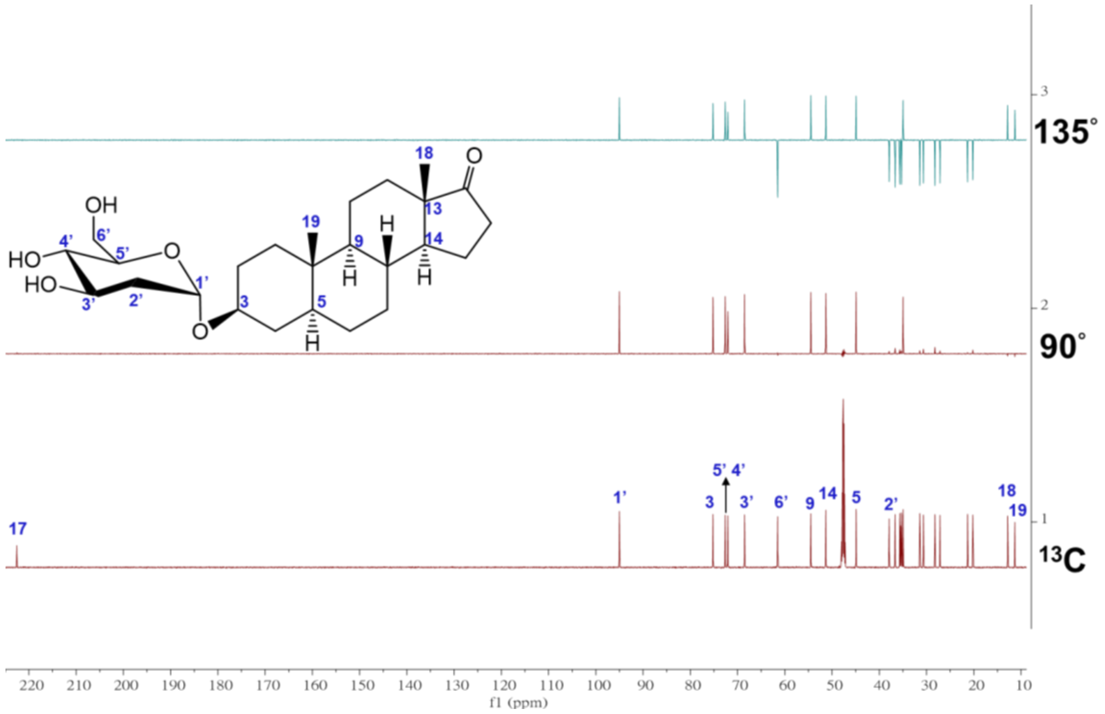

(D) ^{1}H-^{1}H COSY

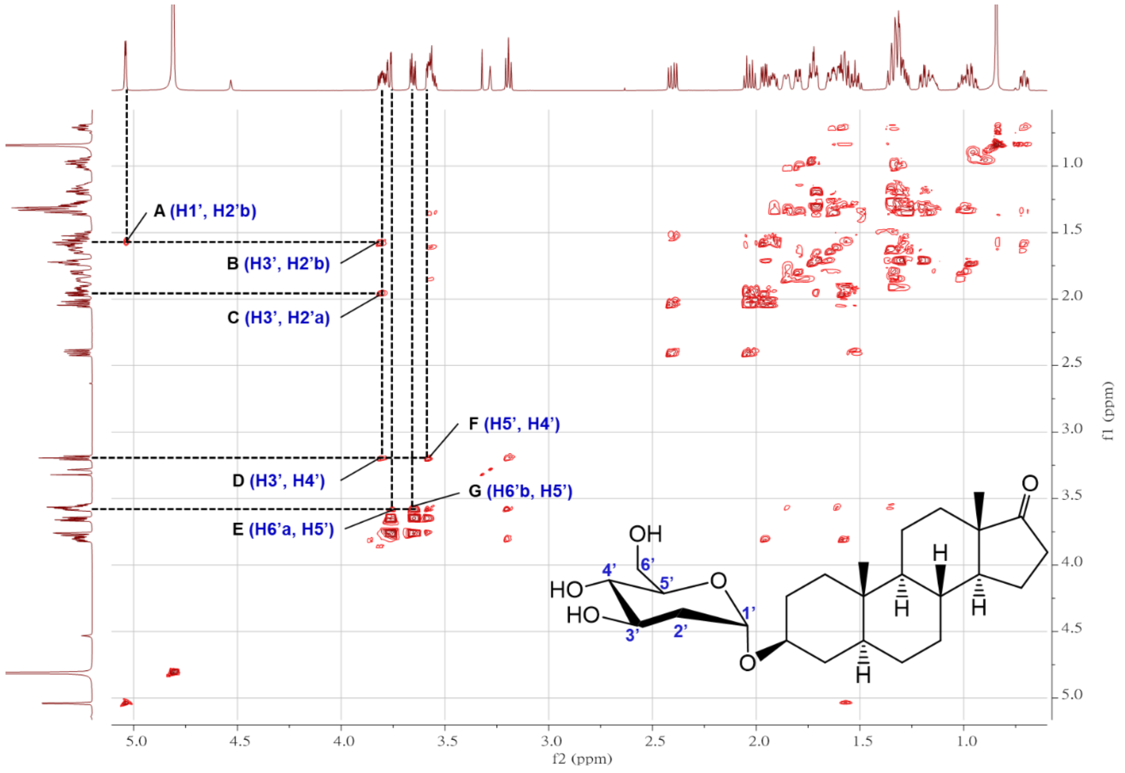

(F) HSQC

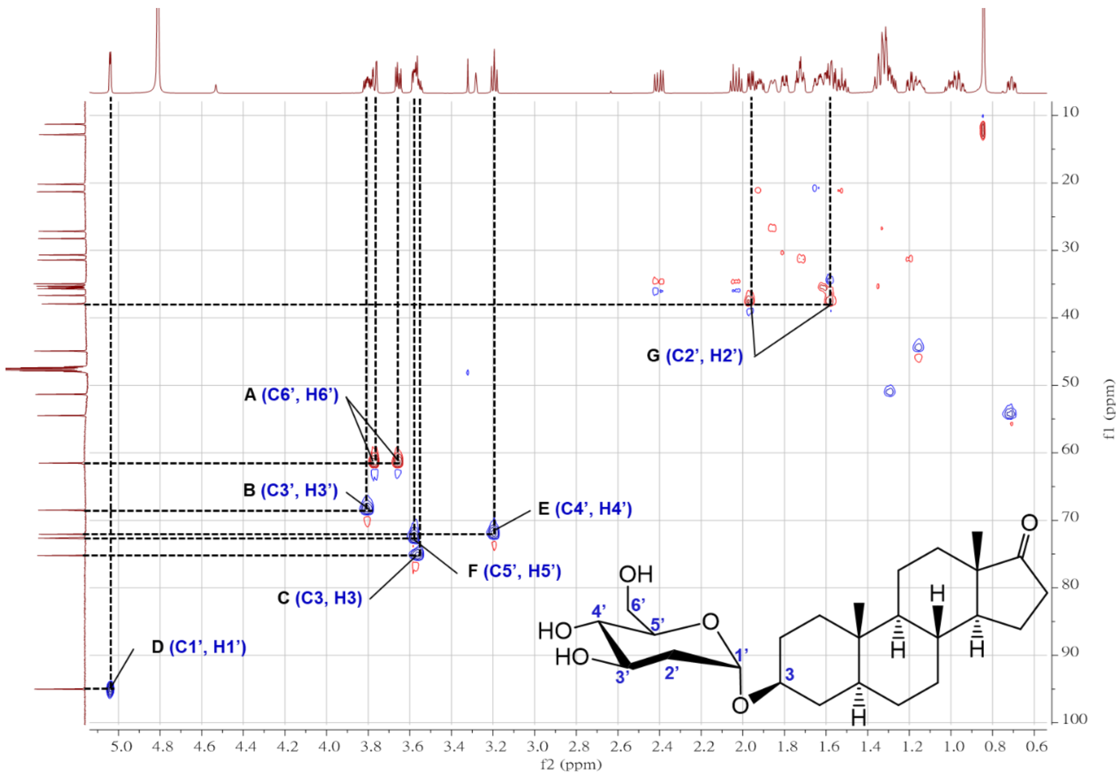
A (C6', H6')
B (C3', H3')
C (C3, H3)
D (C1', H1')
E (C4', H4')
F (C5', H5')
G (C2', H2')
f2 (ppm)
f1 (ppm)

(G) HMBC

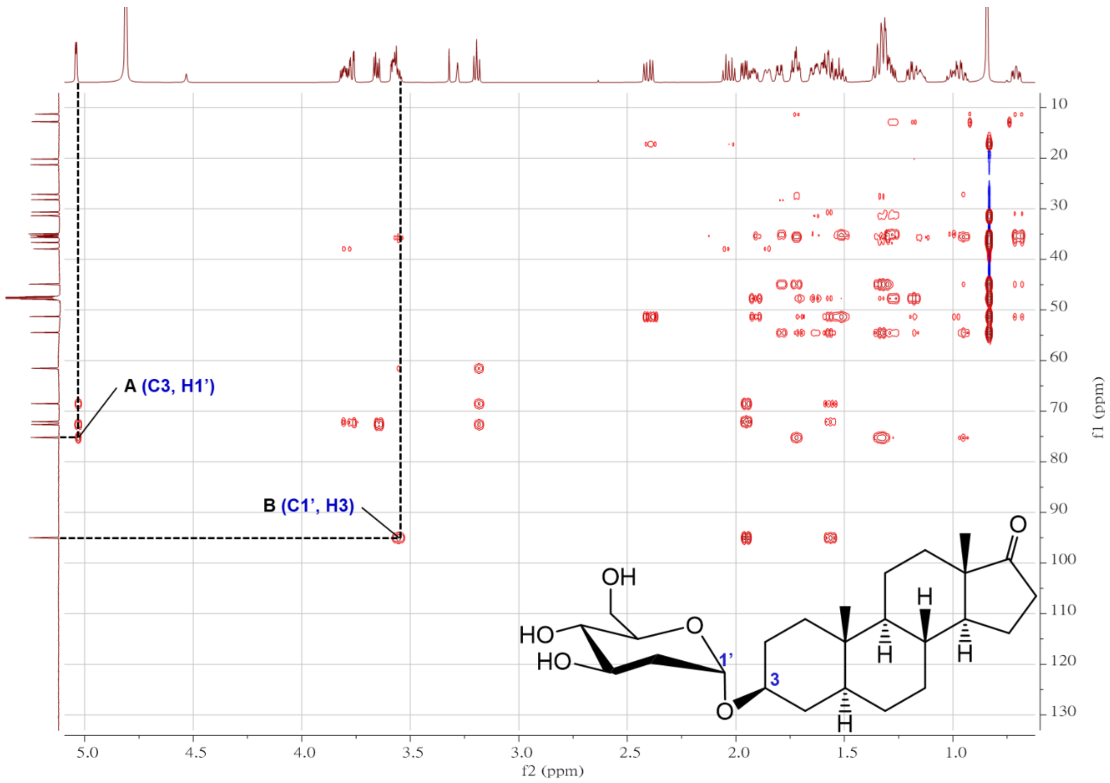
A (C3, H1')
B (C1', H3)
f2 (ppm)
f1 (ppm)

Fig. S4 High–resolution UPLC/ESI MS and NMR of *t*AND-β-2DG (**1d**). (A) ^{1}H NMR, (B) ^{13}C NMR, (C) DEPT, (D) ^{1}H-^{1}H COSY, (E) HSQC, and (F) HMBC.

(A) ^{1}H NMR

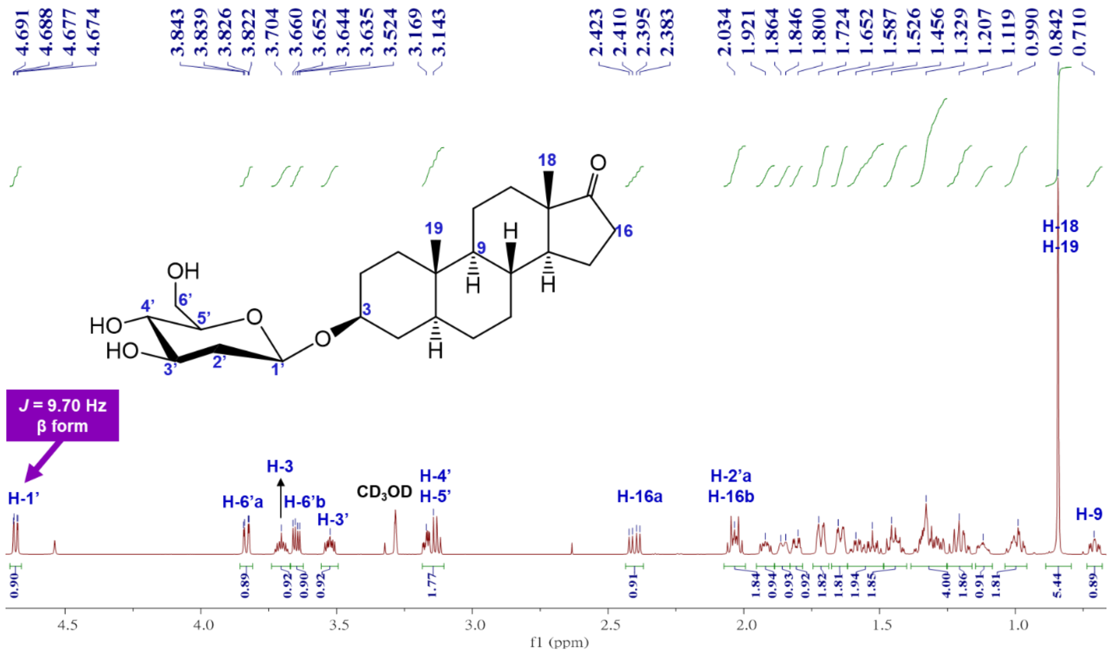

(B) ^{13}C NMR

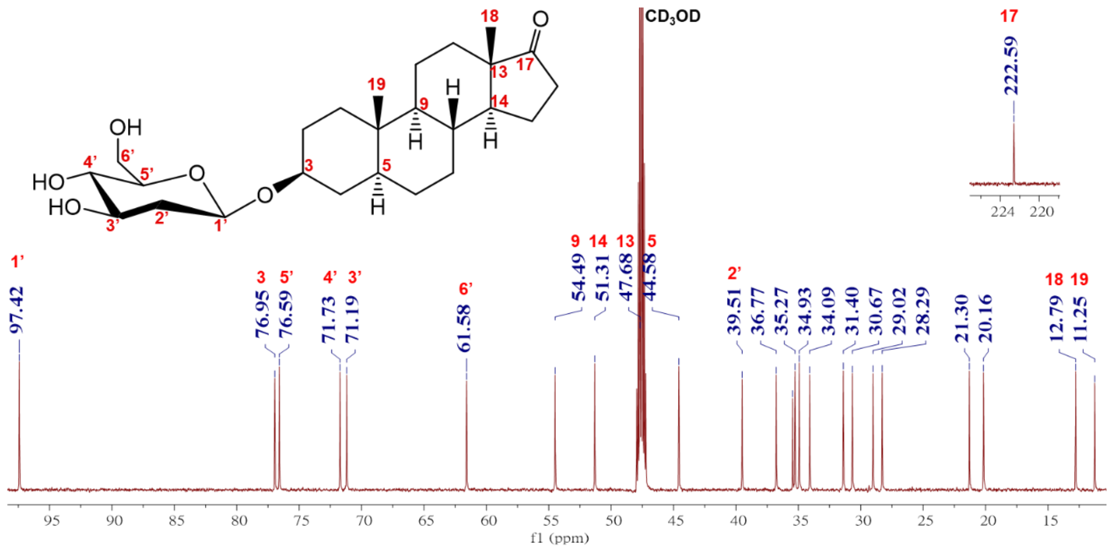

(C) DEPT

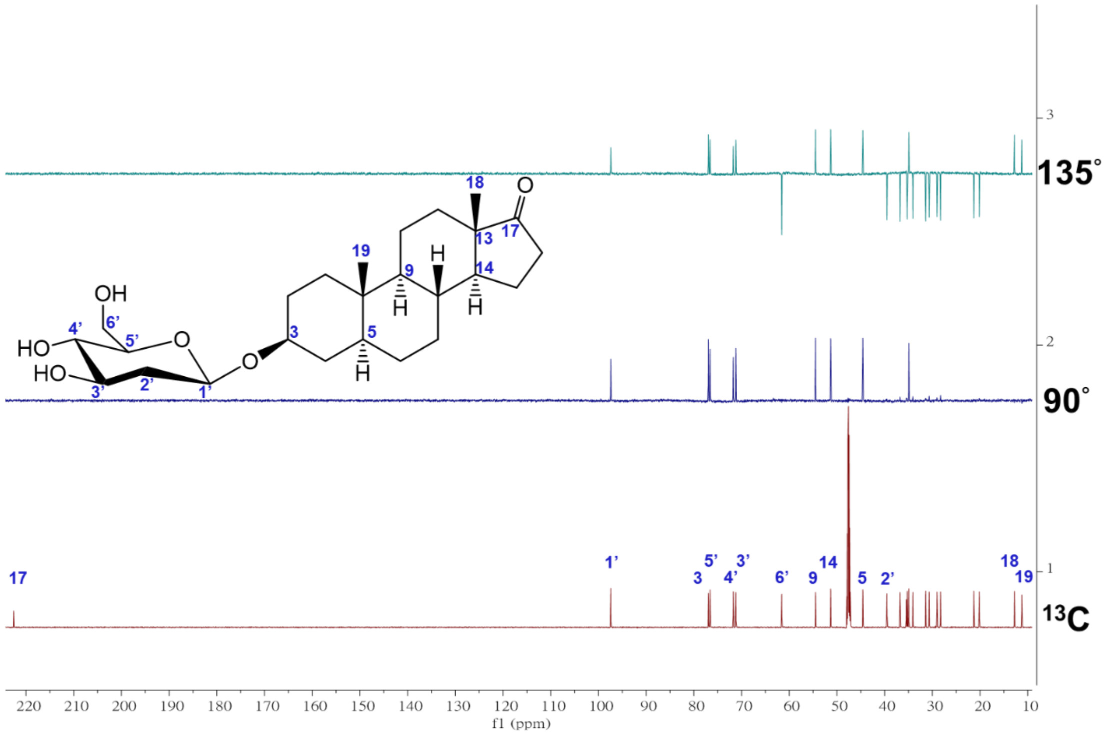

(D) ^{1}H-^{1}H COSY

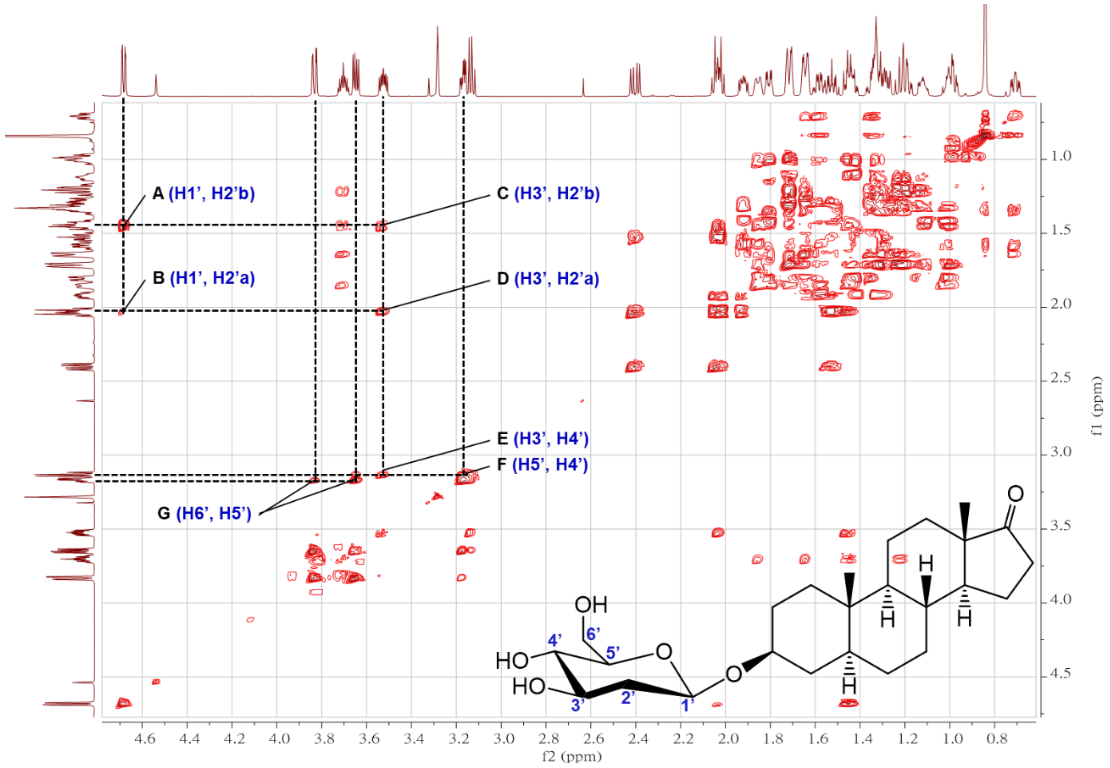

(E) HSQC

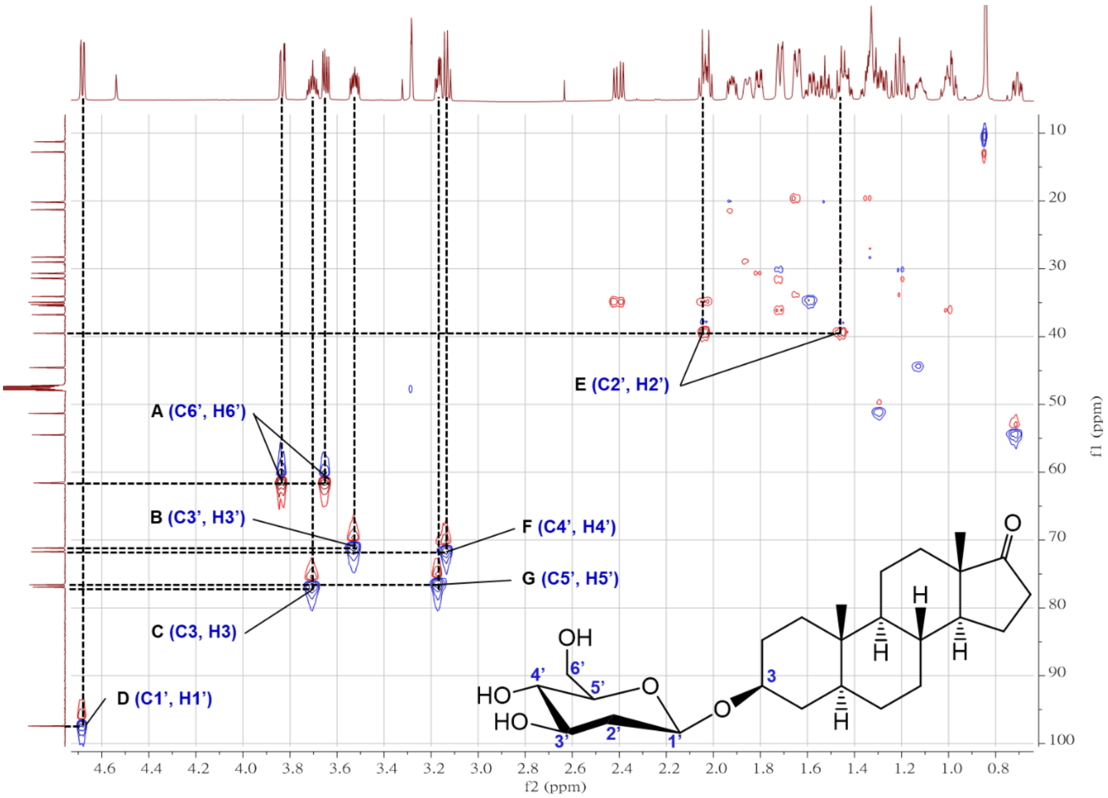

A (C6', H6')
B (C3', H3')
C (C3, H3)
D (C1', H1')
E (C2', H2')
F (C4', H4')
G (C5', H5')
f1 (ppm)
f2 (ppm)

(F) HMBC

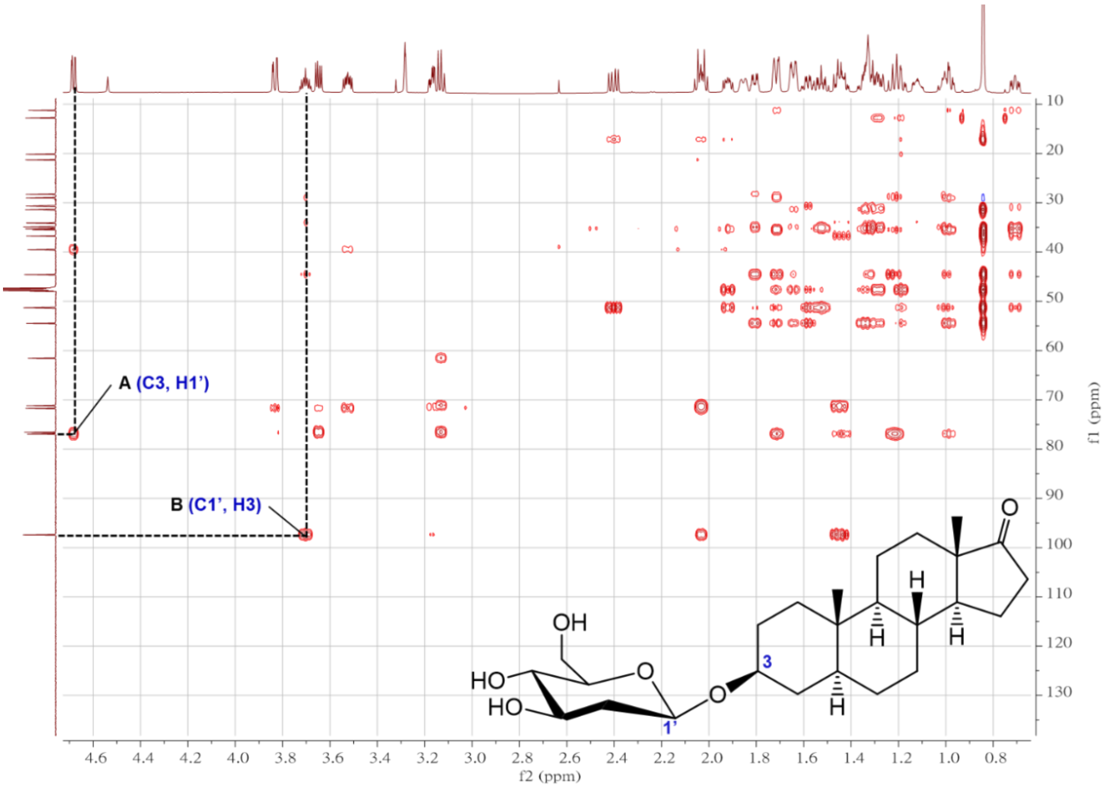

A (C3, H1')
B (C1', H3)
f1 (ppm)
f2 (ppm)

Fig. S5 High–resolution UPLC/ESI MS and NMR of E1-β-Glc (**2a**). (A) ^{1}H NMR, (B) ^{13}C NMR, (C) DEPT, (D) ^{1}H-^{1}H COSY, (E) HSQC, and (F) HMBC.

(A) ^{1}H NMR

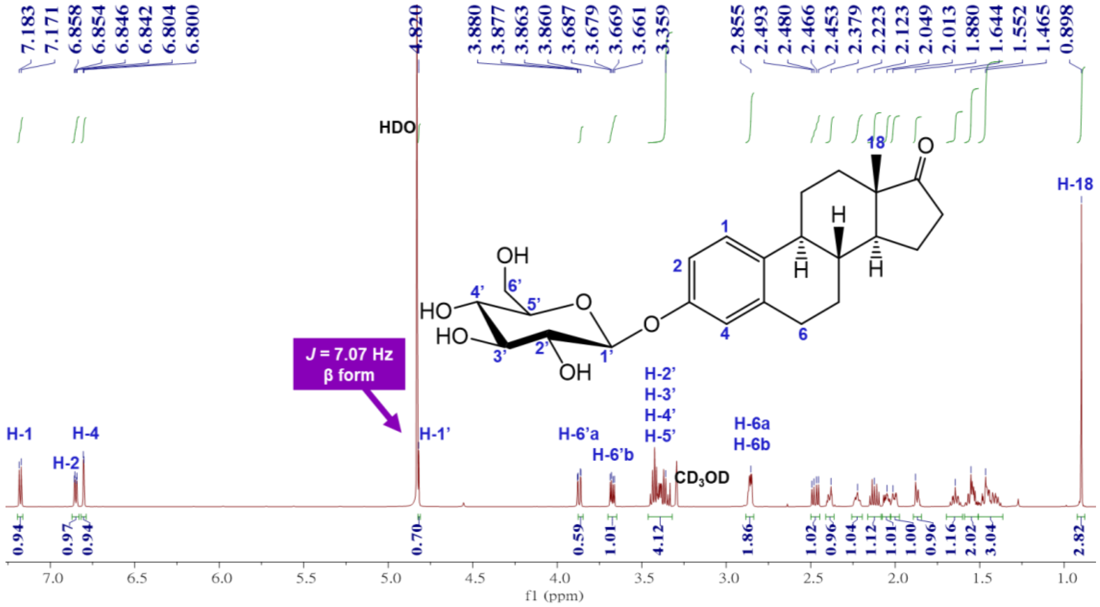

(B) ^{13}C NMR

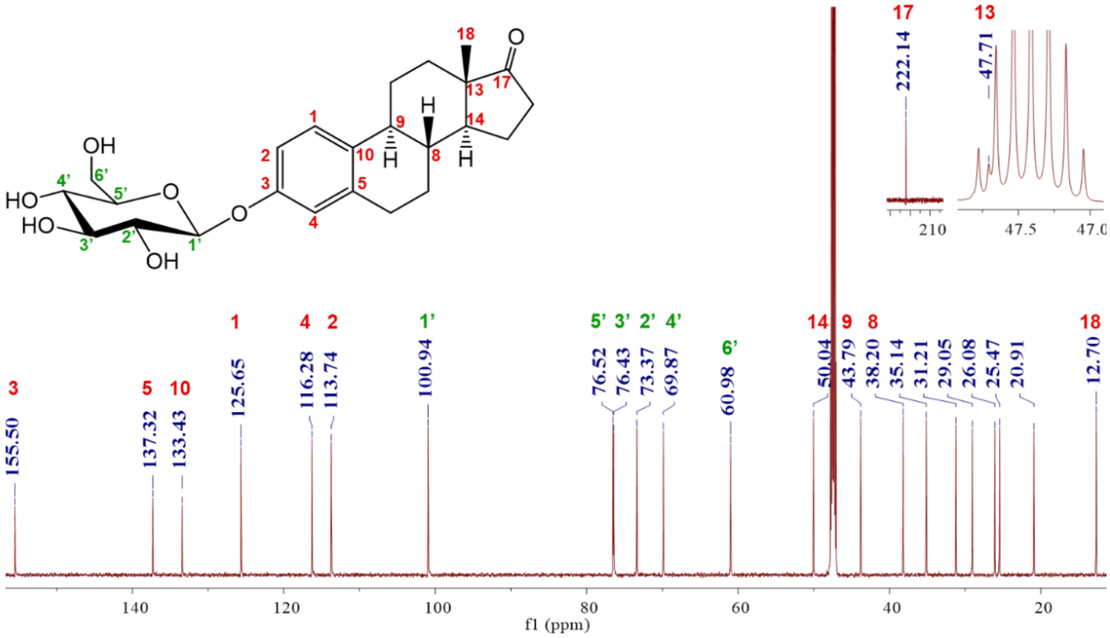

(C) DEPT

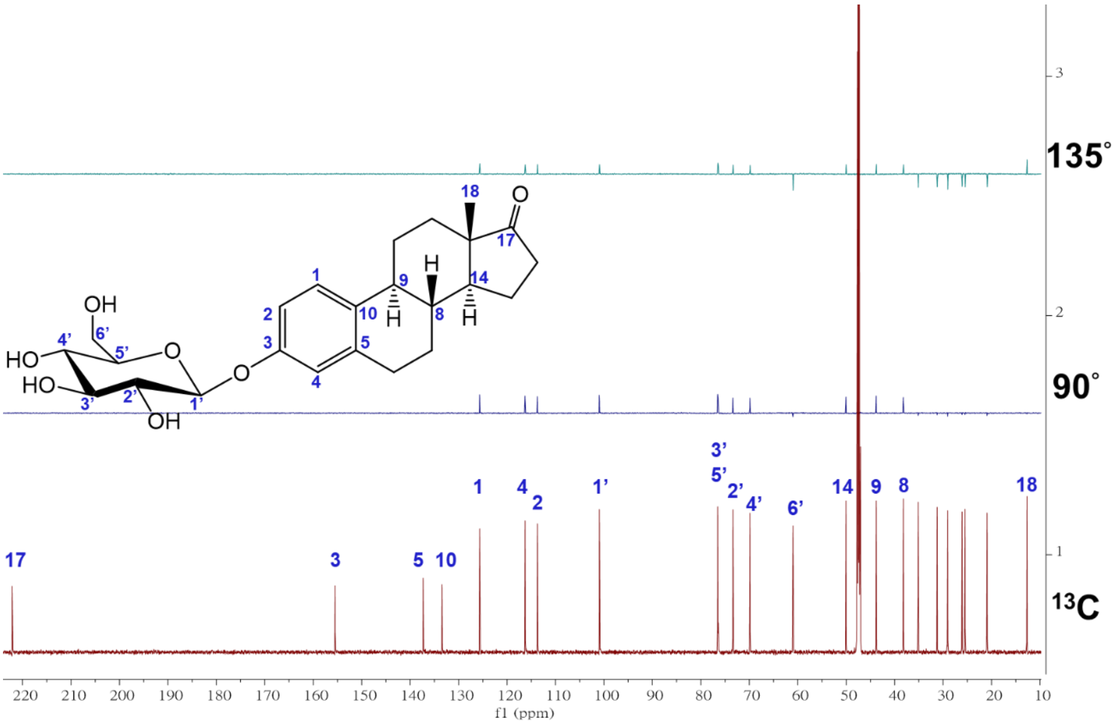

(D) ^{1}H-^{1}H COSY

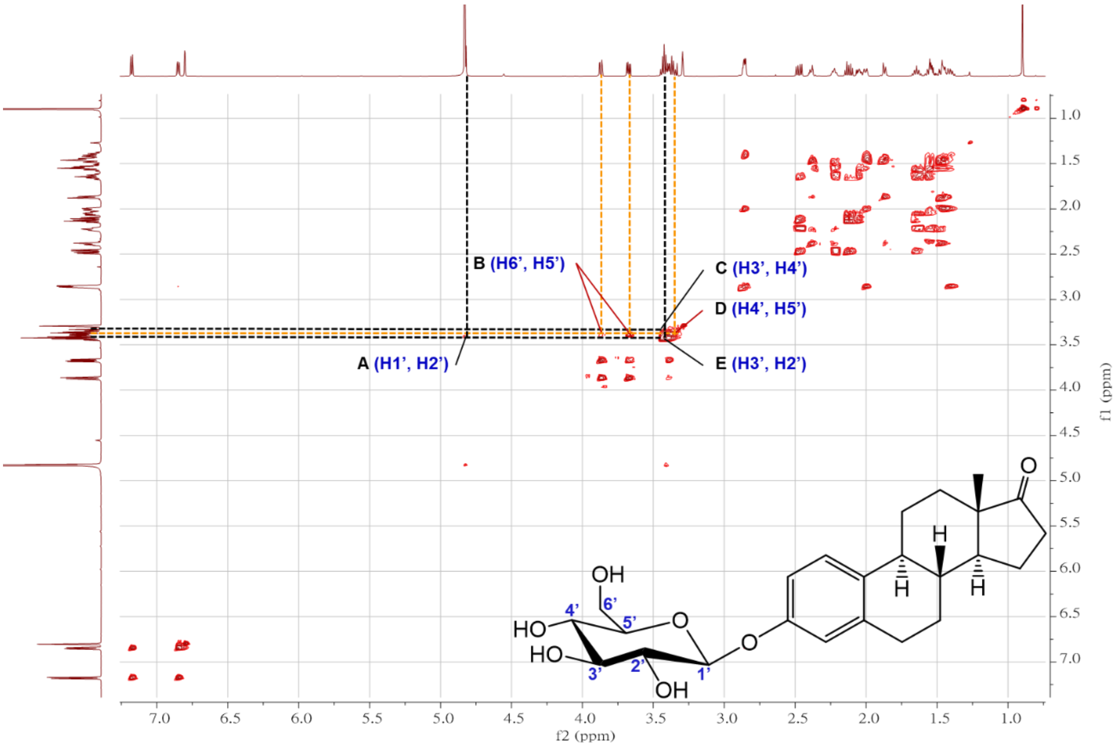

(E) HSQC

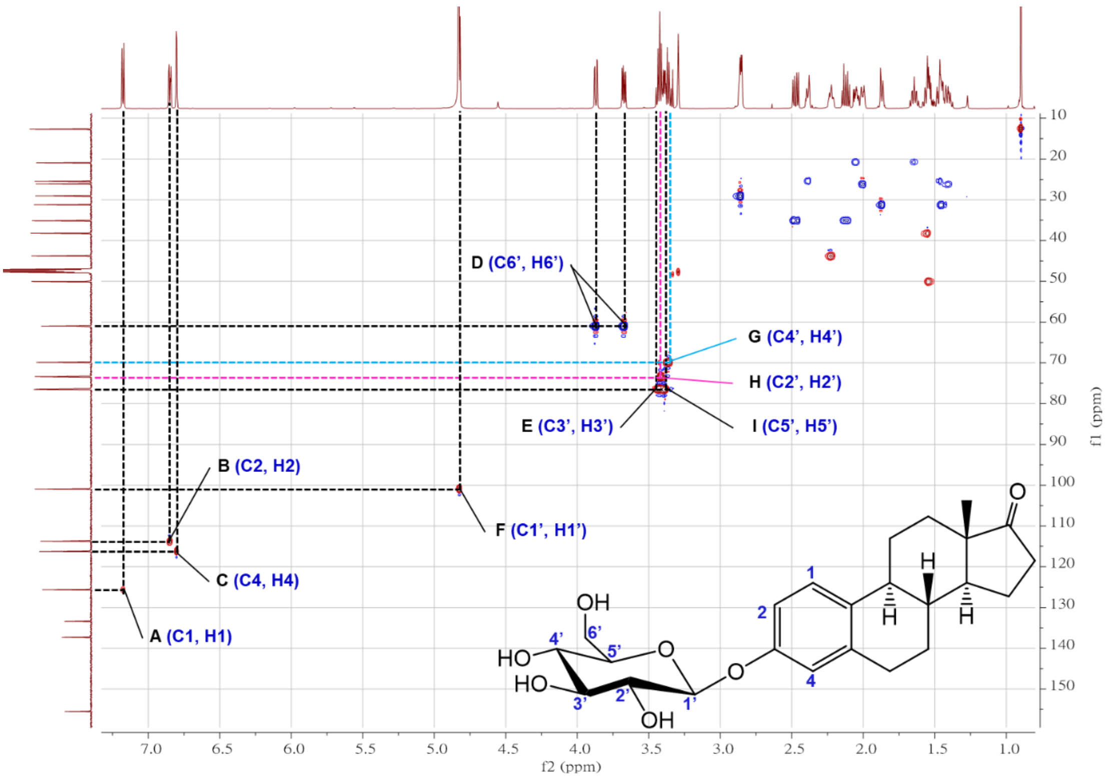

D (C6', H6')
G (C4', H4')
H (C2', H2')
E (C3', H3')
I (C5', H5')
B (C2, H2)
F (C1', H1')
C (C4, H4)
A (C1, H1)
f1 (ppm)
f2 (ppm)

(F) HMBC

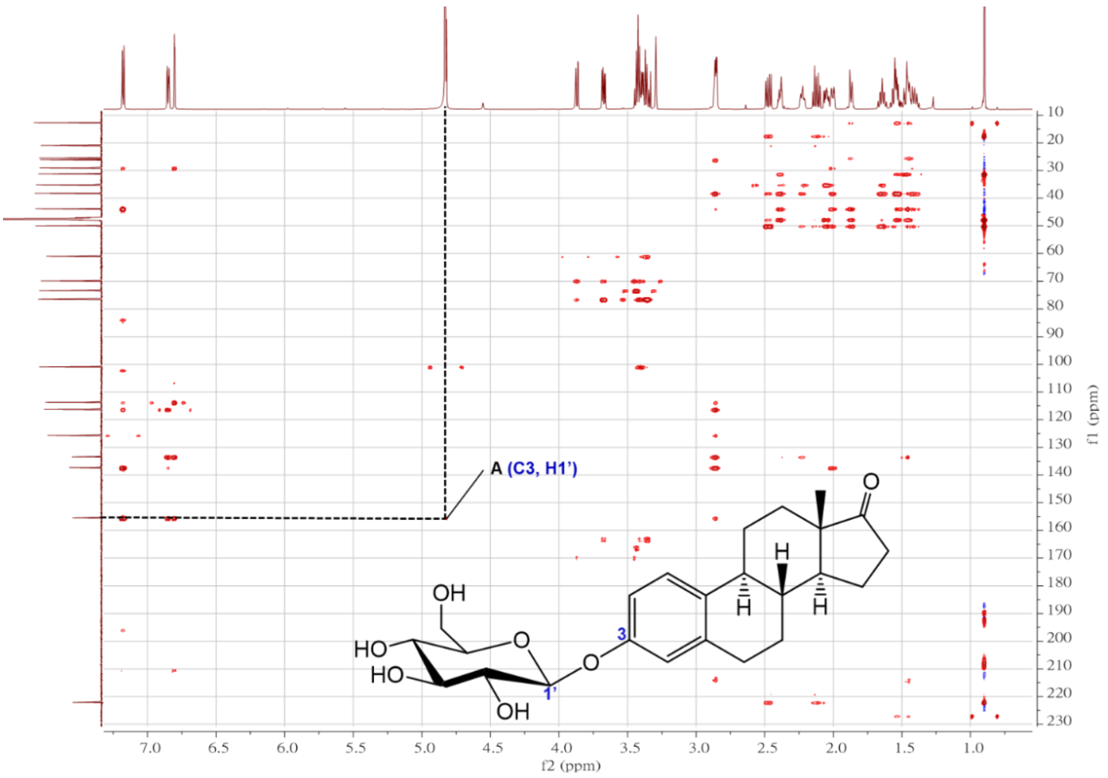

A (C3, H1')
f1 (ppm)
f2 (ppm)

Fig. S6 High–resolution UPLC/ESI MS and NMR of E1-α-2DG (**2b**). (A) ^{1}H NMR, (B) ^{13}C NMR, (C) DEPT, (D) ^{1}H-^{1}H COSY, (E) HSQC, and (F) HMBC.

(A) ^{1}H NMR

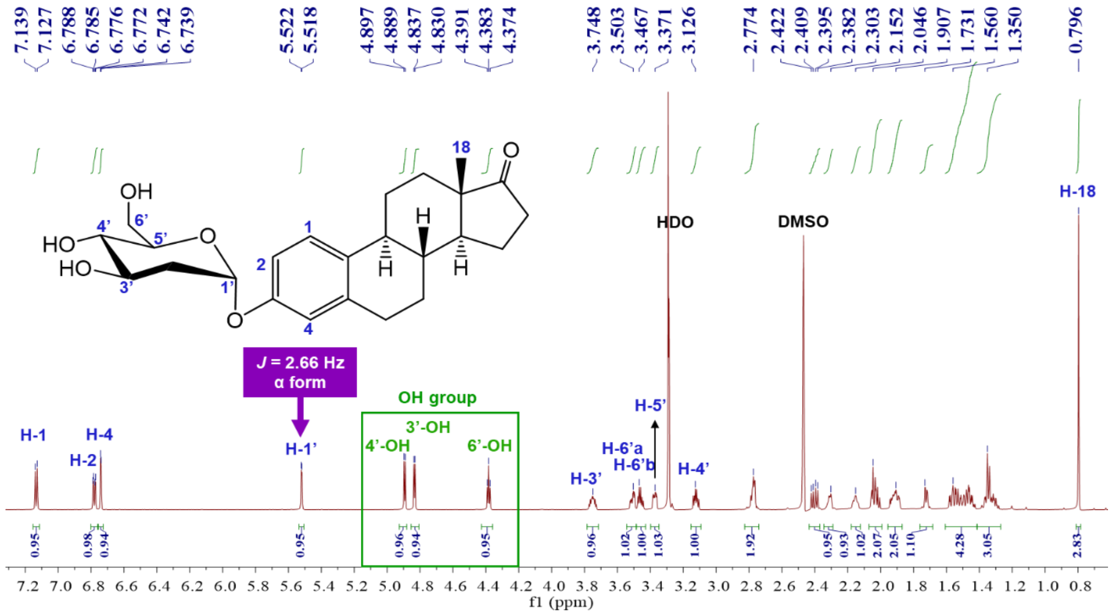

(B) ^{13}C NMR

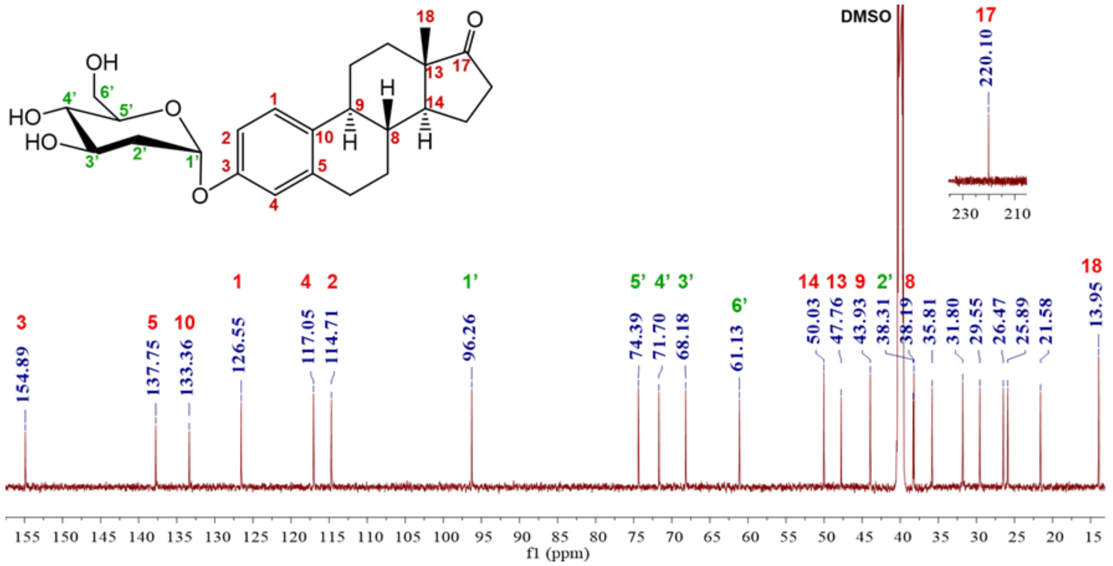

(C) DEPT

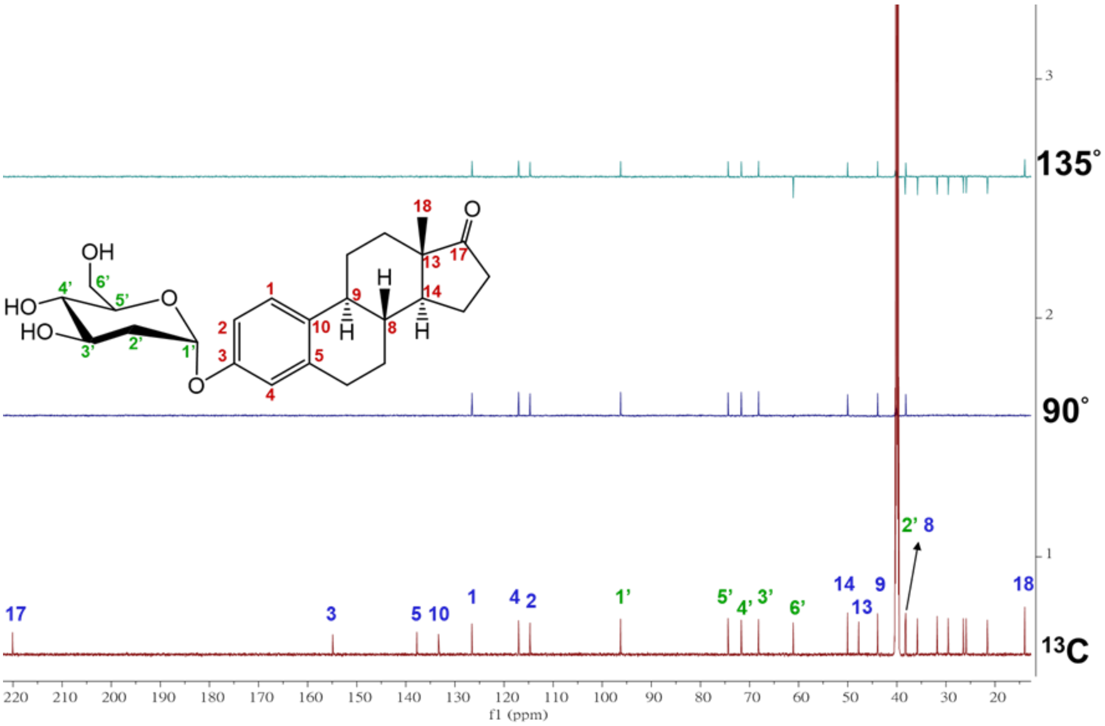

(D) ^{1}H-^{1}H COSY

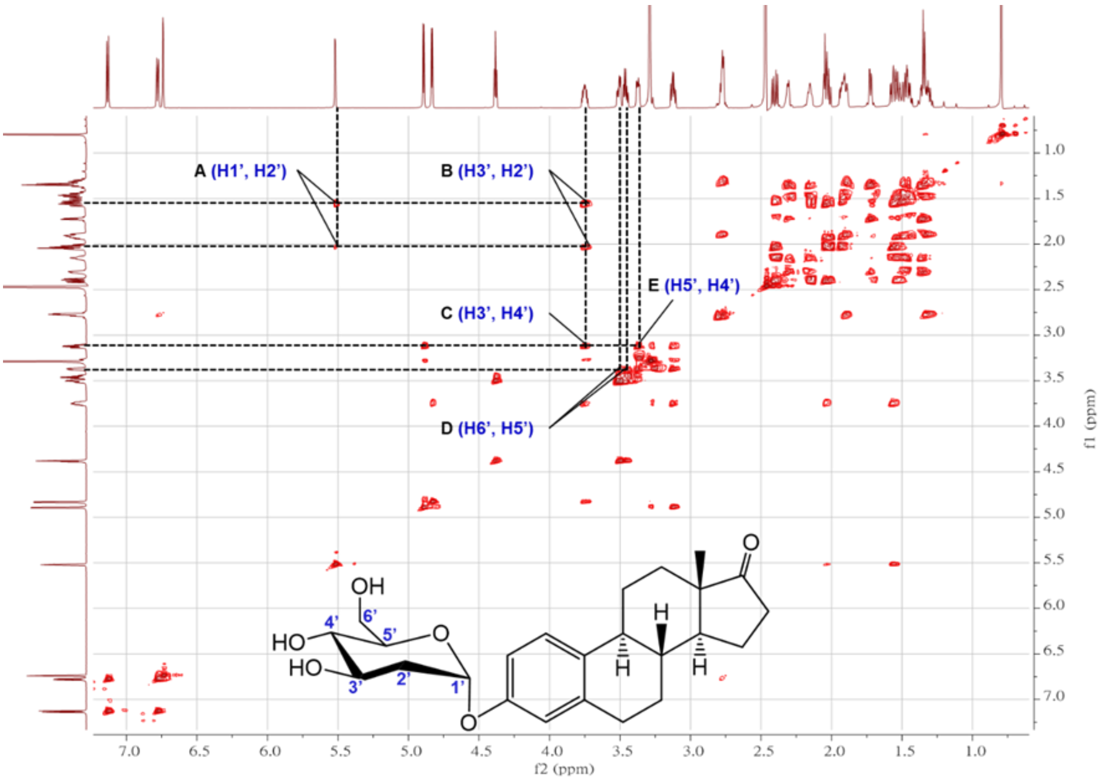

(E) HSQC

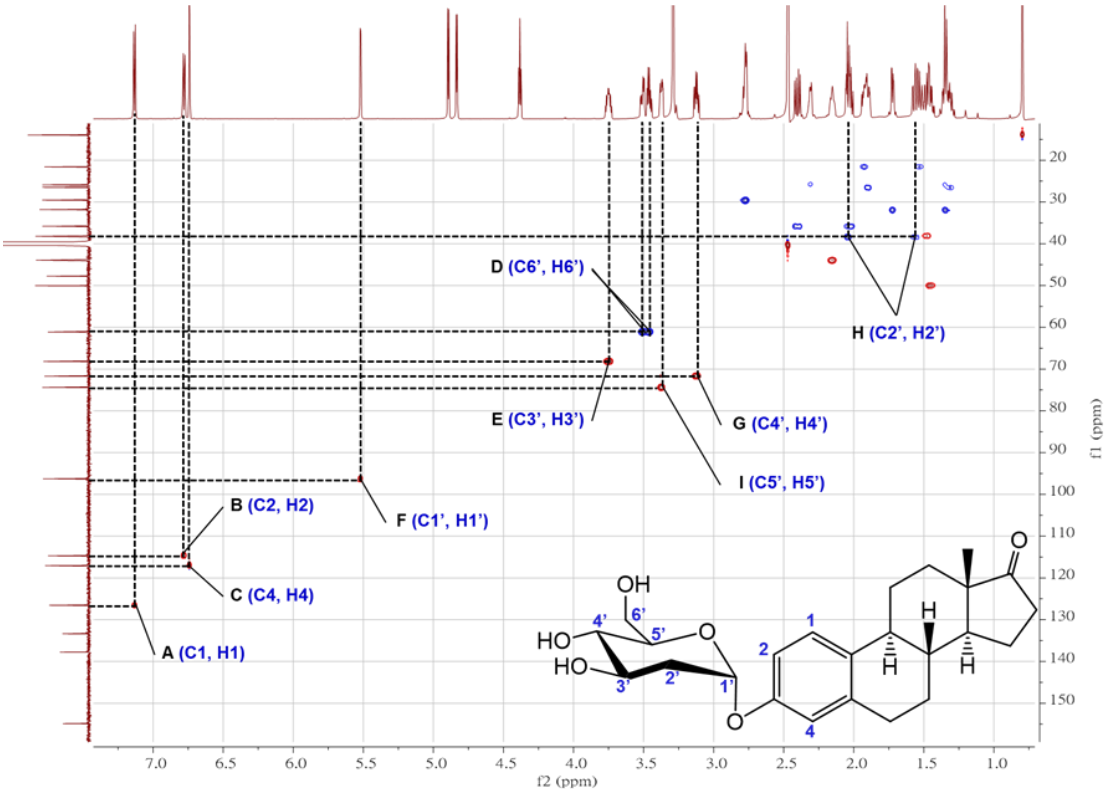

A (C1, H1)
B (C2, H2)
C (C4, H4)
D (C6', H6')
E (C3', H3')
F (C1', H1')
G (C4', H4')
H (C2', H2')
I (C5', H5')
f1 (ppm)
f2 (ppm)

(F) HMBC

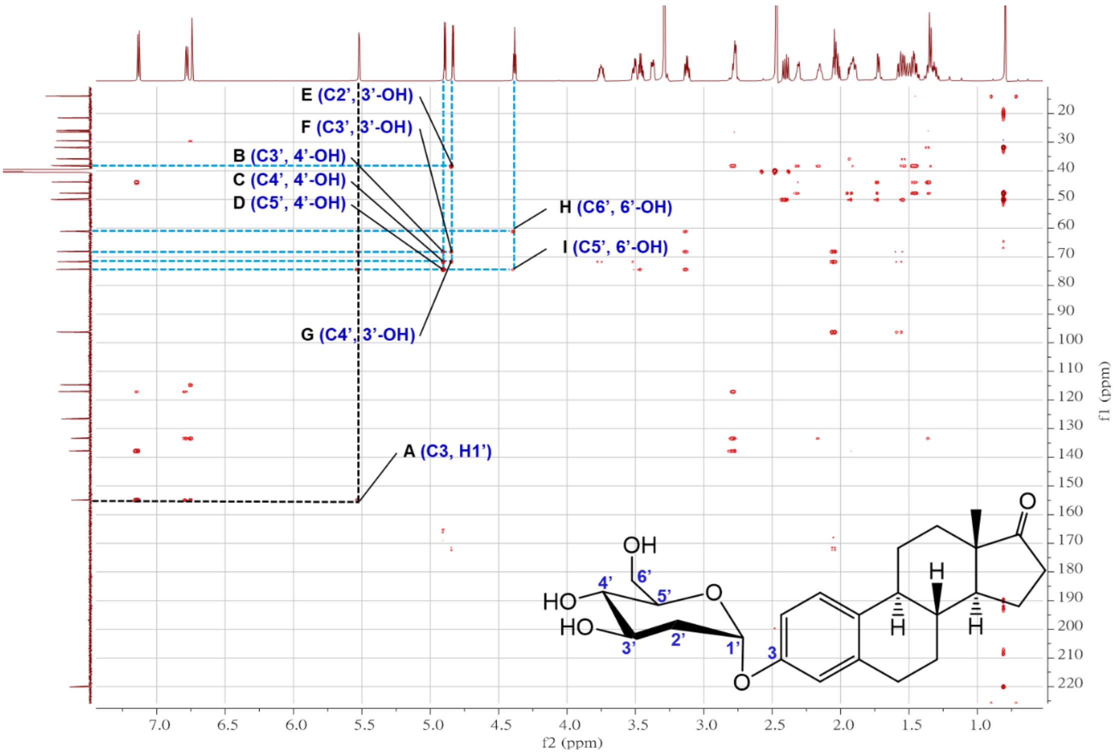

E (C2', 3'-OH)
F (C3', 3'-OH)
B (C3', 4'-OH)
C (C4', 4'-OH)
D (C5', 4'-OH)
H (C6', 6'-OH)
I (C5', 6'-OH)
G (C4', 3'-OH)
A (C3, H1')
f1 (ppm)
f2 (ppm)

Fig. S7 High–resolution UPLC/ESI MS and NMR of E1-β-2DG (**2c**). (A) ^{1}H NMR, (B) ^{13}C NMR, (C) DEPT, (D) ^{1}H-^{1}H COSY, (E) HSQC, and (F) HMBC.

(A) ^{1}H NMR

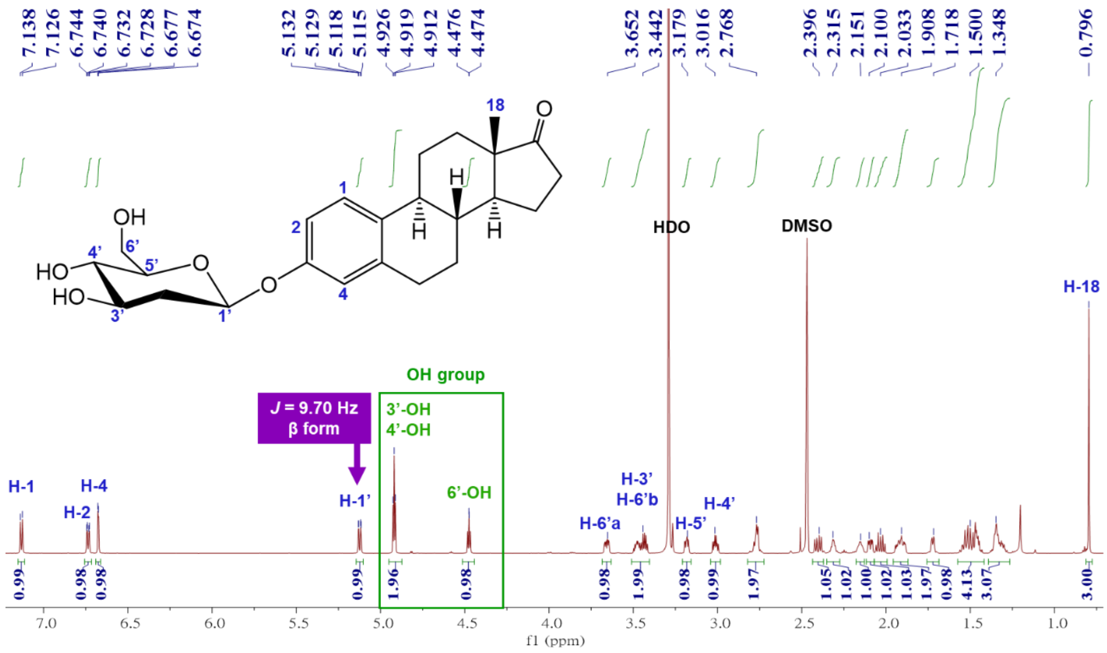

(B) ^{13}C NMR

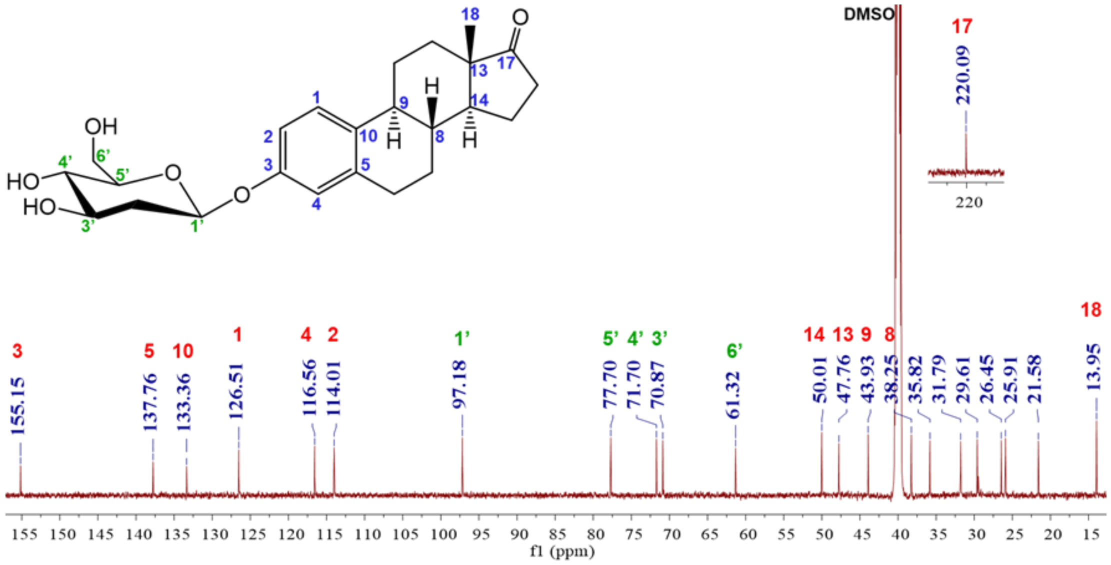

(C) DEPT

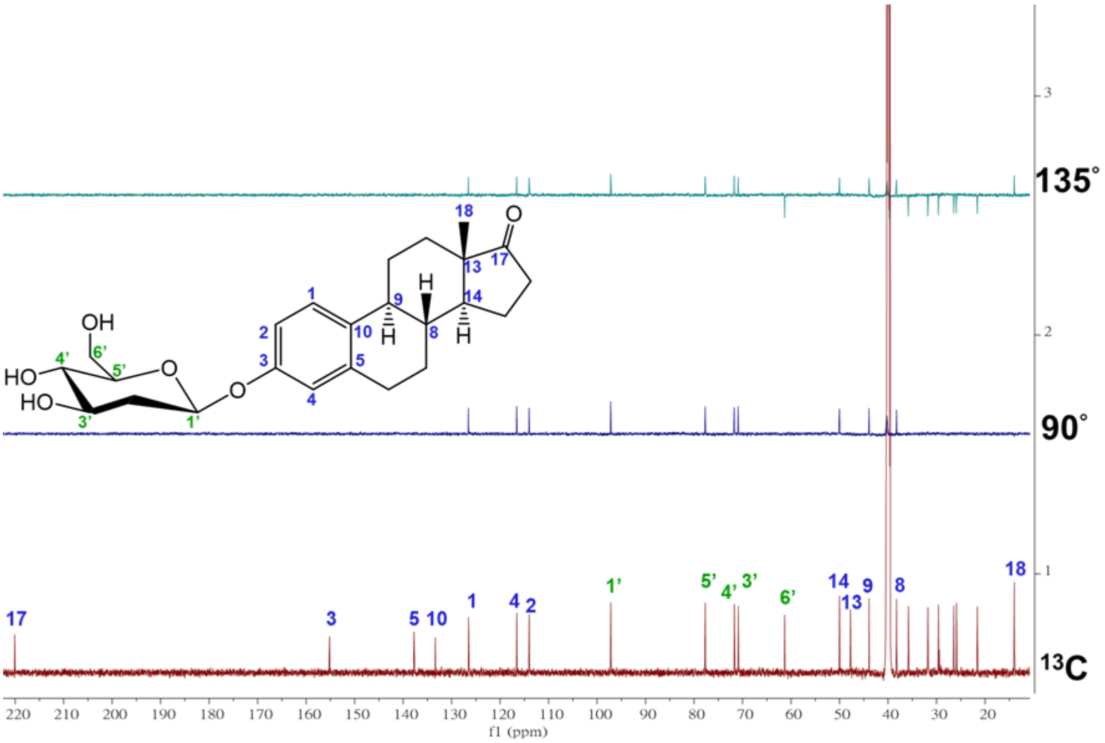

(D) ^{1}H-^{1}H COSY

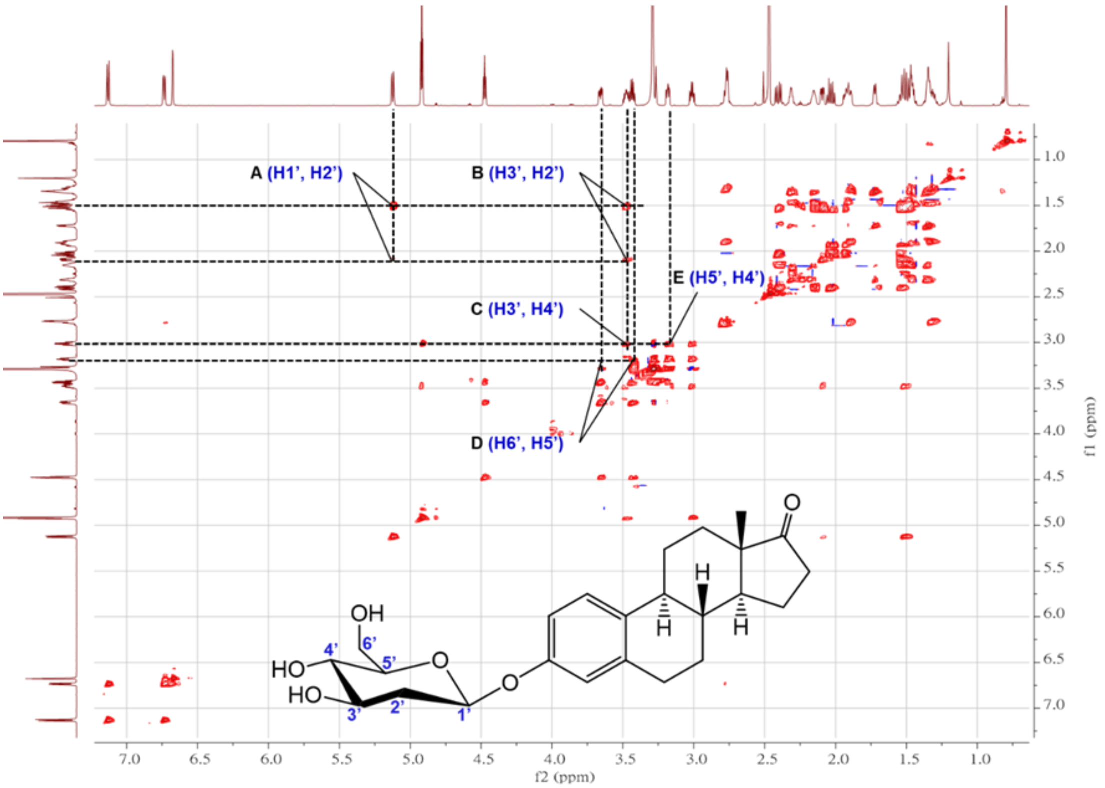

(E) HSQC

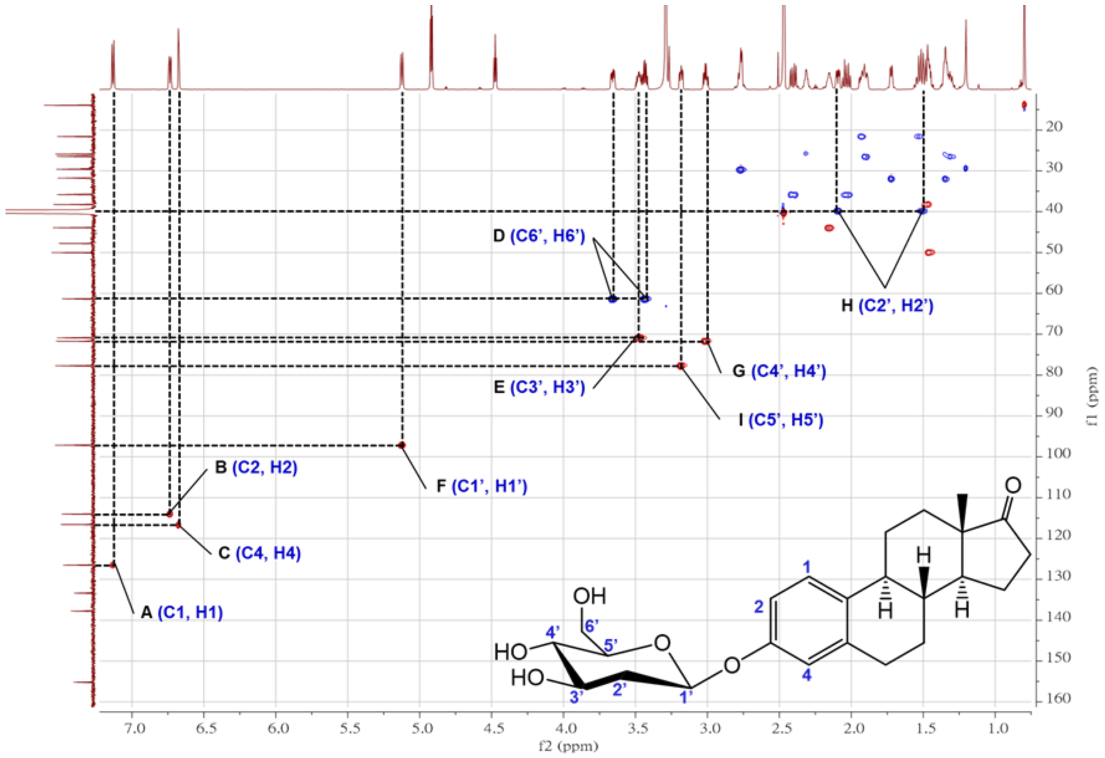
D (C6', H6')
H (C2', H2')
G (C4', H4')
E (C3', H3')
I (C5', H5')
F (C1', H1')
B (C2, H2)
C (C4, H4)
A (C1, H1)
f2 (ppm)
f1 (ppm)

(F) HMBC

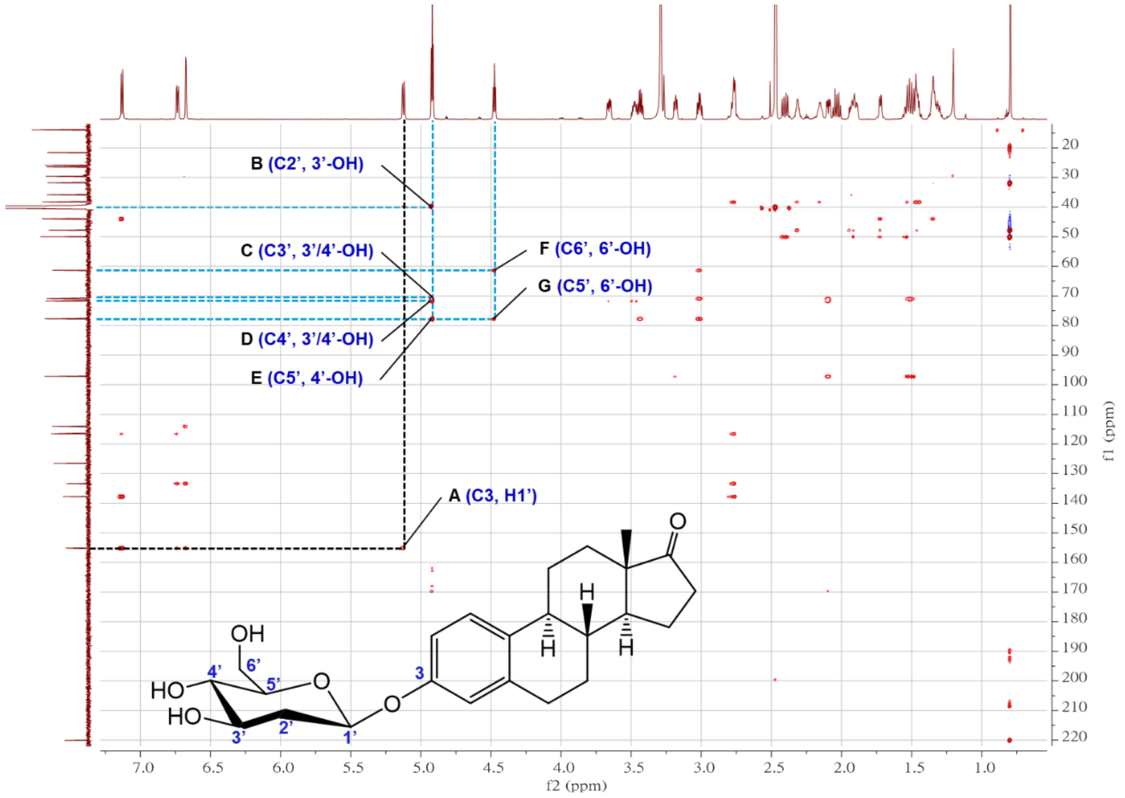
B (C2', 3'-OH)
C (C3', 3'/4'-OH)
F (C6', 6'-OH)
G (C5', 6'-OH)
D (C4', 3'/4'-OH)
E (C5', 4'-OH)
A (C3, H1')
f2 (ppm)
f1 (ppm)

Fig. S8 High–resolution UPLC/ESI MS and NMR of E2-β-Glc (**3a**). (A) ^{1}H NMR, (B) ^{13}C NMR, (C) DEPT, (D) ^{1}H-^{1}H COSY, (E) HSQC, and (F) HMBC.

(A) ^{1}H NMR

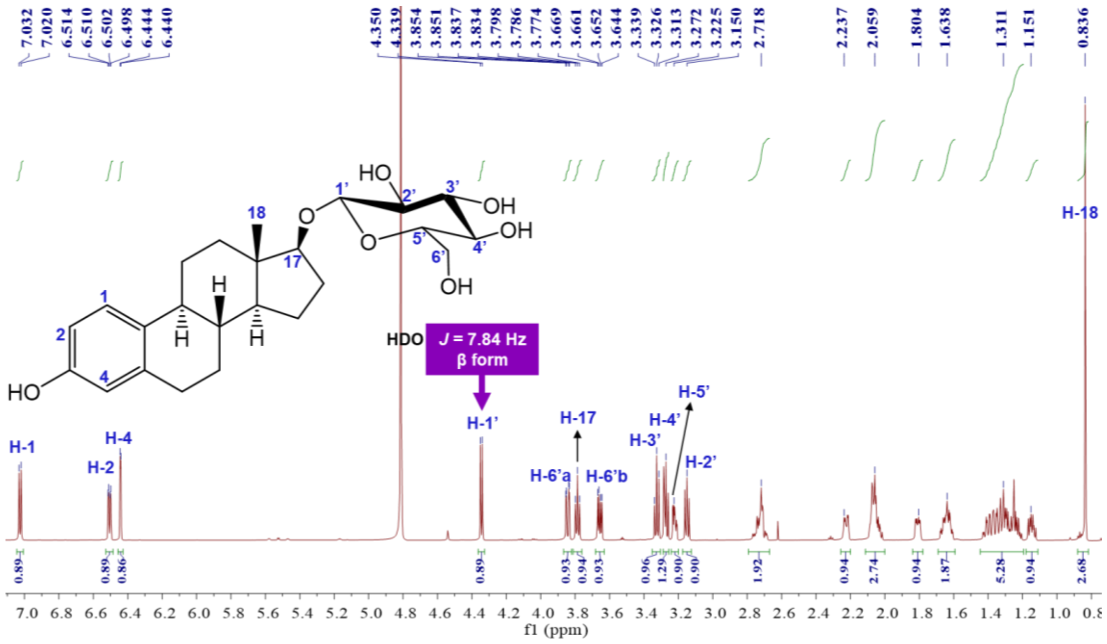

(B) ^{13}C NMR

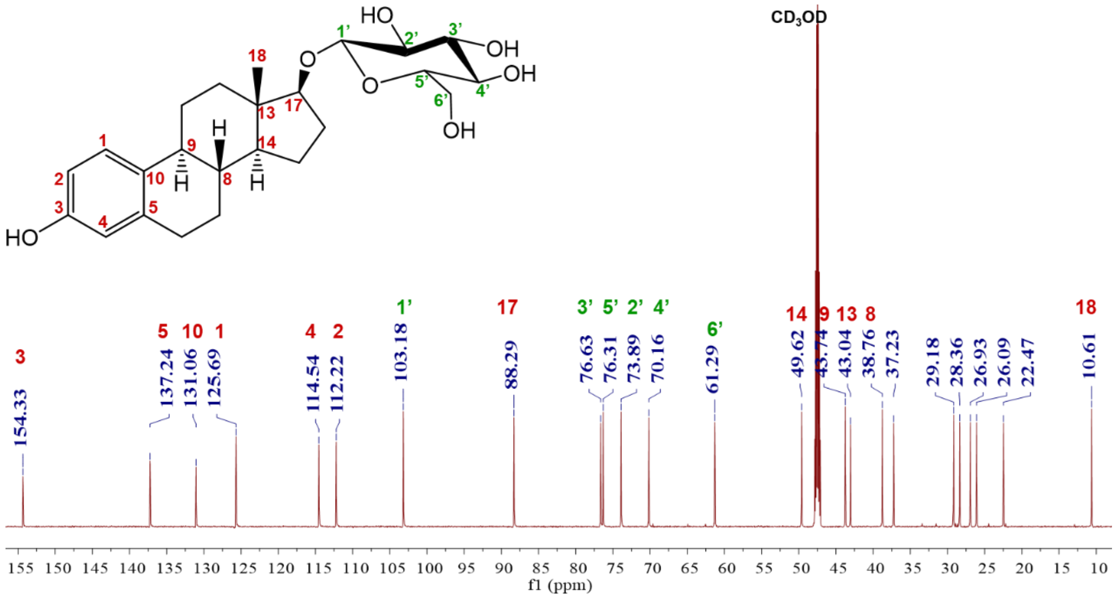

(C) DEPT

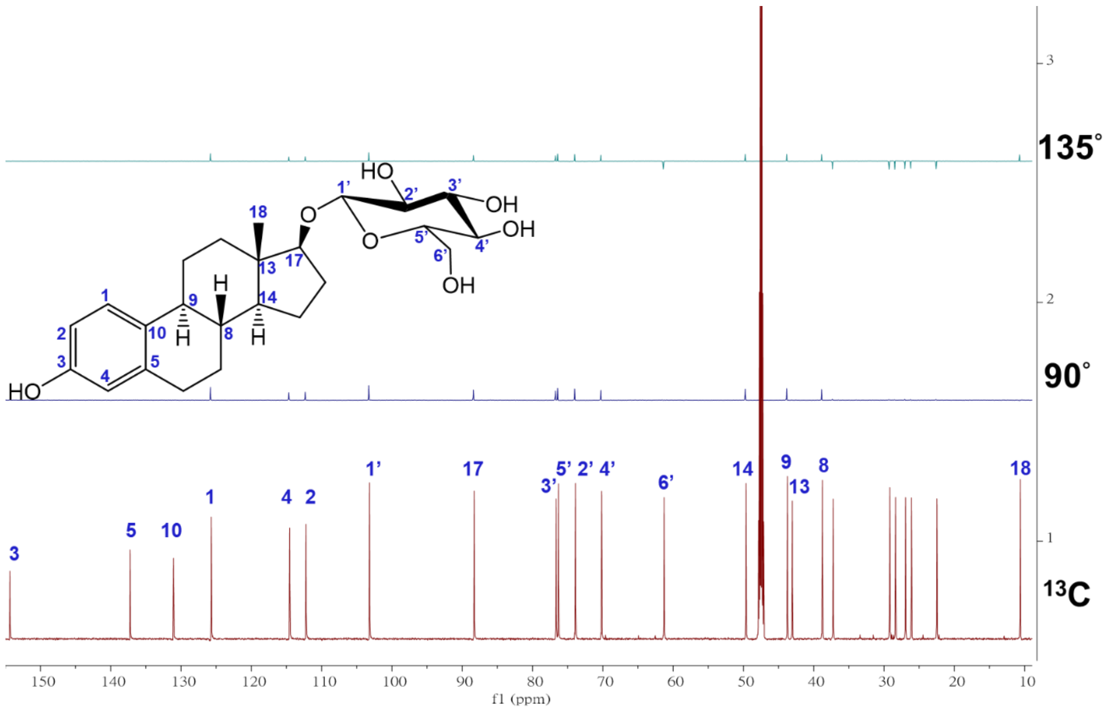

(D) ^{1}H-^{1}H COSY

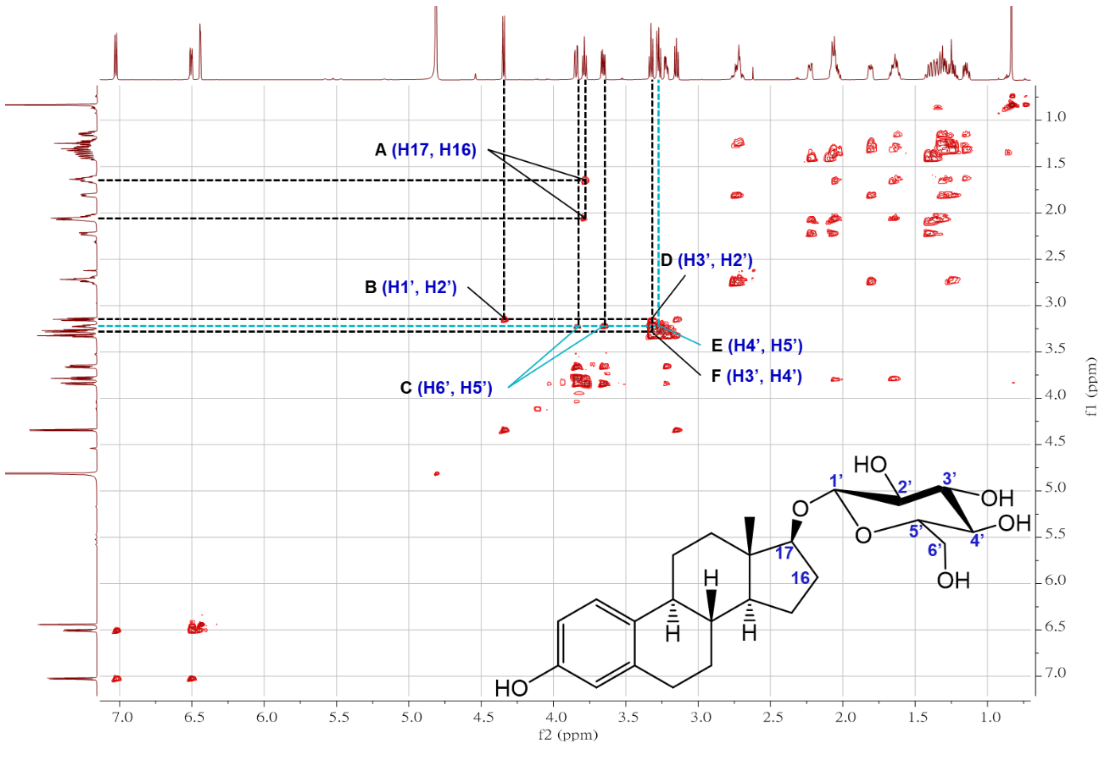

(E) HSQC

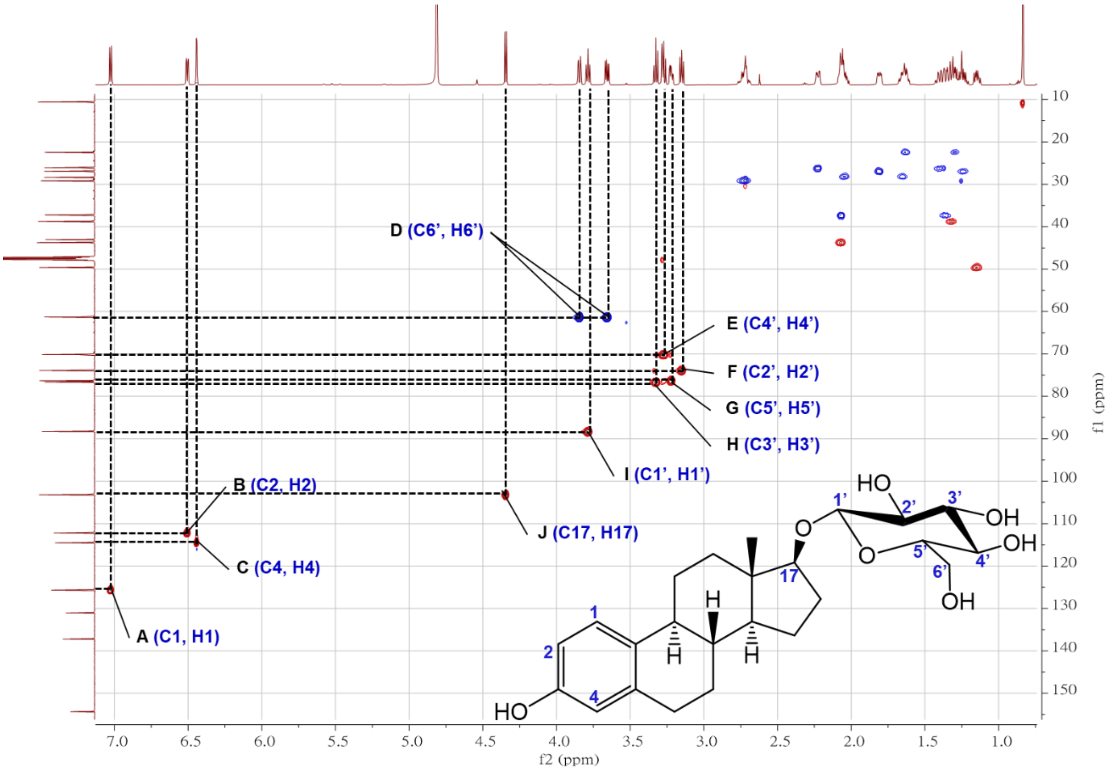
D (C6', H6')
E (C4', H4')
F (C2', H2')
G (C5', H5')
H (C3', H3')
I (C1', H1')
B (C2, H2)
J (C17, H17)
C (C4, H4)
A (C1, H1)
f1 (ppm)
f2 (ppm)

(F) HMBC

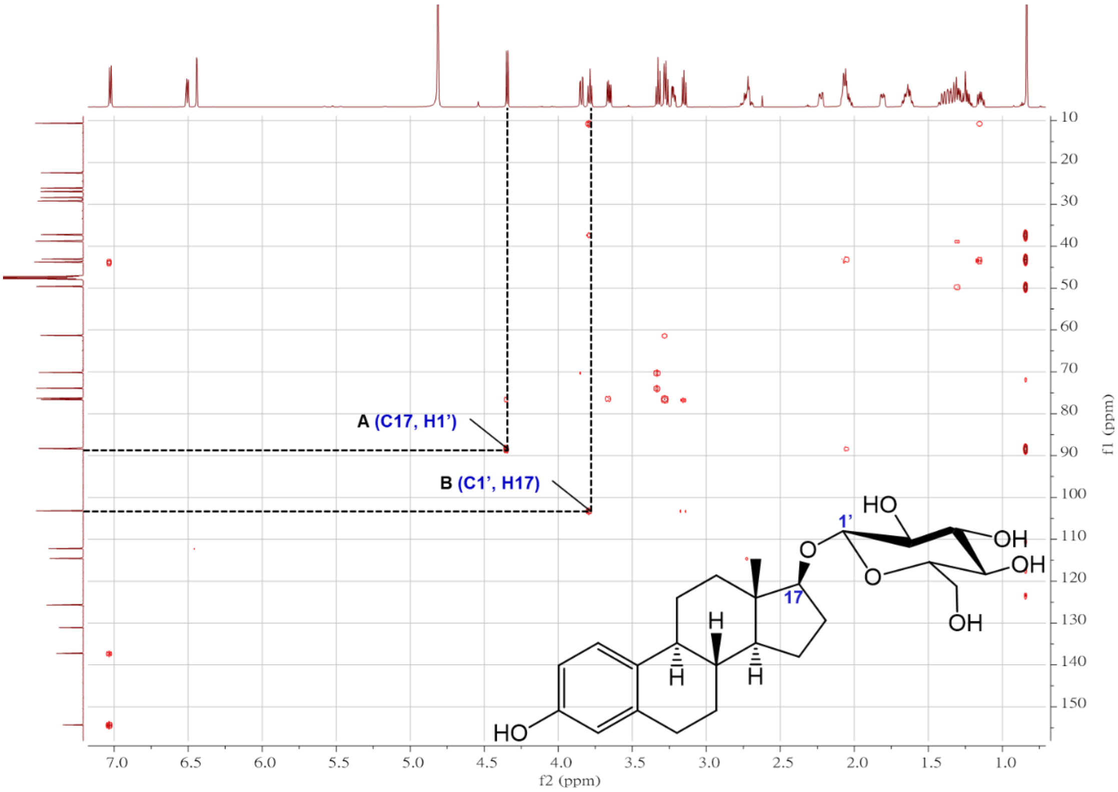
A (C17, H1')
B (C1', H17)
f1 (ppm)
f2 (ppm)

Fig. S9 High–resolution UPLC/ESI MS and NMR of E2-α-2DG (**3b**). (A) ^{1}H NMR, (B) ^{13}C NMR, (C) DEPT, (D) ^{1}H-^{1}H COSY, (E) HSQC, and (F) HMBC.

(A) ^{1}H NMR

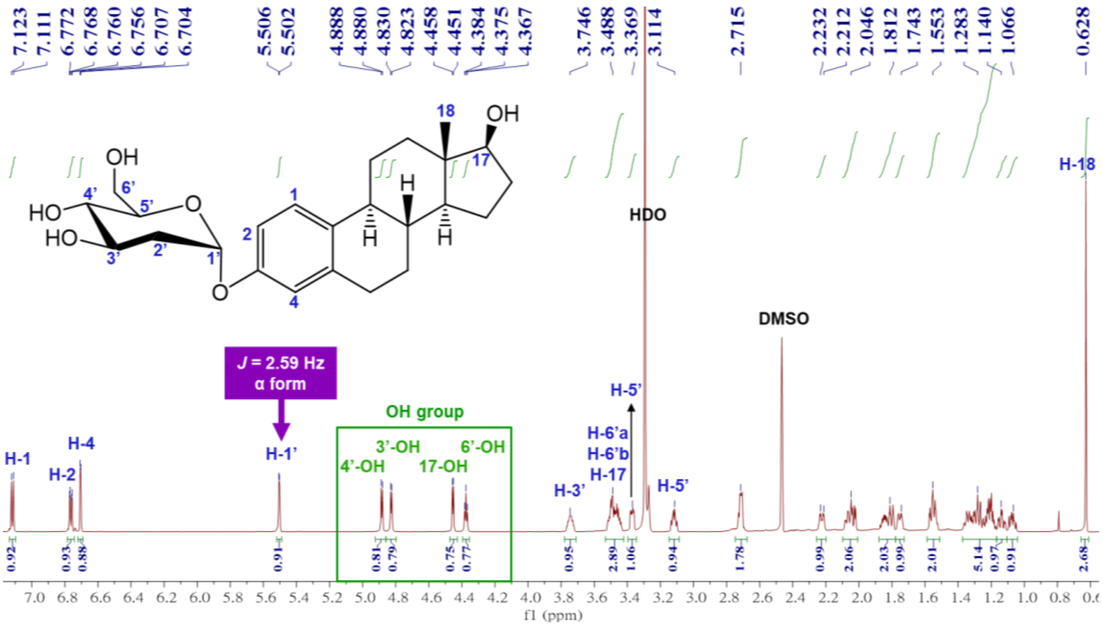

(B) ^{13}C NMR

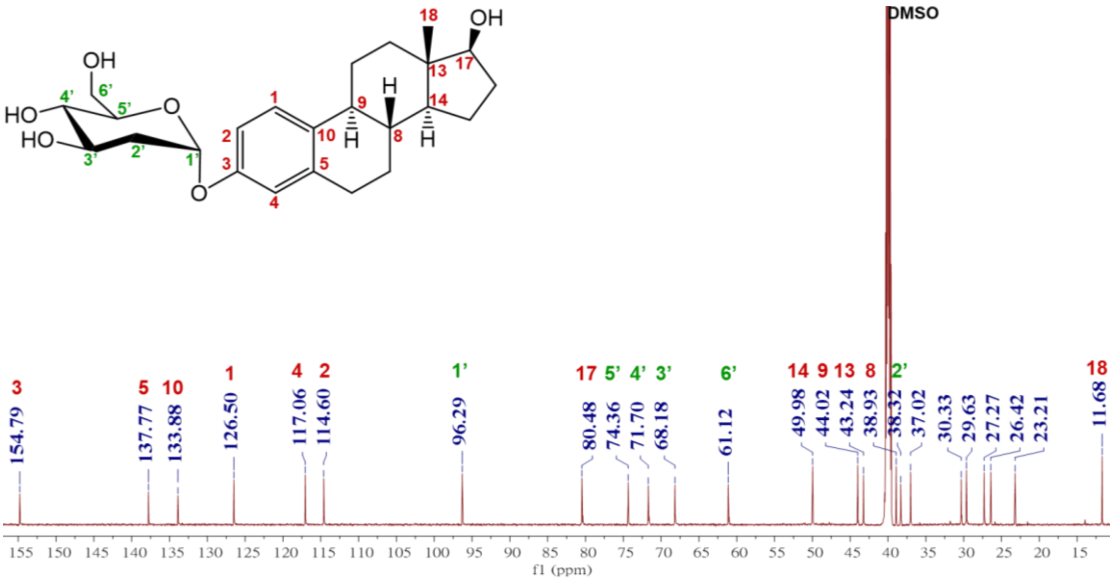

(C) DEPT

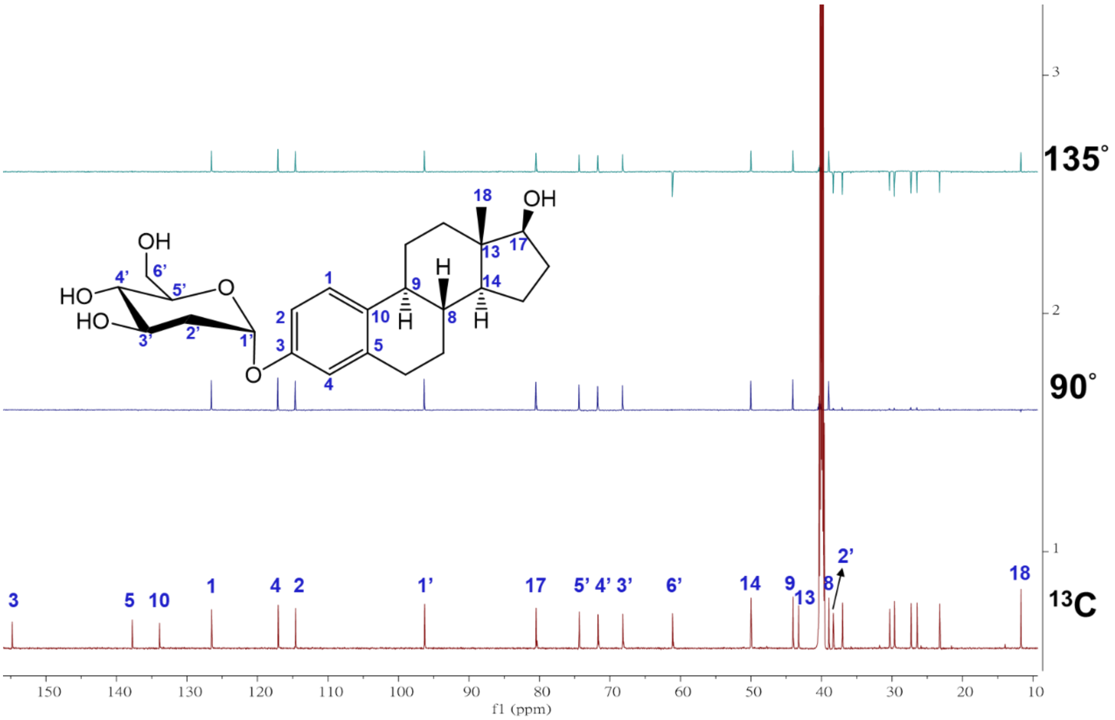

(D) ^{1}H-^{1}H COSY

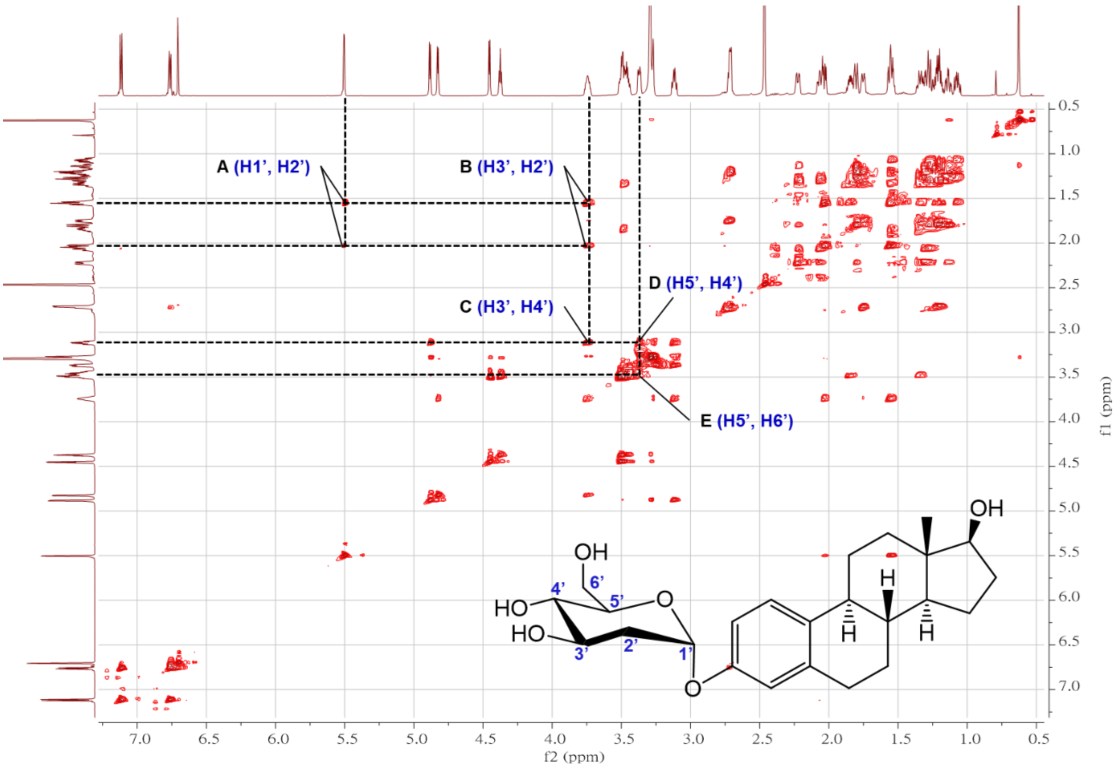

(E) HSQC

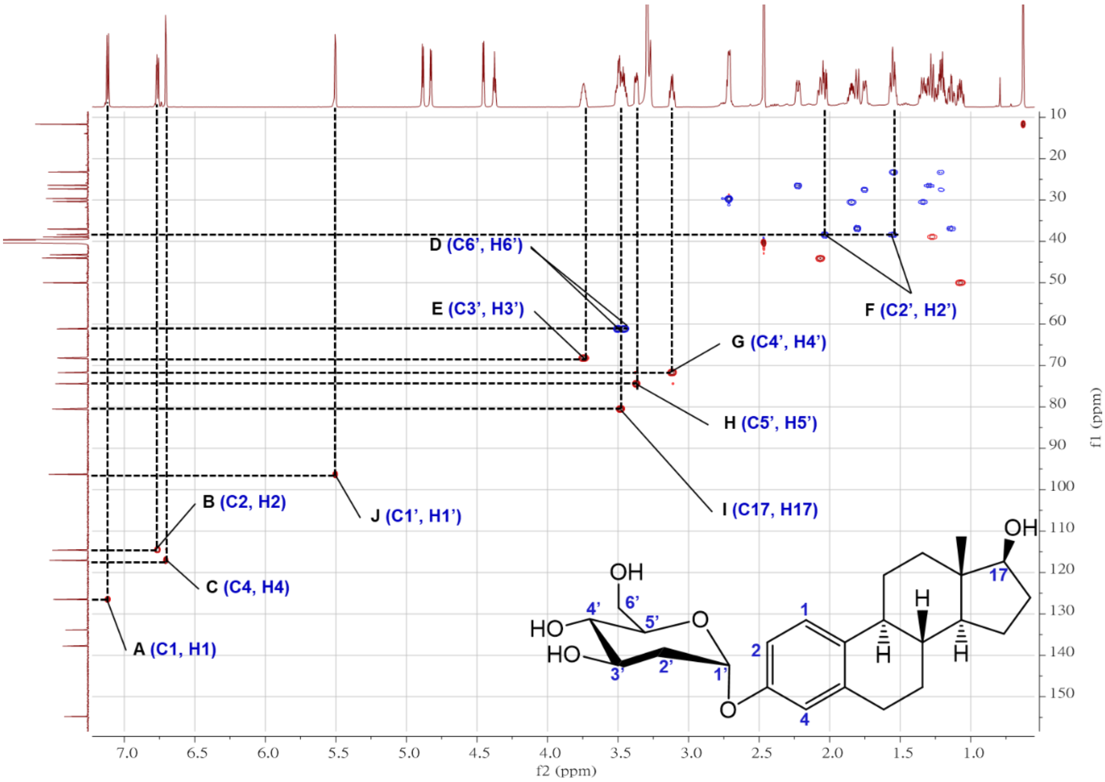
A (C1, H1)
B (C2, H2)
C (C4, H4)
D (C6', H6')
E (C3', H3')
F (C2', H2')
G (C4', H4')
H (C5', H5')
I (C17, H17)
J (C1', H1')
f2 (ppm)
f1 (ppm)

(F) HMBC

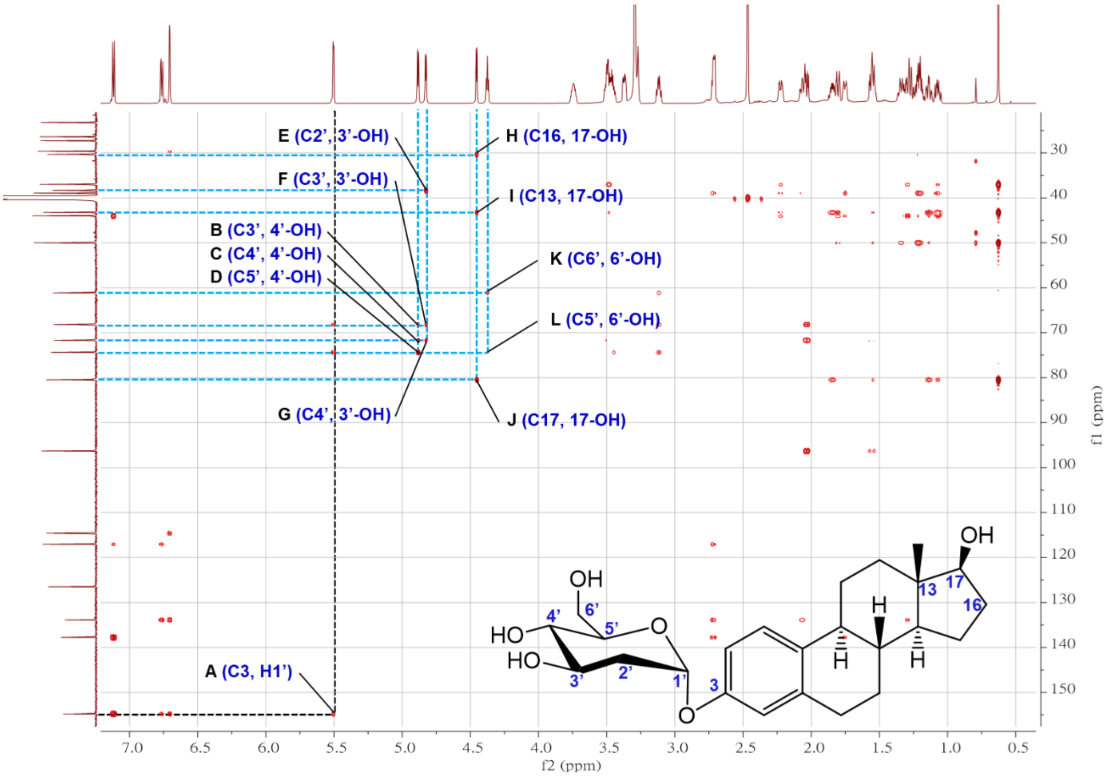
A (C3, H1')
B (C3', 4'-OH)
C (C4', 4'-OH)
D (C5', 4'-OH)
E (C2', 3'-OH)
F (C3', 3'-OH)
G (C4', 3'-OH)
H (C16, 17-OH)
I (C13, 17-OH)
J (C17, 17-OH)
K (C6', 6'-OH)
L (C5', 6'-OH)
f2 (ppm)
f1 (ppm)

Fig. S10 High–resolution UPLC/ESI MS and NMR of E2-β-2DG (**3c**). (A) ^{1}H NMR, (B) ^{13}C NMR, (C) DEPT, (D) ^{1}H-^{1}H COSY, (E) HSQC, and (F) HMBC.

(A) ^{1}H NMR

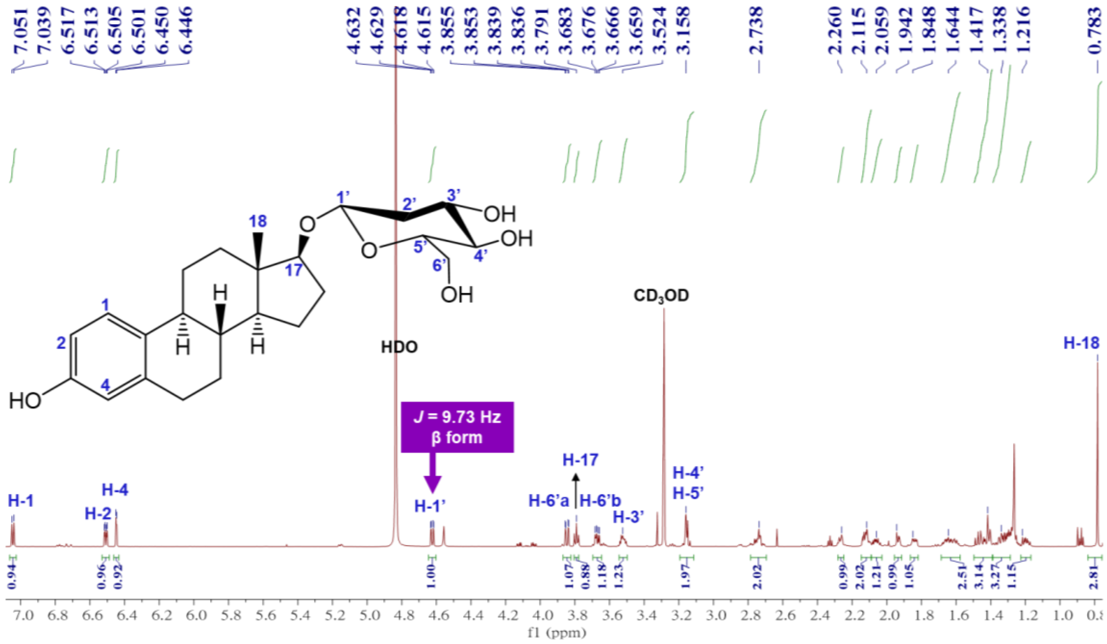

(B) ^{13}C NMR

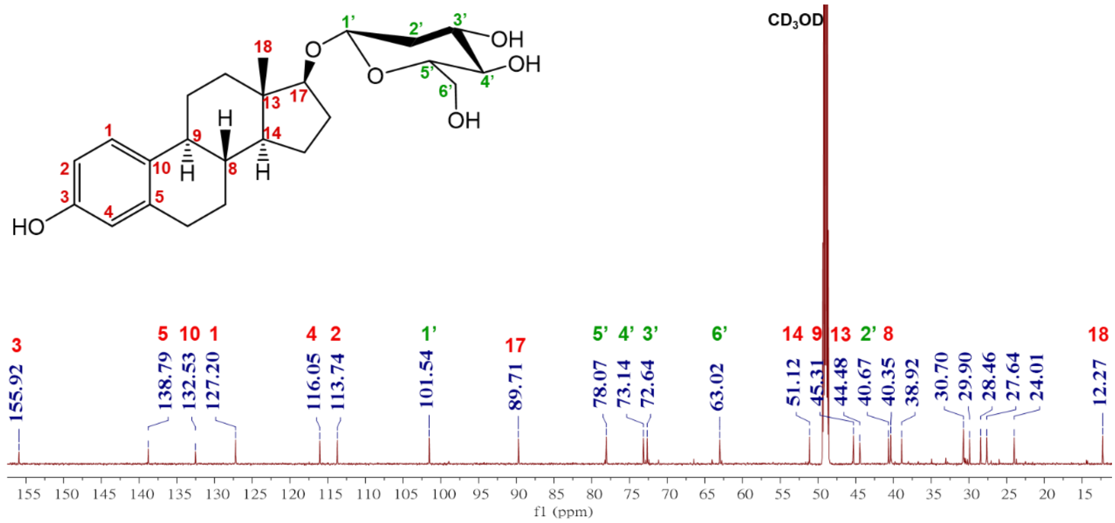

(C) DEPT

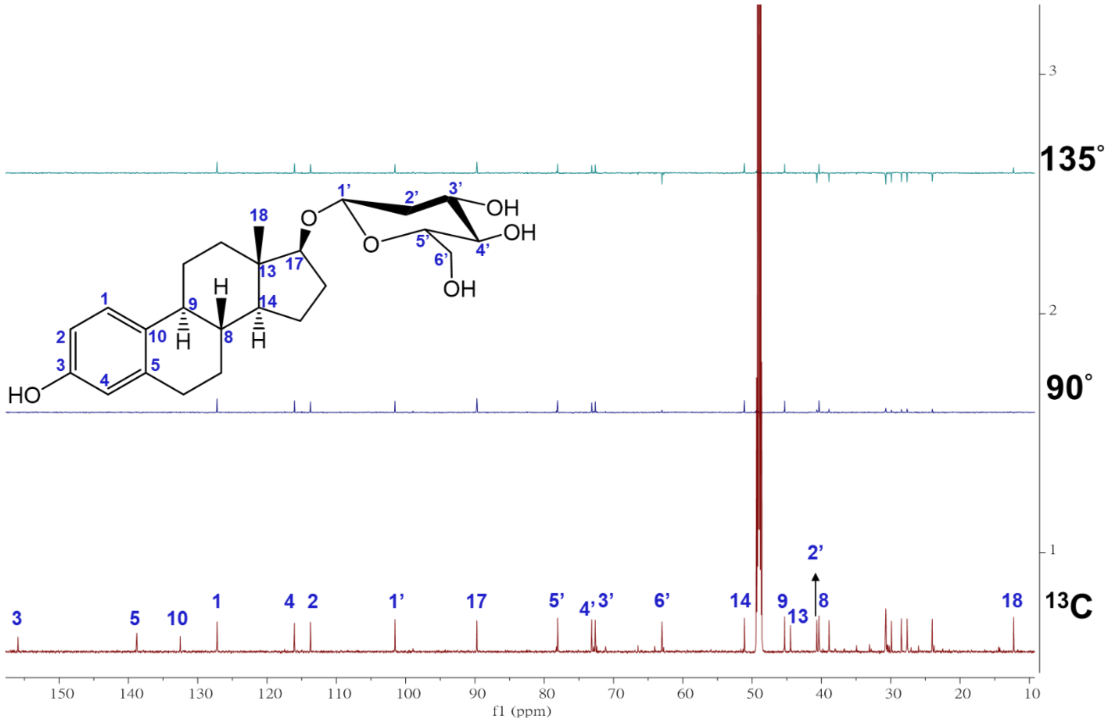

(D) ^{1}H-^{1}H COSY

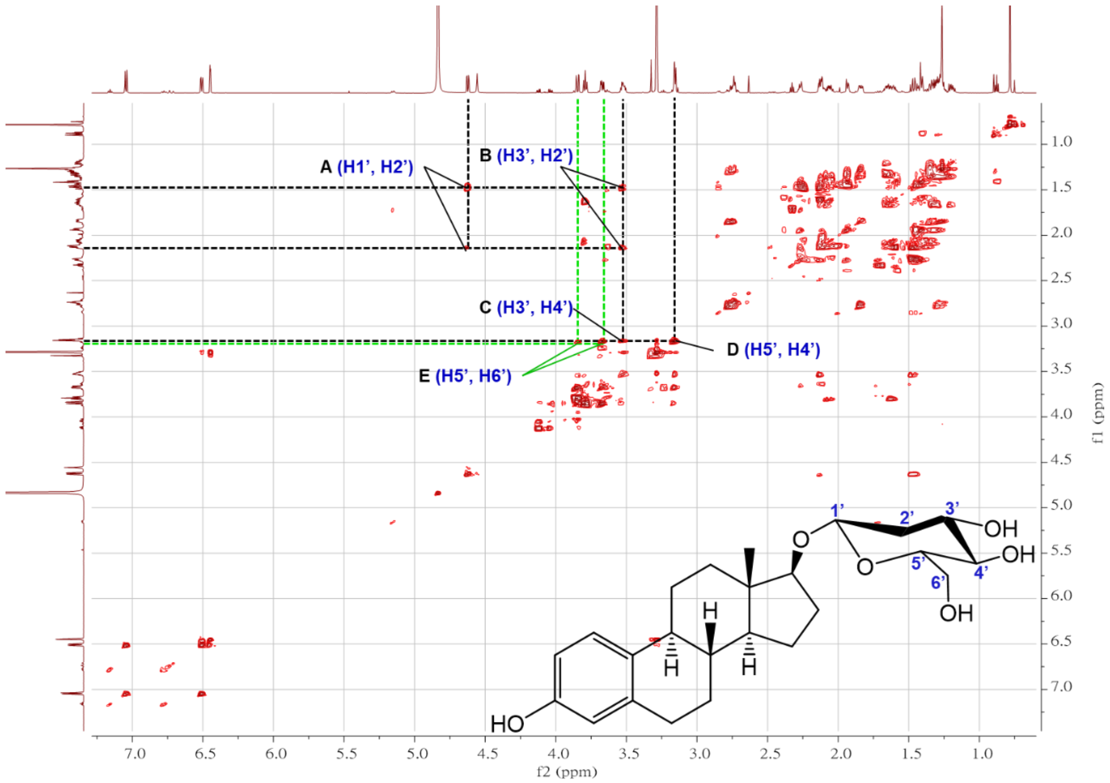

(E) HSQC

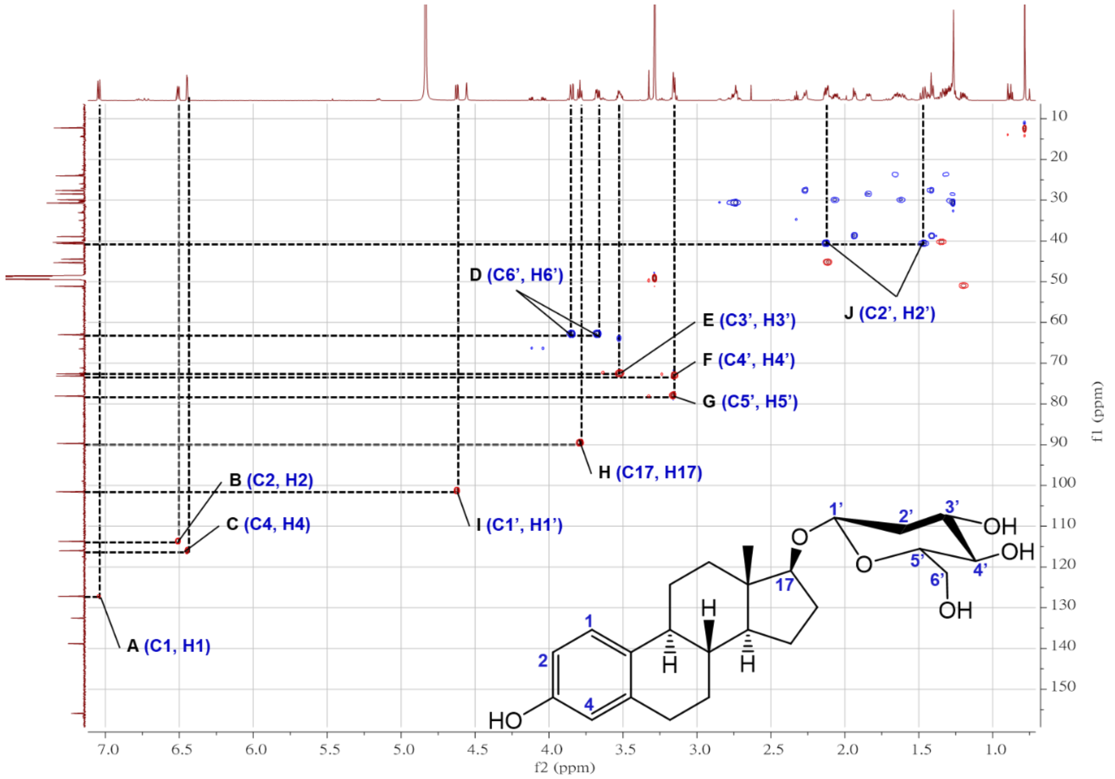
D (C6', H6')
E (C3', H3')
F (C4', H4')
G (C5', H5')
H (C17, H17)
J (C2', H2')
B (C2, H2)
C (C4, H4)
I (C1', H1')
A (C1, H1)
f1 (ppm)
f2 (ppm)

(F) HMBC

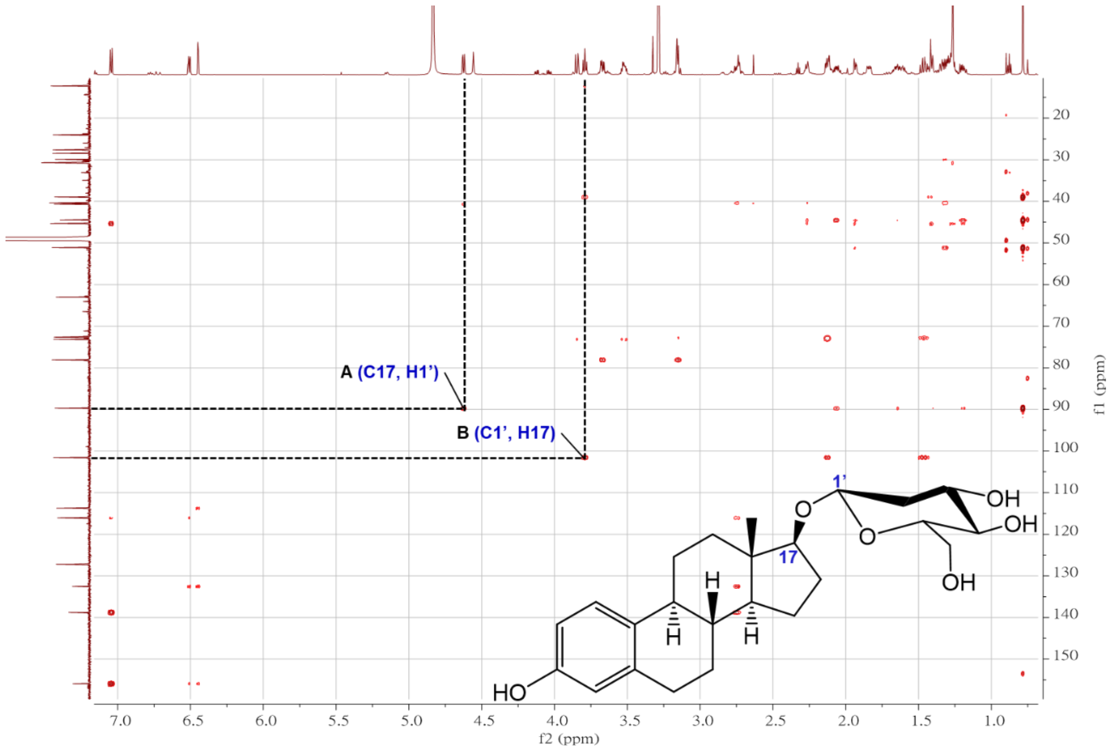
A (C17, H1')
B (C1', H17)
f1 (ppm)
f2 (ppm)

Table S1. Cell viability of *t*AND (**1**), E1 (**2**), E2 (**3**), 2-ME (**4**), and *trans*-androsterone-, estrone-, and estradiol-glycoside (**1a–3c**) against different breast cancer cell lines.

Compound	IC_{50} (µM)		
	**MCF-7**	**MDA-MB-231**	**HEK293**
2-Methoxyestradiol (2ME, **4**)	53.41 ± 3.73	28.50 ± 3.74	73.19 ± 7.56
*t*AND (**1**)	> 100	71.22 ± 1.39	> 100
*t*AND-α-Glc (**1a**)	> 100	> 100	> 100
*t*AND-β-Glc (**1b**)	> 100	> 100	> 100
*t*AND-α-2DG (**1c**)	> 100	> 100	> 100
*t*AND-β-2DG (**1d**)	> 100	> 100	> 100
Estrone (E1, **2**)	> 100	> 100	> 100
E1-β-Glc (**2a**)	> 100	> 100	> 100
E1-α-2DG (**2b**)	61.79 ± 2.87	20.46 ± 2.92	> 100
E1-β-2DG (**2c**)	> 100	68.27 ± 4.75	> 100
Estradiol (E2, **3**)	> 100	88.53 ± 11.90	> 100
E2-β-Glc (**3a**)	> 100	>100	> 100
E2-α-2DG (**3b**)	88.41 ± 12.83	62.96 ± 2.52	> 100
E2-β-2DG (**3c**)	> 100	63.92 ± 2.44	> 100

Table S2. Inhibitory activities of *t*AND (**1**), E1 (**2**), E2 (**3**), 2-ME (**4**), and *trans*-androsterone-, estrone-, and estradiol-glycoside (**1a–3c**) against aromatase CYP19.

**Compound**	**IC_{50} (μM)**
	Aromatase
Exemestane (EXM)	0.045 ± 0.001
Pregnenolone	>100
tAND (1)	28.808 ± 1.290
*t*AND-α-Glc (1a)	> 100
*t*AND-β-Glc (1b)	2.332 ± 0.077
*t*AND-α-2DG (1c)	> 100
*t*AND-β-2DG (1d)	1.161 ± 0.014
E1-β-Glc (2a)	0.775 ± 0.040
E1-α-2DG (2b)	0.101 ± 0.001
E1-β-2DG (2c)	0.968 ± 0.026
E2-β-Glc (3a)	0.266 ± 0.017
E2-α-2DG (3b)	0.159 ± 0.009
E2-β-2DG (3c)	0.413 ± 0.084
